# Attenuation of Innate Immunity by Andrographolide Derivatives Through NF-κB Signaling Pathway

**DOI:** 10.1038/s41598-017-04673-x

**Published:** 2017-07-05

**Authors:** Xin Nie, Shao-Ru Chen, Kun Wang, Yuran Peng, Yi-Tao Wang, Decai Wang, Ying Wang, Guo-Chun Zhou

**Affiliations:** 1 School of Pharmaceutical Sciences, Nanjing Tech University, Nanjing, Jiangsu 211816 China; 2 State Key Laboratory of Quality Research in Chinese Medicine and Institute of Chinese Medical Sciences, University of Macau, Avenida da Universidade, Taipa, Macao SAR China

## Abstract

Andrographolide derivatives or analogs exhibit potent anti-inflammatory effects in several disease models through NF-κB activity. In this study, we synthesized different andrographolide derivatives and investigated their effects on the toll-like receptor (TLR)-induced production of pro-inflammatory cytokines. Among these compounds, **3b**, **5a**, and **5b** inhibited both TNF-α/NF-κB and TLR4/NF-κB signaling pathways. Treatment with compounds **3b**, **5a**, and **5b** and their structural analogs, **3a** and **6b**, suppressed the expression of pro-inflammatory cytokines upon the activation of TLR3 and TLR4 ligands. Compounds **3b** and **5a**, but not **3a**, **5b**, or **6b**, inhibited the nuclear translocation of the NF-κB p65 subunit. Treatment with compounds **3b**, **5a**, **3a**, **5b**, and **6b** attenuated the phosphorylation of p65 and IκBα. Compounds **6b** suppressed the expression of the NF-κB p65 subunit. However, these compounds, except for **5b**, did not affect the TLR9-induced NF-κB-independent production of the pro-inflammatory cytokines, TNF-α, and IFN-β. Compound **3b** potentially protected mice from LPS-induced acute pulmonary inflammation through the inhibition of p65 phosphorylation and the decrease of serum pro-inflammatory cytokines and chemokine. Our study revealed a functional structure–activity relationship between andrographolide derivatives and innate immunity. We identified compound **3b** as a potent immune suppressive agent with the potential to protect acute pulmonary infection.

## Introduction

**Figure 1:**
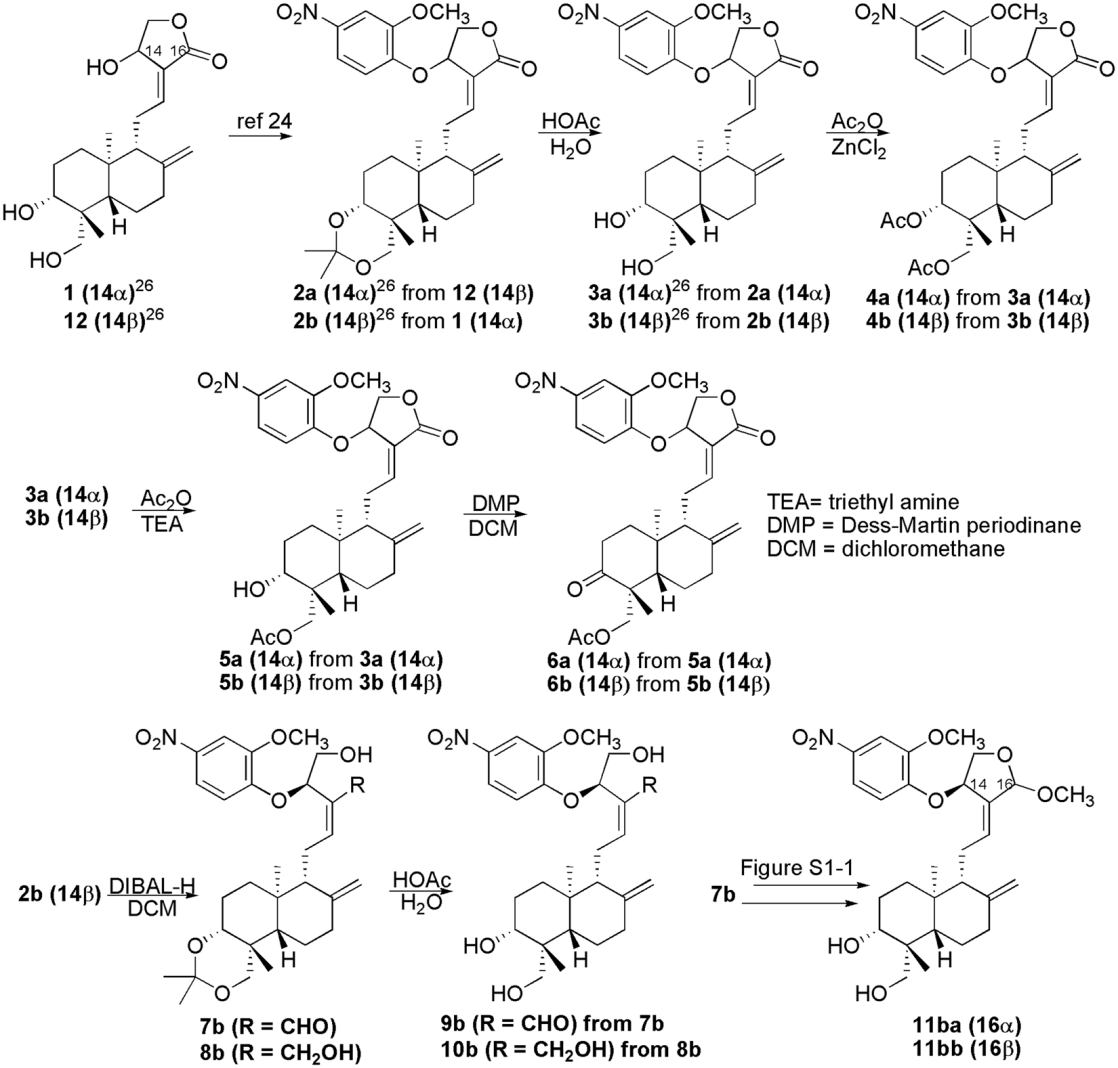
Synthesis of andrographolide derivatives.

Andrographolide (**1**, Fig. 1) is the active component of the medicinal plant *Andrographis paniculata* Nees (Acanthaceae)^1^. A. *paniculata* Nees is traditionally used in China, India, and Thailand in removing heat and toxic materials^1^. Andrographolide has been employed to treat inflammation- and oxidative stress-related diseases, including diarrhea^2^, rheumatoid arthritis^3^, and chronic rhinosinusitis with nasal polyps^4^. Andrographolide treatment reduces serum cholesterol, triglycerides, and low-density lipoprotein cholesterol in hyper-cholesterolemic patients and animals fed with high-fat diets^5^. Andrographolide treatment decreases hepatic neutrophil/macrophage infiltration, down regulates local inflammation, and reduces liver damage in thioacetamide-induced mouse hepatic fibrosis^6^. The anti-inflammatory effect of andrographolide is induced by inhibiting the NF-κB signaling pathway^7–9^. Mass spectrometry result and molecular docking analysis revealed that andrographolide binds to the NF-κB p50 subunit at Cys62 position^10^. Andrographolide has potent inhibitory effect to the NF-κB signaling pathway in several disease models, including TNBS-induced colitis mouse model^11^, lipopolysaccharide (LPS)-induced acute lung injury^9^, and endometriosis^12^.

Several andrographolide derivatives have been synthesized to improve the potency and selectivity of drugs. The andrographolide derivatives isoandrographolide, 7-*O*-methylwogonin, and skullcapflavone-I significantly inhibit the release of NO and prostaglandin E2 and the production of interleukin (IL)-1β and IL-6 in LPS-stimulated J774A.1 macrophage cells in a dose dependent manner^13^. AL-1, an andrographolide analog, improves insulin resistance by down regulating the NF-κB signaling pathway^5^. Treatment with 40 mg/kg and 80 mg**/**kg AL-1exhibits a significant hypoglycemic effect by reducing cholesterol levels and increasing high density lipoprotein levels, respectively^5^. At 1 μM, AL-1 suppresses the high-glucose-induced phosphorylation of the NF-κB p65 subunit and IκBα in rat-islet-derived insulinoma cells. In this case, homeostatic assessment shows that insulin resistance was reduced, and insulin sensitivity was restored^5^. Andrographolide suppresses toll-like receptor 4 (TLR4) expression and NF-κB signaling in multiple myeloma cells at 10 μM^14^. However, the suppression of TLR4 and NF-κB possibly resulted from andrographolide cytotoxicity against the cell model under conditions specified by the experiment^14^.

TLRs are involved in the regulation of innate and adaptive immunity, and non-infectious inflammatory liver diseases^15^. For example, TLR4 acts as a receptor for LPS, which is a cell-wall component of Gram-negative bacteria that induces strong inflammation^15^. TLR4 is closely related to lung infection^15^ and hepatocellular carcinoma^16^. Intracellular TLRs sense foreign genomic material: TLR3 detects viral double-stranded RNA^17^, TLR7 recognizes single-stranded RNA upon viral infection^18^, and TLR9 responds to specific unmethylated CpG motifs in microbial genomic DNA^19^. TLR hyperactivation by corresponding ligands generates a cytokine storm that ultimately mediates acute tissue injury^20–22^. The MyD88/NF-κB/JNK complex is an important collection of adaptor molecules that links receptor-initiated signals to transcriptional regulation in the nucleus^21^. Innate immune responses to several TLRs triggered by ligands also share this highly conserved signaling pathway. Therefore, targeting the NF-κB signaling pathway may elucidate TLR-initiated innate immunity, such as acute pulmonary injury^23^.

In the current study, we synthesized a set of andrographolide derivatives (Fig. 1) to explore new conceptual compounds with higher rigidity, which possibly restricts the rotation of the bonds of C9-C11 and C11-C12 and lowers the competence as Michael acceptor^24^. We then determined the specificity of these andrographolide derivatives against NF-κB-dependent and -independent TLR signaling pathways. One major function of andrographolide is to treat respiratory infection and inflammation^25^. We also evaluated the protective effectiveness of active andrographolide derivative on LPS-induced acute pulmonary inflammation. We aimed to determine the structural basis for the improved potency and immune-suppressive effect of andrographolide. We proposed the structure-activity relationship of andrographolide derivatives against NF-κB signaling pathway, and determined the therapeutic potential in LPS-triggered acute pulmonary injury.

## Results and Discussion

Compounds of listed andrographolide derivatives were synthesized as shown in Fig. 1 and S1 (Supporting information). First, to obtain the aromatic derivatives **2a** or **2b**, 4-nitro-2-methoxyphenol was introduced into the 14-position of andrographolide (**1**) or its 14-epimer (**12**) via the Mitsunobu reaction in a process modified from our previous report^26^. The 3,19-diol derivatives **3a** and **3b** resulted from deprotecting 3, 19-acetonylidene from **2a** and **2b**. Anhydrous ZnCl_2_ catalyzed the di-acetylation of **3a** and **3b** to yield **4a** and **4b**, respectively. Meanwhile, the triethylamine-catalyzed mono-acetylation of **3a** and **3b** formed the 19-acetylated products **5a** and **5b**, respectively. The 3-alcohols of **5a** and **5b** were oxidized by Dess-Martin periodinane into the corresponding 3-ketones, **6a** and **6b**, respectively. Reducing the lactone moiety of **2b** yielded a semi-reduction product of semi-acetal **7b** and a fully reduced product of alcohol **8b**. Deprotection of 3,19-acetonylidene from **7b** and **8b** yielded **9b** and **10b**, respectively. Semi-acetal **7b** was transformed into the diastereomeric acetals of **11ba** and **11bb** (Figure S1), which was inseparable by routine separation methods. Diastereoisomeric separation of **11ba** and **11bb** was performed through the 3,19-disilylated modification of **11ba** and **11bb** to form separable **12ba** and **12bb**, respectively, followed by the deprotection of 3,19-disilylation from **12ba** and **12bb**, respectively (Figure S1).

We utilized luciferase reporters bearing the TNF-α/NF-κB, TLR4/NF-κB, and interferon-γ activated signal (GAS, Table 1) to examine the activity of the newly synthesized andrographolide analogs on signaling pathways that govern the inflammatory response. Inhibiting TLR4 signaling has received considerable attention in the regulation of innate immune response. LPS-activated TLR4 regulates the expression and translocation of NF-κB^27^. Interferon-γ (IFN-γ) helps in activating anti-microbial and anti-tumor lymphocytes to regulate the proliferation and differentiation of biomolecules^28^. The inhibition of IFN-γ signaling regulates cytokine expression through the IFN-γ-STAT3-GAS signaling pathway. These derivatives did not show detectable cytotoxicity against both AD-293 and HeLa cell lines. Parent compound **1** did not show any influence against these signaling pathways up to 10 μM in AD-293 and HeLa cells (Table 1). Meanwhile, none of these derivatives influenced active against the IFN-γ signaling pathway. The 14α-compounds of **2a** and **3a** inhibited TLR4/NF-κB, but not TNF-α/NF-κB in both AD-293 and HeLa cell lines. The 14β-compound **2b** influenced TLR4 and only affected the NF-κB in HeLa cells. 14β-Compound **3b** showed a similar effect on TLR4 to **2b**; however, but the inhibitory effect of the former against NF-κB improved to lower than 10 μM in both cell lines. The latter result indicates that removing 3,19-acetonylidene facilitates the NF-κB inhibition from **2b** to **3b**. Di-acetylation of **3a** and **3b** generated diverse results of the corresponding **4a** and **4b** products. In particular, **4a** selectively exerted its activity against NF-κB in the AD-293 cell line; however, **4b** influenced NF-κB only in the HeLa cell line. Furthermore, results indicated that **4a** was the most active compound against TLR4, followed by **4b**. Unlike the di-acetylation substances, mono-acetylated **5a** and **5b** exhibited good activity against TLR4 and NF-κB in the two cell lines. **5a** and **5b** suppressed TLR4 to similar levels; however, **5b** had slightly more active inhibitory effect against NF-κB than that of **5a**. The oxidization of 3-alcohol **5a** into ketone **6a** enhanced the inhibition of TLR4 expression and suppression of NF-κB signaling in the AD-293 cell line. Oxidation also removed the inhibitory effect on NF-κB in the HeLa cell line. Compound **6b** did not active influence NF-κB in both cell lines and had reduced effect on TLR4 relative to 3-alcohol **5b**. The semi-acetal **7b** only affected the NF-κB in the HeLa cell line. Unfortunately, modifying compounds **8b**, **9b**, **10b**, **11ba**, and **11bb** completely reduced lactone, thereby removing their inhibitory effect on NF-κB and TLR4. All of the lactone-modified derivatives of **7b**, **8b**, **9b**, **10b**, **11ba**, and **11bb** analogs were inactive in all of the tests, although all of the other derivatives potently inhibited the TLR4 signaling pathway in the AD-293 cells (Table 1). Therefore, this work identified the lactone moiety as the key structural motif in the interaction between the test compound and its drug target(s). Notably, the 14α-isomers of **4a** and **6a** influenced NF-κB signal transduction more in the AD-293 cell line than in the HeLa cell line. The 14β-isomers of **2b**, **4b**, and **7b** also selectively inhibited the NF-κB signaling pathway in the HeLa cell line. Overall, among all of the tested derivatives, the NF-κB signaling pathway in the AD-293 cells was only potently inhibited by the 4-h treatment of **3b**, **5b**, and **5a** at EC_50_ concentrations of approximately 10 μM in AD-293 cells (Table 1). These derivatives were also sensitive to the NF-κB signaling pathway in HeLa cells (Table 1), thereby suggesting that the inhibitory activities of these compounds are unaffected by the cellular background.

**Table 1:** The EC_50_ (half effective concentration) of all compounds against NF-κB, TLR4, and IFN-γ signaling pathways and the cytotoxicity (CC_50_, half cytotoxic concentration) in AD-293 and Hela cells.

cmpd	EC_50_ in AD-293 (μM)				EC_50_ in Hela (μM)	
	NF-κB^a^	TLR4^b^	IFN-γ^c^	CC_50_^d^	NF-κB^a^	CC_50_^d^
Andrographolide (**1**)	>10	>10	>10	>10	>10	>10
**2a**	>10	5.76 ± 1.40	>10	>10	>10	>10
**2b**	>10	3.35 ± 0.36	>10	>10	6.22 ± 0.60	>10
**3a**	>10	5.36 ± 0.58	>10	>10	>10	>10
**3b**	8.97 ± 1.03	3.49 ± 0.34	>10	>10	2.00 ± 0.66	>10
**4a**	8.85 ± 1.06	0.90 ± 0.14	>10	>10	>10	>10
**4b**	>10	3.32 ± 0.72	>10	>10	5.48 ± 0.39	>10
**5a**	10.37 ± 0.71	5.79 ± 0.96	>10	>10	4.89 ± 0.28	>10
**5b**	7.54 ± 1.33	5.98 ± 0.30	>10	>10	2.31 ± 0.60	>10
**6a**	7.92 ± 0.42	3.55 ± 0.07	>10	>10	>10	>10
**6b**	>10	7.67 ± 0.67	>10	>10	>10	>10
**7b**	>10	>10	>10	>10	5.48 ± 0.34	>10
**8b**	>10	>10	>10	>10	>10	>10
**9b**	>10	>10	>10	>10	>10	>10
**10b**	>10	>10	>10	>10	>10	>10
**11ba**	>10	>10	>10	>10	>10	>10
**11bb**	>10	>10	>10	>10	>10	>10
**12ba**	>10	>10	>10	>10	>10	>10
**12bb**	>10	>10	>10	>10	>10	>10

^a^Treated for 4 h.

^b^Treated for 16 h.

^c^Treated for 24 h.

^d^Treated for 24 h.

**Figure 2:**
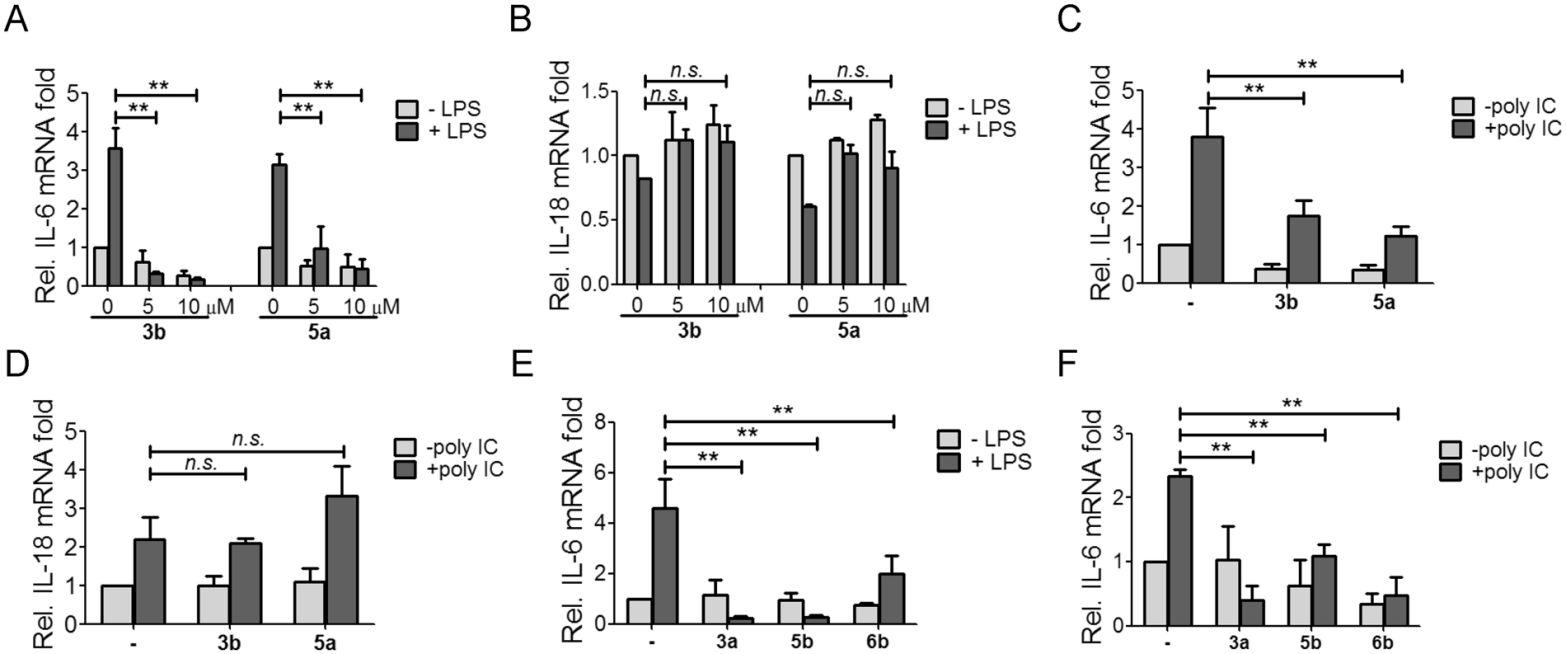
Andrographolide derivatives inhibited proinflammatory cytokine transcription. THP-1 cells differentiated with 10^−8^ M PMA were treated with 1 μg/ml LPS in the presence or absence of **3b** and **5a** for 6 h. The mRNA levels of (**A**) IL-6 and (**B**) IL-18 were determined by real-time PCR analysis. THP-1 cells were transfected with 50 ng/μl poly I:C using PEI in the presence or absence of **3b** and **5a** for 9 h. The mRNA levels of (**C**) IL-6 and (**D**) IL-18 were determined by real-time PCR analysis. (**E**) THP-1 cells were treated with 5 μg/ml LPS in the presence or absence of 10 μM **3a**, **5b**, and **6b** for 6 h, or (**F**) THP-1 cells were transfected with 50 ng/μl poly I:C in the presence or absence of 10 μM **3a**, **5b**, and **6b** for 9 h, the mRNA level of IL-6 was determined by real-time PCR analysis. Results are presented as mean ± SD from at least three independent experiments (**p < 0.05; *n.s*., non-specific; −, DMSO control).

We then utilized the TLR4 ligand LPS to induce inflammatory responses in THP-1 cells. The LPS-stimulated transcription of pro-inflammatory cytokine IL-6 was suppressed with **3b** and **5a** treatments at 5 and 10 μM (Fig. 2A), respectively. However, IL-18 transcription was unchanged (Fig. 2B). Treatment with **3b** and **5a** abrogated the transcription of polyinosinic:polycytidylic acid (poly I:C)-induced IL-6 (Fig. 2C). Upon poly I:C stimulation, **5a** treatment enhanced IL-18 mRNA, which remained unchanged with **3b** treatment (Fig. 2D). This result was consistent with a previous report that IL-18 transcription was unrelated to TLR stimulation^29^. Treatment with **3a**, **5b**, and **6b** also inhibited LPS or poly I:C-stimulated transcription of IL-6 (Fig. 2E,F).

**Figure 3:**
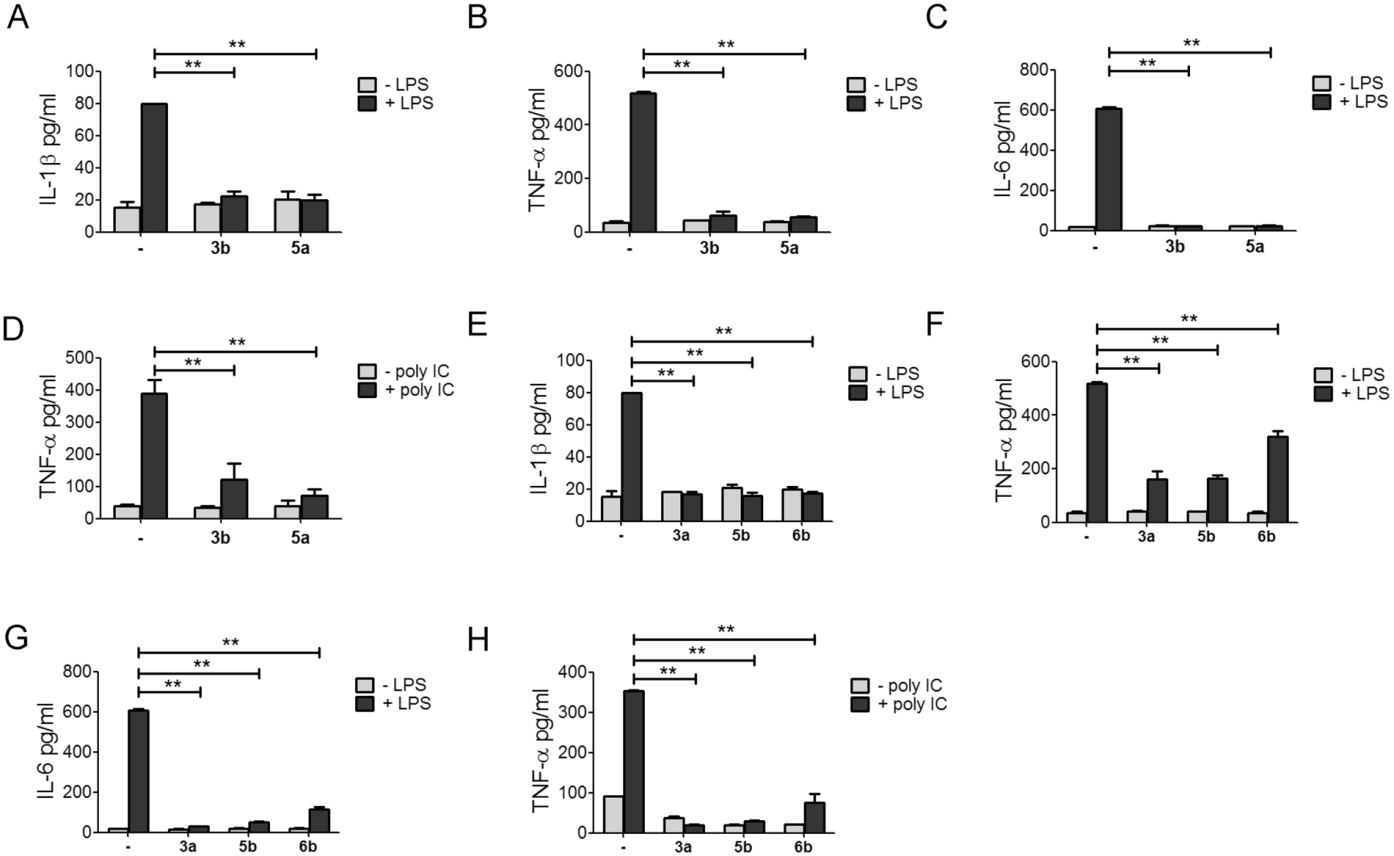
Andrographolide analogs inhibited pro-inflammatory cytokines expression. THP-1 cells differentiated with 10^−8^ M PMA were treated with 1 μg/ml LPS in the presence or absence of **3b** and **5a** for 6 h. The levels of secreted (**A**) IL-1β, (**B**) TNF-α, and (**C**) IL-6 in the medium was determined by LEGENDplex^TM^ bead-based immunoassays. (**D**) THP-1 cells were transfected with 50 ng/μl poly I:C in the presence or absence of **3b** and **5a** for 9 h. The level of secreted TNF-α in the medium was determined as in (**B**). Effects of **3a**, **5b**, and **6b** on the LPS-stimulated secretion of (**E**) IL-1β, (**F**) TNF-α, and (**G**) IL-6 were determined as in (**A**). (**H**) Effects of **3a**, **5b**, and **6b** on the poly I:C-stimulated secretion of TNF-α were determined as in (**A**). Results are presented as mean ± SD of three different experiments (**p < 0.05; −, DMSO control).

To better understand the effect of andrographolide derivatives on TLR-triggered innate immune responses, we further analyzed the cytokines released into the medium under different stimuli. Treatment with **3b** and **5a** significantly inhibited the LPS-stimulated release of pro-inflammatory cytokines, including IL-1β, TNF-α, and IL-6 (Fig. 3A–C), as well as poly I:C-triggered TNF-α expression (Fig. 3D). Treatment with **3a**, **5b**, and **6b** suppressed the LPS-stimulated expression of IL-1β, TNF-α, and IL-6, respectively (Fig. 3E–G). However, their inhibitory effect against TNF-α and IL-6 expression (Fig. 3F) was less than that of **3b** and **5a** (Fig. 3B,C). Compounds **3a**, **5b**, and **6b** inhibited poly I:C-triggered TNF-α under the same conditions (Fig. 3H). Compounds **3a** and **5b** expressed more inhibitory activity against poly I:C-induced TNF-α than compounds **3b**, **5a**, and **6b** (Fig. 3D–H). The NF-κB signaling pathway was indirectly inhibited by **3a** and **6b** (Table 1). These compounds exerted their inhibitory activity through TLR4 signal transduction.

**Figure 4:**
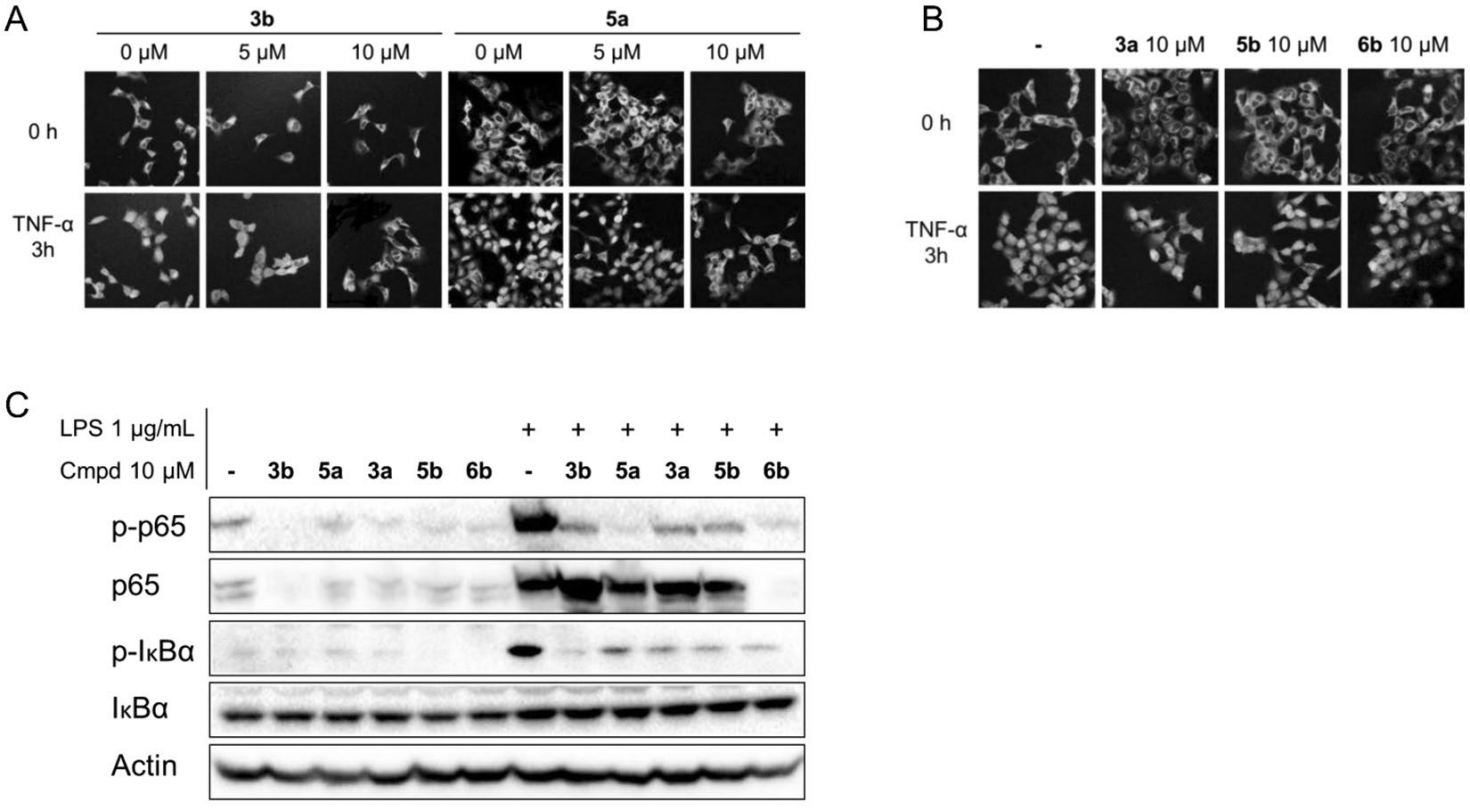
Treatment with andrographolide analogs inhibited the NF-κB signaling pathway. AD-293 cells were treated with 50 ng/ml TNF-α alone or in combination with either (**A**) **3b** and **5a**, or (**B**) **3a**, **5b**, and **6b** for 3 h. After fixation, the nuclear translocation of NF-κB p65 was examined under a confocal microscope. Images were obtained under 200× magnificence. (**C**) Western blot analysis of the levels of phosphorylated p65, p65, phosphorylated IκBα, and IκBα treated with 1 µg/ml LPS alone or in combination with compounds **3b**, **5a, 3a**, **5b**, and **6b** in THP-1 cells. The blot of actin was used as an internal loading control. The results are representative of three independent experiments.

In several diseases, the activation of the NF-κB signaling pathway governs the transcription of pro-inflammatory cytokines, including IL-6, IL-1β, and TNF-α^30^. NF-κB activation depends on the phosphorylation-triggered proteasome degradation of inhibitor IκB proteins. The IκB kinase complex, which is responsible for IκB phosphorylation, is essential to the nuclear translocation of NF-κB and the initiation of subsequent signal transduction^30^. To confirm the inhibitory activity of andrographolide derivatives against NF-κB signaling, we determined the effect of these analogs on the nuclear translocation of NF-κB in AD-293 cells using immunofluorescent staining. Treatment with **3b** and **5a** inhibited the TNF-α-stimulated nuclear translocation of p65 at 10 μM (Fig. 4A). By contrast, structural analogs **3a**, **5b**, and **6b** did not show apparent activity at 10 μM (Fig. 4B). Western blot analysis revealed that all the compounds decreased LPS-triggered phosphorylation of p65 and IκBα in THP-1 cells (Fig. 4C). Compounds **3b** and **3a** enhanced the expression level of p65 as compared with LPS alone (Fig. 4C). Compound **6b** inhibited the endogenous expression level of p65 subunit (Fig. 4C). LPS-stimulated expression of IκBα was affected by none of the compounds (Fig. 4C).

**Figure 5:**
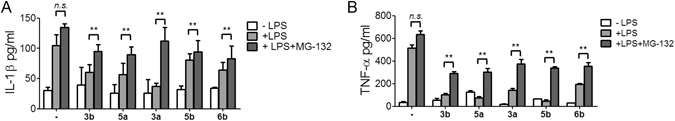
Effects of proteasome inhibitor MG-132 on the inhibition of TLR4 signaling induced by andrographolide derivatives. THP-1 cells differentiated with 10^−8^ M PMA were treated with 1 μg/ml LPS and pretreated with 1 μg/ml MG-132 16 h before the addition of andrographolide analogs. The levels of (**A**) IL-1β and (**B**) TNF-α in the medium were determined by LEGENDplex^TM^ bead-based immunoassays after treatment with compounds **3b**, **5a**, **3a**, **5b**, and **6b** for an additional 6 h. The results are representative of at least three independent experiments and presented as mean ± SD of three different experiments (**p < 0.05; −, DMSO control).

**Figure 6:**
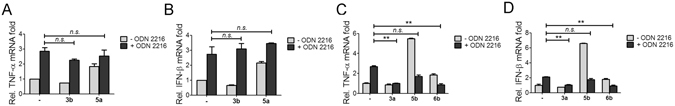
Effects of andrographolide derivatives on class A CpG ODN2216-stimulated TLR9 signaling. AD-293 cells were stimulated with ODN2216 alone or combined with andrographolide derivatives. The mRNA levels of TNF-α and IFN-β treated with (**A**) and (**B**) **3b** and **5a**, or (**C**) and (**D**) **3a**, **5b**, and **6b**, respectively, were quantitated by real-time PCR. The results are representative of at least three independent experiments and presented as mean ± SD (**p < 0.05; *n.s*., nonspecific).

Proteasome inhibitor MG 132 abrogates the activation of the NF-κB signaling pathway by blocking IκB degradation^31^. In Fig. 5, the LPS-triggered expression of IL-1β and TNF-α slightly increased in the presence of MG132 compared with LPS treatment alone. This condition may result from the activation of other signaling mediators, such as activator protein-1, which controls the transcription of pro-inflammatory cytokines^31^. Pretreatment with MG132 significantly decreased the suppressive effect of the andrographolide derivatives **3b**, **5a**, **3a**, **5b**, and **6b** on LPS-stimulated IL-1β and TNF-α production (Fig. 5A,B). The strongest counteractions were exerted by **3a** against IL-1β production and by **5b** against TNF-α production. Thus, **3a** and MG132 directly competed for IL-1β production, whereas **5b** and MG132 competed directly for TNF-α production. Synthetic CpG oligonucleotides (ODNs) contain unmethylated CpG dinucleotides and are present in bacterial DNA at a higher frequency than in mammalian DNA. Class A CpG ODN 2216 induces the TLR9-dependent activation of pro-inflammatory cytokines by weakly stimulating NF-κB signaling^30^. Treatment with compounds **3b**, **5a**, and **3a** did not inhibit ODN 2216-induced TNF-α and IFN-β transcription (Fig. 6). By contrast, treatment with **5b** and **6b** strongly enhanced the transcription of TNF-α and IFN-β cytokines, respectively (Fig. 6C,D). These results show a similar effect on LPS-stimulated TNF-α cytokine levels (Figs 3F,H and 5B).

**Figure 7:**
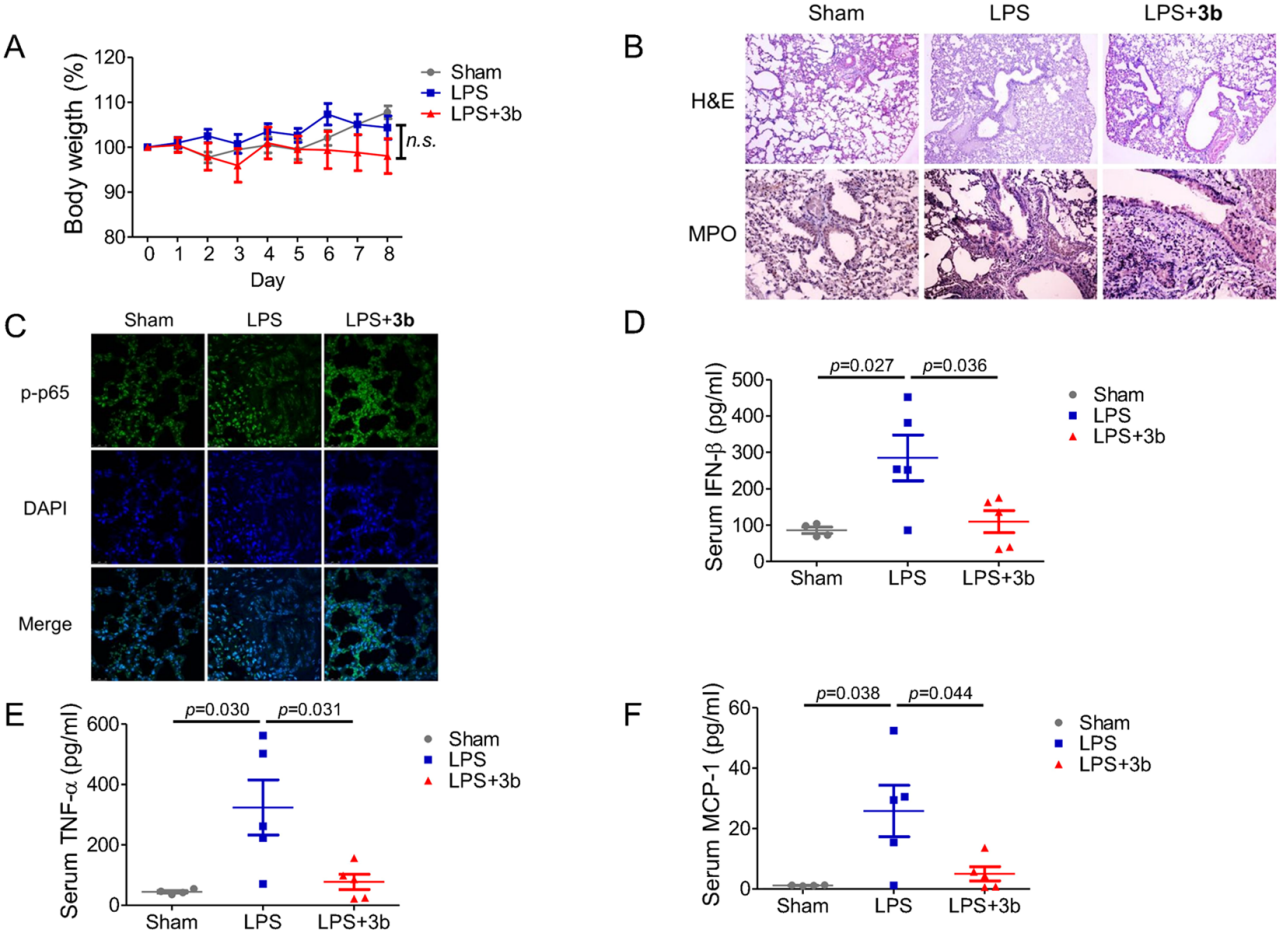
Efficacy of compound **3b** (45 mg/kg) on LPS-induced pulmonary injury. (**A**) Body weight changes of mice with LP-induced pulmonary injury. (**B**) H&E staining and immunohistochemistry analysis of MPO of mice lung tissue (630× magnification). (**C**) Immunofluorescent analysis of phosphorylated p65 level in mice lung tissue (400× magnification). Serum levels of (**D**) IFN-β, (**E**) TNF-α, and (**F**) MCP-1 were determined by LEGENDplex^TM^ bead-based cytokine and chemokine immunoassays. The results are representative of two independent experiments (n ≥ 4 for each group) and presented as mean ± SD (*p* value was labeled on the figure; *n.s*., nonspecific).

We further evaluated the therapeutic potential of active andrographolide derivative on LPS-induced acute pulmonary injury in mice (Fig. 7). Oral administration of compound **3b** did not significantly change the body weight during the treatment (Fig. 7A). Hematoxylin and eosin (H&E) staining results indicated that compound **3b** decreased LPS-induced alveolar wall thickening (upper panel, Fig. 7B). Neutrophil infiltration is an indicator of lung injury^32^. We then determined neutrophil infiltration by immunohistochemistry staining of myeloperoxidase (MPO), which is most abundantly expressed in neutrophils^32^. The number of MPO-positive cells were significantly increased in the LPS-treated group, whereas compound **3b** decreased MPO-positive cells (lower panel, Fig. 7B). Phosphorylated p65 was mainly localized in the nucleus in the LPS-treated group, whereas compound **3b** led to the cytosolic localization of phosphorylated p65 (Fig. 7C). The expression level of phosphorylated p65 was undetectable by Western blot analysis (Fig. 4C); however, fluorescent staining is not a quantitative method, and fluorescent intensity shown in different treatment could not reflect protein expression levels. LPS-induced elevation of serum levels of pro-inflammatory cytokines and chemokine, including IFN-β, TNF-α, and MCP-1, were significantly decreased by compound **3b** treatment (Fig. 7D–F). These results suggested that compound **3b** protected LPS induced pulmonary injury through the inhibition of NF-κB signaling *in vivo*.

## Conclusion

In summary, we identified several andrographolide derivatives as specific NF-κB inhibitors (Table 1). SARs indicated that in addition to the importance of the good pharmacophore of 4-nitro-2-methoxyphenoxy, the derivative’s inhibitory potency and selectivity of a derivative are determined by 14-stereochemistry, 13-monoacetylation, 3,19-diacetylation, 3-alcohol, or 3-ketone. Among these andrographolide derivatives, **3b** is a potent and specific NF-κB inhibitor that prevents the phosphorylation of the NF-κB p65 subunit without affecting the endogenous expression of NF-κB family members. In this report, we identified compounds **3b**, **5a**, **5b**, and **6b** as potential immunomodulatory inhibitors of TLR signaling with distinct regulation of NF-κB family members. Compound **3b** effectively reduced LPS-induced pulmonary injury by decreasing the nucleus translocation of phosphorylated p65 and serum pro-inflammatory cytokines. The andrographolide derivative **3b** may serve as a useful framework in further developing potential agents for the treatment of inflammatory and autoimmune diseases, and immunotherapy against various pathogens, such as bacteria-induced acute pulmonary injuries.

## Materials and Methods

### Reagents

Cell culture media and fetal bovine serum (FBS) were purchased from Gibco. Lipopolysaccharide (LPS), polyinosinic:polycytiylic acid (poly I:C), and ODN 2216 were purchased from InvivoGen (San Diego, CA). TNFα was purchased from PeproTech (Rocky Hill, NJ). Chemical compounds were dissolved in DMSO at 10 mM as store solution. The content of DMSO doesn’t exceed 0.1% in cells. MG132 was purchased from MedChem Express (Monmouth Junction, NJ, USA). All other reagents were purchased from Sigma-Aldrich (St. Louis, MO) except for those otherwise noted.

### Cell culture condition

Cell lines were obtained from the American Type Culture Collection (ATCC). HeLa and AD-293 cells were maintained in DMED high glucose medium supplemented with 10% FBS. Hela and AD-293 stable cell lines harboring NF-κB and GAS response element in pGL4.20 vector (Promega) were maintained in the presence of 1 μg/ml puromycin. AD-293 cells with overexpression of TLR4 and NF-κB response element plasmid was maintained in the presence of 1 μg/ml puromycin and 10 μg/ml blasticidin. HEK-293 cells with overexpression of TLR9 were maintained in the presence of 10 μg/ml blasticidin.

THP-1 cells were maintained in RPMI-1640 medium with 10% FBS plus 0.05 M 2-mercaptoethanol. The differentiation of THP-1 monocytes into macrophages-like cells was triggered by 10^−8^ M phorbol 12-myristate 13-acetate (PMA) for 72 hours. All cell lines were maintained in a humidified incubator with an atmosphere of 95% air and 5% CO_2_ at 37 °C.

### Reporter assay

Reporter cells were treated with 50 ng/ml TNF-α to stimulate NF-κB signaling pathway, 20 pg/ml IFN-γ to stimulate GAS signaling pathway, and 1 μg/ml LPS to stimulate TLR 4. Medium was removed at the end of the treatment, and cell extracts were prepared and luciferase activity was measured by Luciferase assay kit (Promega) according to the manufacturer’s instructions. EC_50_ was defined as the concentration of drug that inhibited stimulator-triggered luciferase reporter activation by 50% after continuous drug exposure for 4 hours.

### Western blot

Western blot was performed according to a standard protocol. The primary antibodies using in this research are from NFκB pathway sampler kit (CST#9936), including anti-p-IκBα, anti-IκBα, and anti-NFκB p65. GAPDH (Santa Cruz, #sc-32233) is chosen as housekeeping protein.

### qRT-PCR

RNA was extracted from THP1 cells using Trizol (Invitrogen). And total RNA was reversely transcribed using ProtoScript®II Reverse Transcriptase (NEB). Quantitative PCRs were performed triplicates for all samples using the Mx 3005P Real-Time PCR system (Stratagene) with SYBR Green I nucleic acid gel stain (Life Technologies). For normalization, ΔC_t_ values were calculated relative to the levels of Actin transcripts. Primers for qRT-PCR were listed in Table S1.

### Immunofluorescent

Immunofluorescent was performed for TNF-α-stimulated nucleus translocation of NF-κB. The primary antibody used was anti-NFκB p65 (1:100 dilution; CST #8242). Secondary antibody is anti-rabbit IgG HRP-linked antibody (1:1000 dilution; CST #7074). Nuclei were revealed by DAPI staining. Fluorescence images were collected under confocal microscope system (Leica Microsystems, Wetzlar, Germany).

### Animals

Female WT BalB/C mice (18–20 g) were obtained from Animal Facility at University of Macau. All methods were carried out in accordance with relevant guidelines and regulations. All experimental protocols were approved by Panel on Animal Research Ethics of University of Macau. Mice were acclimated for 1 week prior to use in experiments with free access to water and chow diet through the experiment. *In vivo* experiments were performed in the animal facility.

### Treatment conditions

BABL/c mice were randomly divided into three groups, sham, LPS and LPS plus compound **3b** treatment group. Mice in the treatment group were pre-treated with compound **3b** (45 mg/kg) orally for 9 days, once daily. Mice in sham and LPS group were given PBS administration as placebo. All the mice were anesthetized with isofluorane suspended from incisors. PBS or LPS (200 μg/ml in PBS, 100 μg/kg) were administered intragastrically 1 h after the oral administration from day 7 to day 9. The intragastrically administration with LPS or PBS were given three times per day for three days continuously. Mice were sacrificed 6 h after the last intragastrically administration to collect serum and lung tissue.

### Collection of tissue and serum

Mice were sacrificed after being anaesthetized with CO_2_. Serum was collected and stored at −70 °C until analysis. Lung was removed, fixed and saved for histologic analysis.

### Histological analysis

After fixation, lung sections were stained with haematoxylin-eosin staining solution and examined under light microscopy. At least three different sections were examined per lung section.

### Immunohistochemistry and immunofluorescent

Lung sections were fixed with paraffin, and washed by PBS before block with 3% H_2_O_2_ solution. Paraffin-fixed lung sections were then washed by TBS-T and blocked in blocking buffer containing 10% goat serum, 0.1% BSA, 0.2% gelatin at room temperature for 1 hour. Lung sections were then incubated in primary antibody against MPO or phosphorylated p65 in blocking buffer at 4 °C overnight, washed by TBS-T and then incubated with HRP-conjugated secondary antibody. A set of slides were processed without incubation with primary antibody as negative control. The signal was detected by DAB peroxidase substrate kit (Vector Laboratories, Burlingame, CA, USA). The slides were counterstained with hematoxylin and mounted for immunohistochemistry analysis. The sections were incubated with FITC-conjugated secondary antibody and counterstained with DAPI for immunofluorescent analysis.

As a negative control, a set of slides was processed without primary antibody. To quantify stains of different proteins, pictures were taken of >30 fields of view at ×400 magnification. Adobe Photoshop CS2 software was used to pixel count the positive staining. The stains were scored by three researchers separately in blind.

### Statistical analysis

Data are presented as mean ± S.D. or mean ± S.E.M. No animals were excluded for analysis. All experiments were repeated two or more times. Data were normally distributed, and the variance between groups was not significantly different. Differences in measured variables between groups were analyzed by one-way or two-way ANOVA, or the student’s *t* test by GraphPad Prism 5 software. Results were considered statistically significant when *p* < 0.05.

### Supporting information

Experimental details for the syntheses, NMR spectra and HPLC purity analysis of listed compounds, and general information for biological evaluation. This material is available free of charge via the Internet.

### Data Availability

All data generated or analyzed during this study are included in this published article and its Supplementary Information files.

## Electronic supplementary material

Supplementary information
