# Supplementary material for: Attenuation of Innate Immunity by Andrographolide Derivatives Through NF-κB Signaling Pathway
Source: Sci Rep. 2017 Jul 5;7:4738. doi: 10.1038/s41598-017-04673-x (PMC5498490; doi:10.1038/s41598-017-04673-x)
Supplement: Supplementary file 1 — Supplementary information [file 41598_2017_4673_MOESM1_ESM.pdf]

Attenuation of Innate Immunity by Andrographolide Derivatives Through NF-κB Signaling Pathway

Xin Nie, Shao-Ru Chen, Kun Wang, Yuran Peng, Yi-Tao Wang, Decai Wang, Ying Wang, Guo-Chun Zhou

Contents:

Figure S1-1. Preparation and separation of diasteroisomers of **11ba** and **11bb** from **7b** --p 2

Determination of 16-stereochemistry of **12bb** by NOESY------------pp3-4

Figure S2. Original scan of Western blots-------------------------------page 5

Synthesis for Figure 1---pp6-15

Synthesis of Figure S1---pp16-19

NMR spectra--pp20-36

HPLC spectra--pp37-44

Biological evaluation---pp45-48

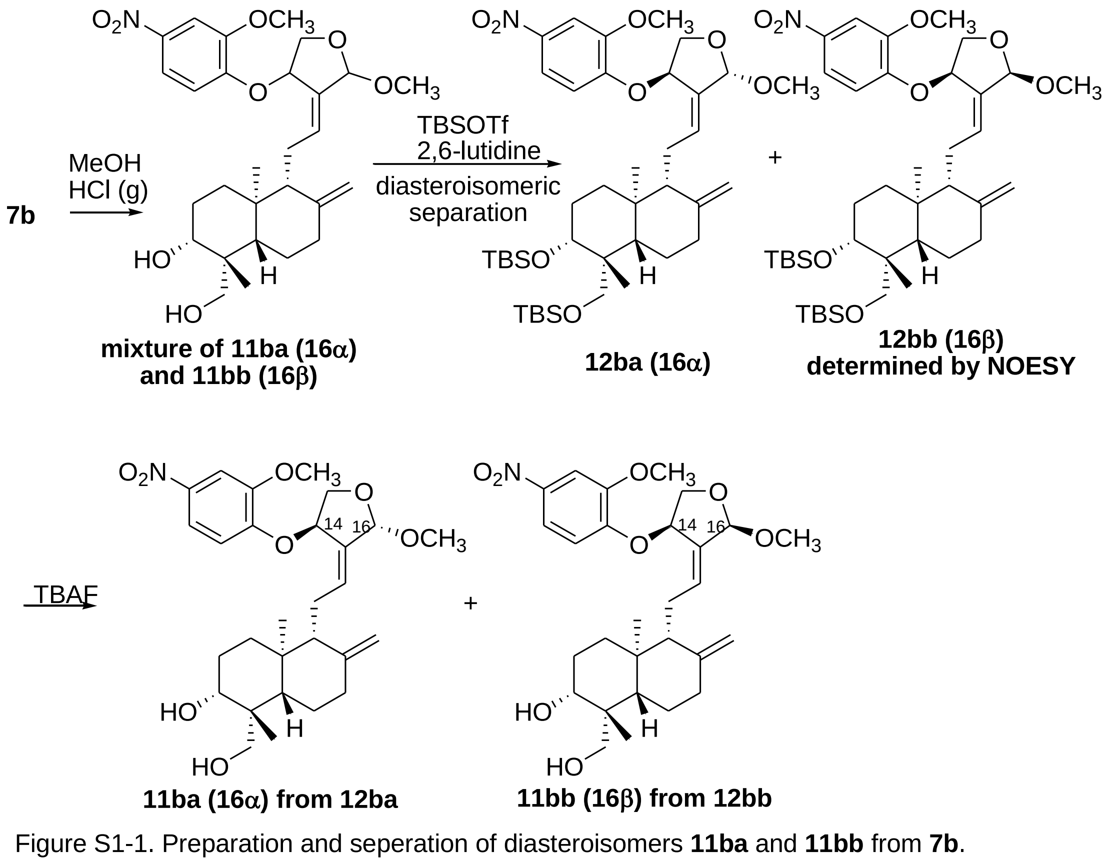

Figure S1-1. Preparation and separation of diasteroisomers of **11ba** and **11bb** from **7b**.

Determination of 16-stereochemistry of **12bb** by NOESY (Figures S1-2 and S1-3)

One $-OCH_3$ at 3.18 ppm is NOE correlated with aromatic H at 7.57 ppm, indicated this $-OCH_3$ is attached to benzene and another one at 3.28 ppm is acetal $-OCH_3$. The $-OCH_3$ at 3.28 ppm is NOE related with the signal at 5.22 ppm, which is NOE related with 12-H at 6.00 ppm, we conclude the signal at 5.22 ppm as 16-H. The signal of 12-H at 6.00 ppm is correlated with 16-H at 5.22 ppm, one proton of 17-H at 4.56 ppm and one of 11-H at 1.78 ppm. The proton at 5.01 ppm is strongly NOE with one of aromatic H at 6.22 ppm, weakly NOE with 16-H at 5.22 ppm, and very weakly NOE with 3.99 ppm, we can conclude 5.01 ppm signal is 14α-H and the stereochemistry is 14α-H and 16α-H. So, the absolute structure of 12bb is determined as Figure S1-2.

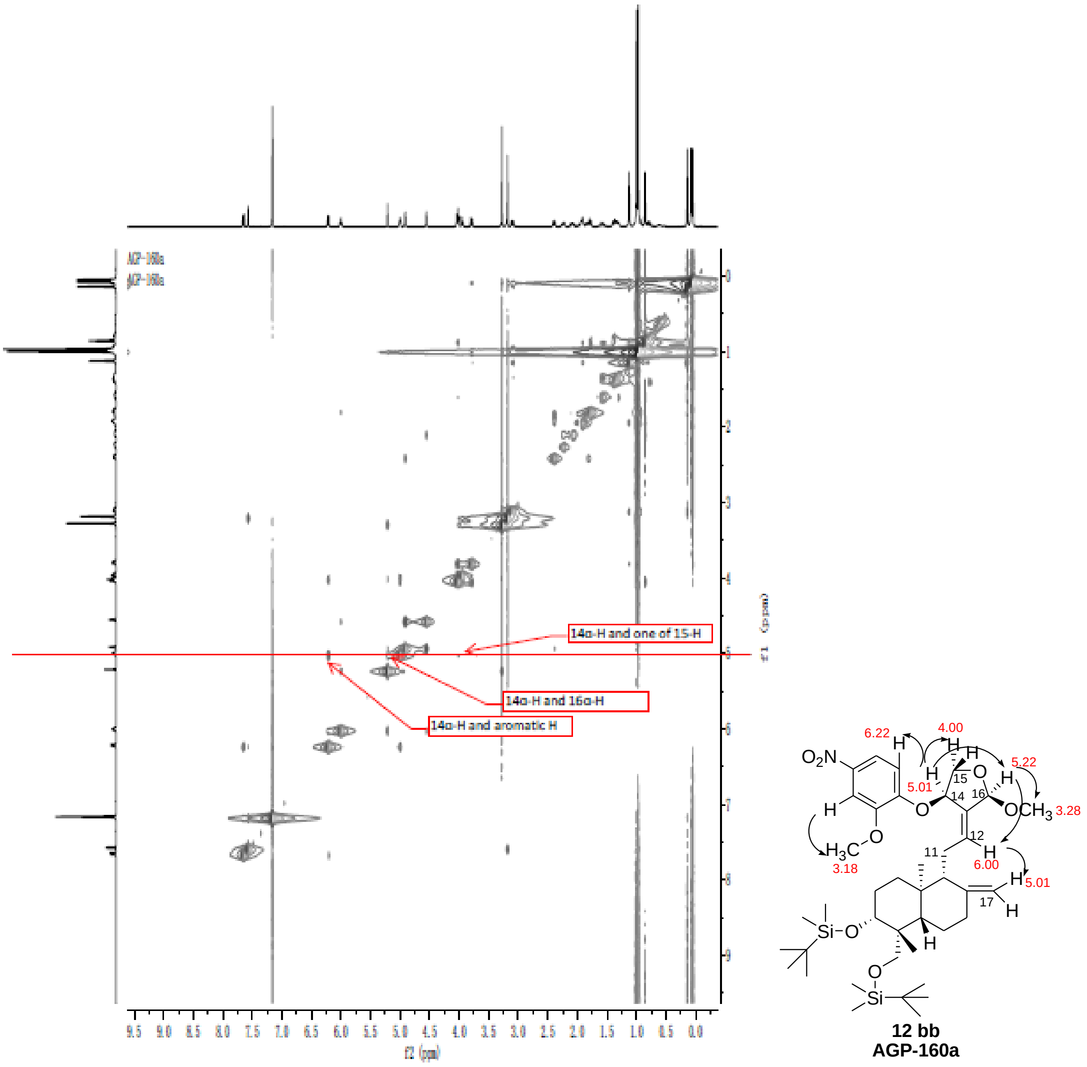

Figure S1-2 NOE relationship

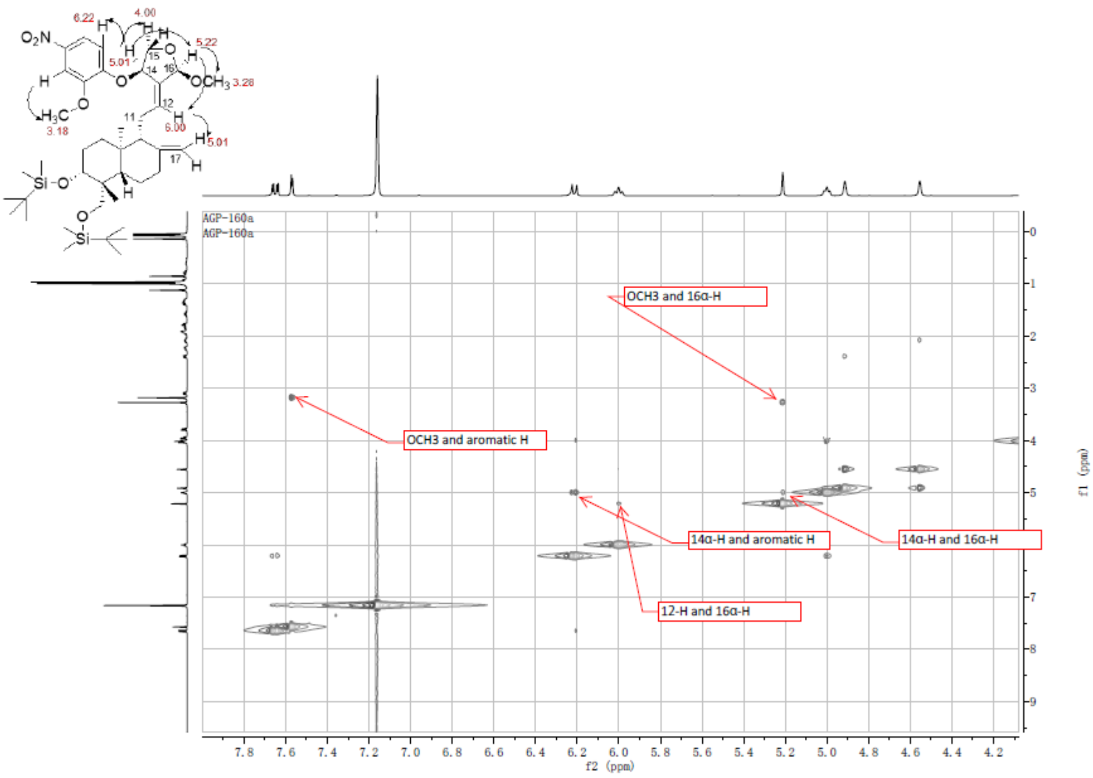

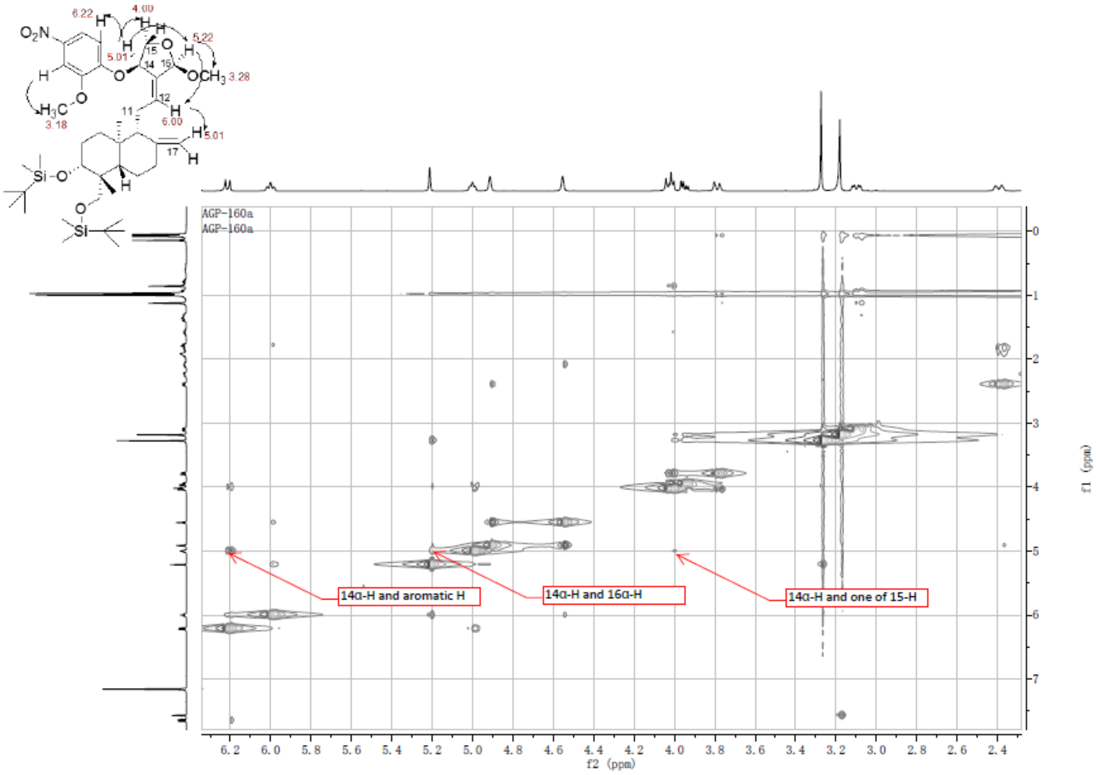

Figure S1-3 NOE relationship

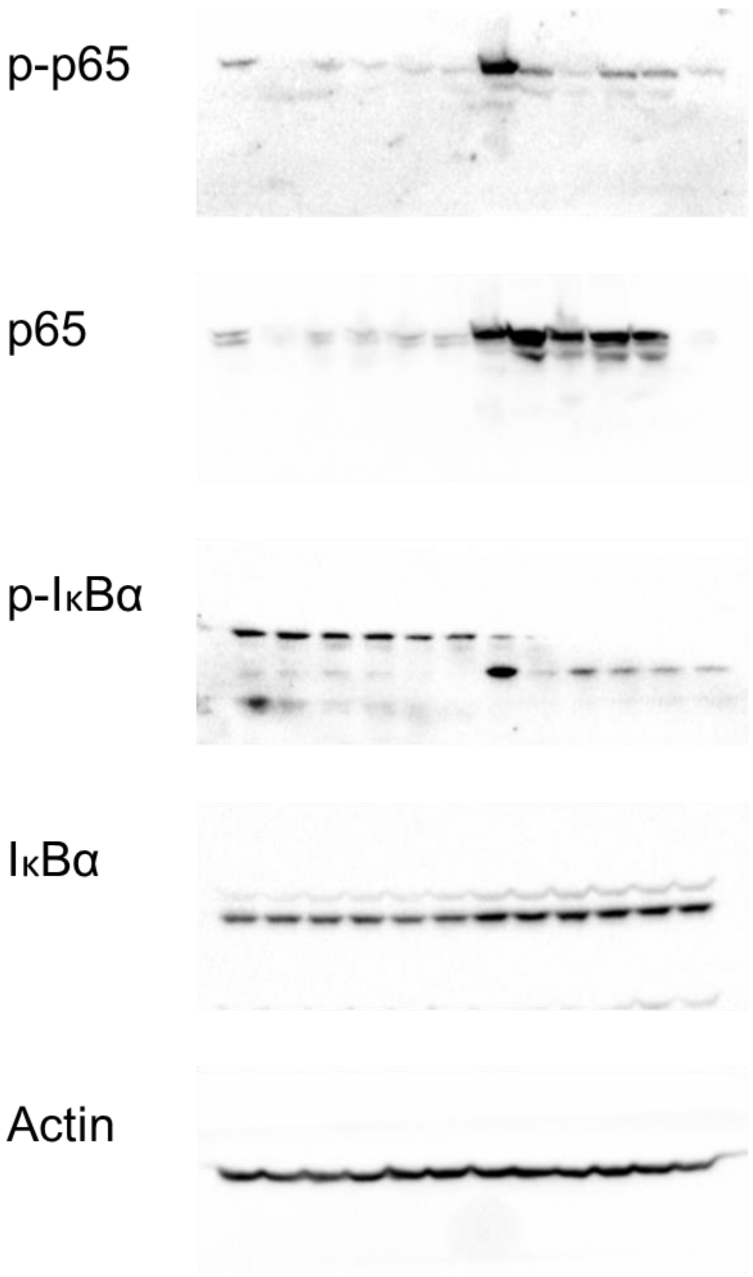

Figure S2. Original scan of Western blots.

Synthesis for Figure 1

General information for Chemistry

Unless stated, materials were obtained from commercial suppliers and used without further purification. ^{1}H and ^{13}C NMR spectra were recorded on a Bruker AV-400 spectrometer at 400 and 101 MHz, respectively. Coupling constants (*J*) are expressed in hertz (Hz). Chemical shifts of NMR are reported in parts per million (ppm) units relative to the solvent. The high resolution of MS (HRMS) was recorded on an Applied Biosystems Q-STAR Elite ESI-LC-MS/MS mass spectrometer. Melting points were measured using a YRT-3 melting point apparatus (Shanghai, China) and were uncorrected.

3,19-Diacetoxy-14α-(2'-methoxy-4'-nitro-phenoxy)-andrographolide (**4a**) and 3,19-Diacetoxy-14β-(2'-methoxy-4'-nitro-phenoxy)-andrographolide (**4b**)

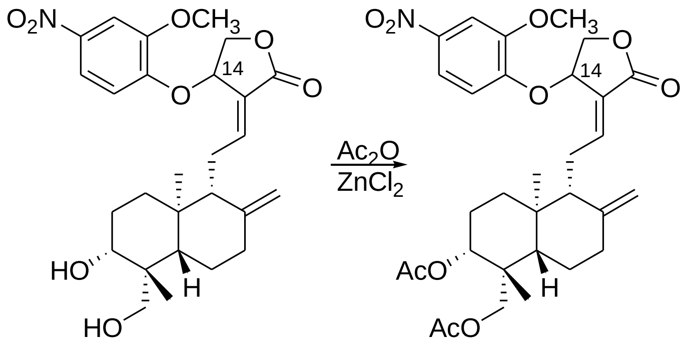

3a (14α) **4a (14α)** from **3a (14α)**
3b (14β) **4b (14β)** from **3b (14β)**

0.50 g of **3a** or **3b (**1.0 mmol）and 0.16 g of anhydrous $ZnCl_2$ (1.2mmol) were added into 10 ml of dichloromethane, and the mixture was then treated with 0.30 g of acetic anhydride (0.28 ml, 3.0 mmol) at 40 °C for 3h. After the reaction was complete, the reaction mixture was evaporated to dryness and the residue was dissolved by ethyl

acetate and washed with sat. $NaHCO_3$ solution and brine. The organic phase was dried over anhydrous Na_2SO_4, filtered, evaporated to dryness and the residue was purified by silica gel column chromatography (petroleum ether/ethyl acetate 5/3) to afford 0.54 g or 0.55 g of corresponding **4a** or **4b**.

4a (94.0 % yield), white solid, m.p.121.0 –123.0 °C. ^{1}H NMR (400 MHz, C_6D_6) δ 7.54 – 7.50 (m, 1H), 6.43 (m, 1H), 6.36-6.30 (m, 1H), 5.43 (s, 1H), 5.31 (d, J = 10.4 Hz, 1H), 5.12 (s, 1H), 4.67 (dd, J = 12.0, 4.4 Hz, 1H), 4.56 (d, J = 11.8 Hz, 1H), 4.09 (d, J = 11.8 Hz, 1H), 3.81 – 3.68 (m, 2H), 3.16 (s, 3H), 2.26 (m, 1H), 2.22 – 2.08 (m, 2H), 1.82 (m, 1H), 1.77 – 1.70 (m, 2H), 1.70 (s, 3H), 1.69 (s, 3H), 1.66 – 1.50 (m, 3H), 1.38 (m, 2H), 1.02 (dd, J = 12.6, 2.8 Hz, 1H), 0.88 (s, 3H), 0.64 (s, 3H). ^{13}C NMR (101 MHz, C6D6) δ 171.7, 169.9, 169.8, 152.9, 149.8, 146.9, 145.8, 142.5, 134.7, 117.7, 113.3, 109.1, 107.3, 79.8, 74.0, 70.1, 64.6, 55.5, 55.3, 52.0, 41.6, 39.1, 38.6, 37.0, 30.6, 25.0, 24.6, 22.5, 20.8, 20.6, 14.8. ESI-HRMS: *m/z* 608.2465 $[M+Na]^+$ calculated for $C_{33}H_{39}NNaO_{10}$, 608.2472.

4b (94.1% yield), white solid, m.p.76.6 - 77.2 °C. ^{1}H NMR (400 MHz, $CDCl_3$) δ 7.90 (dd, J = 8.8, 2.6 Hz, 1H), 7.84 (d, J = 2.5 Hz, 1H), 7.15 (t, J = 6.5 Hz, 1H), 6.84 (d, J = 8.8 Hz, 1H), 5.68 (d, J = 5.7 Hz, 1H), 4.89 (s, 1H), 4.60 (dd, J = 10.9, 5.9 Hz, 1H), 4.49 (dd, J = 11.7, 4.5 Hz, 1H), 4.45 – 4.38 (m, 2H), 4.32 (d, J = 11.8 Hz, 1H), 4.07 (d, J = 11.8 Hz, 1H), 3.98 (s, 3H), 2.51 – 2.38 (m, 2H), 2.35 – 2.25 (m, 1H), 2.04 (s, 1H), 2.03 (s, 3H), 2.02 (s, 3H), 1.98 – 1.83 (m, 3H), 1.60 (d, J = 12.9 Hz, 1H), 1.54 (s, 1H), 1.49 (dd, J = 13.0, 4.1 Hz, 1H), 1.31 (dd, J = 12.7, 2.3 Hz, 1H), 1.25 (m, 1H), 1.04 – 0.97 (m, 3H), 0.68 (s, 3H). ^{13}C NMR (101 MHz, C_6D_6) δ 169.8, 169.6, 168.1,

150.9, 150.3, 150.0, 147.4, 143.4, 125.2, 117.0, 114.2, 107.7, 107.5, 79.3, 72.6, 69.8, 64.3, 55.4^9, 55.4^6, 54.8, 41.4, 39.0, 37.8, 36.3, 27.1, 25.4, 24.5, 24.3, 22.5, 20.4^9, 20.4^5, 14.4. ESI-HRMS: *m/z* 608.2465 $[M+Na]^+$ calculated for $C_{33}H_{39}NNaO_{10}$, 608.2472.

14α-(2'-methoxy-4'-nitro-phenoxy)-19-acetoxy-andrographolide (**5a**) and 14β-(2'-methoxy-4'-nitro-phenoxy)-19-acetoxy-andrographolide (**5b**)

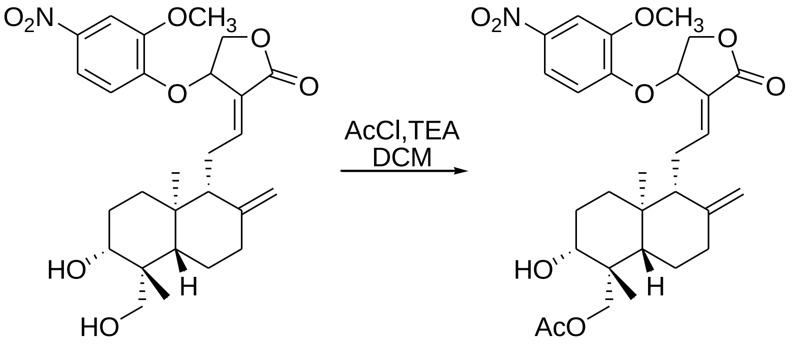

3a (14α)
3b (14β)

5a (14α) from **3a (14α)**
5b (14β) from **3b (14β)**

To the solution of 0.50 g of **3a** or **3b** (1.0 mmol) in 15 ml of dichloromethane, 0.15 g of trimethylamine (1.5 mmol) in 2 ml of dichloromethane was added and then treated with 0.094 g of acetyl chloride (1.2 mmol) in 2 ml of dichloromethane at 0 °C for 30min. After distilled off the volatile solvents, the residue was dissolved in ethyl acetate and washed with sat. $NaHCO_3$ solution and brine. The organic phase was dried over anhydrous Na_2SO_4, filtered, evaporated to dryness and residue was purified by silica gel column chromatography (petroleum ether/ethyl acetate 1/1) to afford **5a** or **5b**.

5a (27% yield), a yellow solid, m.p. 82.8 - 83.1 °C. ^1H NMR (400 MHz, DMSO-d6) δ 7.90 (dd, J = 8.9, 2.6 Hz, 1H), 7.80 (d, J = 2.6 Hz, 1H), 7.23 (d, J = 9.0 Hz, 1H), 6.92 (t, J = 6.3 Hz, 1H), 5.90 (d, J = 5.3 Hz, 1H), 4.82 (s, 1H), 4.71 (dd, J =

10.4, 5.8 Hz, 2H), 4.59 (s, 1H), 4.36 (d, *J* = 10.9 Hz, 1H), 4.09 (d, *J* = 11.7 Hz, 1H), 4.02 (d, *J* = 11.7 Hz, 1H), 3.87 (s, *J* = 17.7 Hz, 3H), 3.11 (dd, *J* = 12.6, 8.1 Hz, 1H), 2.41 – 2.29(m, 3H), 1.95 – 1.87 (m, 5H), 1.77 (d, *J* = 13.1 Hz, 1H), 1.53 – 1.37 (m, 4H), 1.22 – 1.15 (m, 3H), 0.99 (s, 3H), 0.55 (s, 3H). ^{13}C NMR (101 MHz, C_6D_6) δ 170.5, 168.4, 151.4, 150.9, 150.7, 147.0, 143.6, 124.9, 117.2, 115.5, 109.4, 107.6, 78.5, 73.6, 70.2, 64.9, 55.9, 55.5, 55.2, 42.7, 39.0, 38.0, 37.5, 28.2, 25.3, 24.6, 22.8, 20.6, 14.6. ESI-HRMS: *m*/*z* 566.2359 $[M + Na]^+$ calculated for $C_{29}H_{37}NO_9Na$ 566.2366.

5b (83% yield), a faint yellow solid, m.p. 115.1–116.0 °C. ^{1}H NMR (400 MHz, DMSO-d6) δ 7.94 (dd, *J* = 8.9, 2.7 Hz, 1H), 7.82 (d, *J* = 2.7 Hz, 1H), 7.25 (d, *J* = 9.0 Hz, 1H), 7.04 (t, *J* = 7.1 Hz, 1H), 6.00 (d, *J* = 5.0 Hz, 1H), 4.85 (s, 1H), 4.74 – 4.66 (m, 2H), 4.58 (s, 1H), 4.37 (d, *J* = 11.2 Hz, 1H), 4.08 (d, *J* = 11.7 Hz, 1H), 4.00 (d, *J* = 11.7 Hz, 1H), 3.92 (s, 3H), 2.94 – 2.85 (m, 1H), 2.43 – 2.28 (m, 3H), 1.96 (s, 3H), 1.91 (s, 2H), 1.79 (d, *J* = 12.9 Hz, 1H), 1.42 (dd, *J* = 21.4, 11.7 Hz, 3H), 1.27 (d, *J* = 10.1 Hz, 1H), 1.06 (d, *J* = 10.8 Hz, 1H), 0.99 (s, 3H), 0.90 (t, *J* = 13.0 Hz, 1H), 0.58 (s, 3H). ^{13}C NMR (101 MHz, DMSO-d6) δ 170.4, 168.9, 151.5, 150.6, 149.4, 147.6, 141.8 , 125.3, 117.4, 113.9, 107.8, 106.7, 76.6, 72.3, 70.7, 65.0, 56.1, 55.9, 54.0, 41.6, 38.9, 37.6, 36.2, 27.5, 24.9, 24.7, 22.8, 20.9, 13.8. ESI-HRMS: *m*/*z* 566.2360 $[M +Na]^+$ calculated for $C_{29}H_{37}NO_9Na$ 566.2366.

3-Oxo-14α-(2’-methoxy-4’-nitro-phenoxy)-19-acetoxy-andrographolide (**6a**) and 3-Oxo-14β-(2’-methoxy-4’-nitro-phenoxy)-19-acetoxy-andrographolide (**6b**)

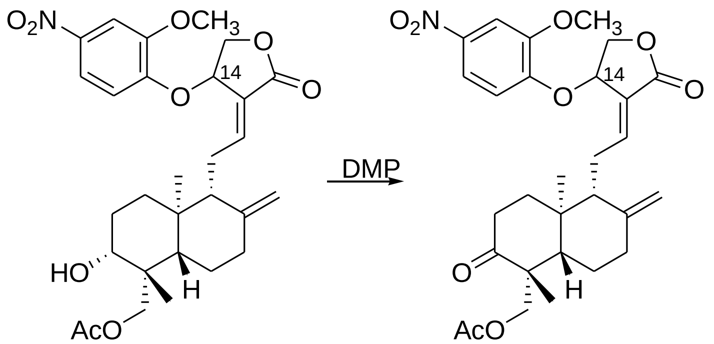

5a (14α)
5b (14β)

6a (14α) from **5a (14α)**
6b (14β) from **5b (14β)**

0.54 g of **5a** or **5b** (1.0mmol) was dissolved in 15 ml of dichloromethane and treated with 0.64 g of Dess-Martin periodinane (DMP, 1.5 mmol) in dark for 3 hours. The reaction was treated with 0.2 g of $Na_2S_2O_3$ in 10 ml of water for 20 min and the mixture was then extracted by ethyl acetate and washed with sat. $NaHCO_3$ solution and brine. After the organic phase was dried over anhydrous Na_2SO_4, filtered and evaporated to dryness,the residue was purified by silica gel column chromatography (petroleum ether/ethyl acetate 4/1) to afford about 90% yield of **6a** or **6b**.

6a, white solid, m. p. 81.4 - 82.7 ^{o}C. ^{1}H NMR (400 MHz, DMSO) δ 7.91 (d, J = 6.9 Hz, 1H), 7.81 (s, 1H), 7.24 (d, J = 8.9 Hz, 1H), 6.91 (s, 1H), 5.92 (s, 1H), 4.89 (s, 1H), 4.74– 4.68 (m, 2H), 4.46 (d, J = 11.1 Hz, 1H), 4.37 (d, J = 10.6 Hz, 1H), 3.88 (s, 3H), 2.74 – 2.71 (m, 1H), 2.47 – 2.43 (m, 2H), 2.36 (d, J = 11.6 Hz, 1H),2.14 – 2.03(m, 3H), 1.90 (s, 3H), 1.87 – 1.85(m, 1H), 1.71 (d, J = 9.4 Hz, 2H), 1.66 (s, 1H), 1.54–1.41 (m, 2H), 1.03 (s, 3H), 0.80 (s, 3H). ^{13}C NMR (101 MHz, C_6D_6) δ 210.3, 170.3, 168.3, 151.3, 150.6, 150.3, 146.3, 143.6, 125.1, 117.2, 115.4, 110.2, 107.6, 73.5, 70.1, 66.1, 56.7, 55.5, 55.0, 52.3, 38.8, 38.2, 37.6, 35.5, 25.3, 24.7, 20.8, 20.3, 14.7. ESI-HRMS: *m/z* 564.2204 $[M + Na]^+$ calculated for $C_{29}H_{35}NO_9Na$ 564.2210.

6b, white solid, m.p. 134.1 - 134.8 ^{o}C. ^{1}H NMR (400 MHz, C_6D_6) δ 7.58 (dd, J =

8.8, 2.6 Hz, 1H), 7.52 (d, J = 2.6 Hz, 1H), 7.11 (td, J = 7.1, 1.6 Hz, 1H), 5.92 (d, J = 8.8 Hz, 1H), 4.86 (d, J = 5.6 Hz, 1H), 4.80 (s, 1H), 4.65 (d, J = 11.3 Hz, 1H), 4.38 (s, 1H), 3.73 (dd, J = 10.8, 2.0 Hz, 1H), 3.69 – 3.61 (m, 2H), 3.11 (s, 3H), 2.59 (td, J = 14.5, 5.8 Hz, 1H), 2.14 – 2.08 (m, 2H), 2.08 – 2.05 (m, 2H), 1.62 (s, 3H), 1.60 – 1.52 (m, 1H), 1.43 (d, J = 5.8 Hz, 1H), 1.33 (m, 2H), 1.24 – 1.16 (m, 4H), 1.11 – 0.94 (m, 2H), 0.64 (s, 3H). ^{13}C NMR (101 MHz, C_6D_6) δ 210.1, 170.1, 168.0, 150.8, 150.2, 149.9, 147.0, 143.3, 125.0, 117.0, 114.3, 108.3, 107.3, 72.8, 69.7, 65.9, 56.4, 55.3, 54.7, 52.1, 38.9, 37.6, 37.3, 35.2, 25.6, 24.4, 20.6, 20.1, 14.3. ESI-HRMS: *m/z* 564.2208 $[M + Na]^+$ calculated for $C_{29}H_{35}NNaO_9$, 564.2210.

3,19-Acetonylidene-14β-(2’-methoxy-4’-nitro-phenoxy)-16-hydroxy-andrographolide (**7b**) and 3,19-Acetonylidene-14β-(2’-methoxy-4’-nitro-phenoxy)-15,16-dihydroxy-andrographolide (**8b**)

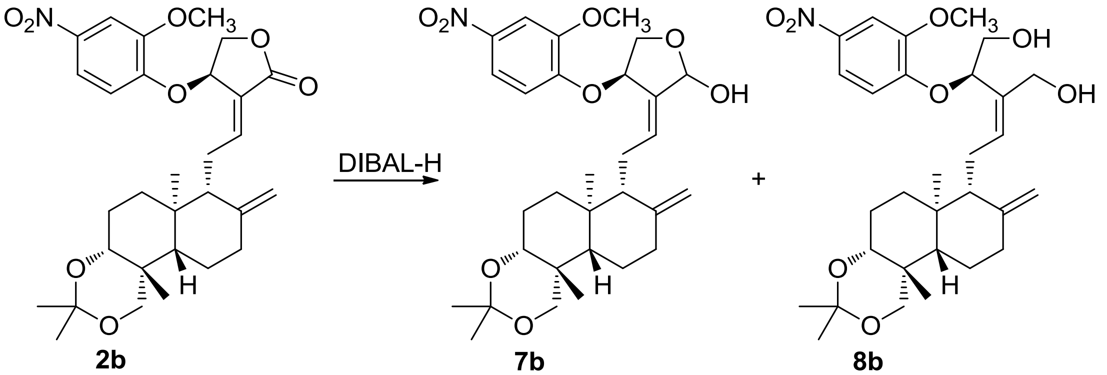

2b **7b** **8b**

Under N_2 atmosphere, 1.6 g of **2b** (1.0 mmol) was dissolved in 10 ml of anhydrous dichloromethane. After the solution was cooled to -78°C, 2.7ml of DIBAL-H (3.9mmol) in toluene was added dropwise and kept in the same temperature for 30 min. The reaction solution was treated with 5 ml of sat. Seignette

salt solution before the temperature rose to room temperature and washed with brine for about 3 times. After the solution was dried over anhydrous Na_2SO_4 and filtered, organic solution was evaporated to dryness and residue was purified by silica gel column chromatography by petroleum ether/ethyl acetate (3/1) to afford **7b** as a yellow solid 1.2 g (75% yield) and by petroleum ether/ethyl acetate (1/3) to afford **8b** as a light yellow solid 0.1 g (6% yield).

7b, m.p. 154.0-155.8 °C; ^{1}H NMR (400 MHz, DMSO-d_6) δ 9.35 (s, 1H), 7.86 (dd, J = 9.0, 2.7 Hz, 1H), 7.76 (d, J = 2.7 Hz, 1H), 6.80 (d, J = 9.2 Hz, 1H), 6.68 (t, J = 6.1 Hz, 1H), 5.42 (dd, J = 7.1, 3.6 Hz, 1H), 5.26 (t, J = 5.8 Hz, 1H), 4.53 (s, 1H), 3.90 – 3.80(m, 6H), 3.64 – 3.59 (m, 1H), 3.43 – 3.39 (m, 1H), 3.10 (d, J = 11.6 Hz, 1H), 2.76 – 2.74(m, 1H), 2.58 – 2.51 (m, 1H), 2.31 – 2.20 (m, 1H), 2.04 – 1.81 (m, 3H), 1.80 – 1.57 (m, 3H), 1.35 (s, 3H), 1.26 (s, 3H), 1.25 – 1.14 (m, 3H), 1.13 (s, 3H), 0.81 (s, 3H). ^{13}C NMR (101 MHz, DMSO-d_6) δ 194.0, 161.9, 152.4, 149.0, 147.6, 140.9, 137.0, 117.6, 112.0, 108.0, 106.6, 98.0, 76.3, 75.2, 62.7, 61.7, 56.1, 55.3, 51.9, 38.1, 37.0, 34.3, 27.9, 25.9, 25.4, 25.1, 24.4, 22.7, 15.3. ESI-HRMS: *m/z* 566.2723 $[M+Na]^+$ calculated for $C_{30}H_{41}NO_8Na$, 566.2730.

8b, m.p. 159.7-161.5 °C. ^{1}H NMR (400 MHz, DMSO-d_6) δ 7.87 (dd, J = 9.0, 2.7 Hz, 1H), 7.75 (d, J = 2.7 Hz, 1H), 7.11 (d, J = 9.1 Hz, 1H), 5.44 (t, J = 6.1 Hz, 1H), 5.14 – 5.08 (m, 2H), 4.85 (t, J = 5.3 Hz, 1H), 4.72 (s, 1H), 4.24 (s, 1H), 3.97 – 3.79 (m, 6H), 3.75 (dd, J = 13.6, 4.5 Hz, 1H), 3.65 – 3.60 (m, 1H), 3.39 (dd, J = 9.5, 3.8 Hz, 1H), 3.10 (d, J = 11.6 Hz, 1H), 2.44 (dd, J = 15.8, 6.8 Hz, 1H), 2.29 (d, J = 13.2 Hz, 1H), 2.06– 2.02(m, 1H), 1.97 – 1.82 (m, 2H), 1.78 – 1.54 (m, 4H), 1.34 (s, 3H),

1.25 (s, 3H), 1.21 (s, 2H), 1.15 (s, 1H), 1.12 (s, 3H), 0.81 (s, 3H). ^{13}C NMR (101 MHz, DMSO-d_6) δ 153.3, 149.1, 147.5, 140.7, 134.2, 130.2, 117.4, 113.1, 108.1, 106.6, 98.1, 79.7, 76.1, 62.7, 62.5, 62.1, 56.1, 56.0, 51.8, 38.2, 37.3, 37.1, 34.3, 27.7, 25.9, 25.4, 25.1, 22.8, 21.9, 15.5. ESI-HRMS: *m/z* 568.2884 $[M+Na]^+$ calculated for $C_{30}H_{43}NO_8Na$, 568.2886.

14β-(2'-methoxy-4'-nitro-phenoxy)-16-hydroxyl-andrographolide (**9b**)

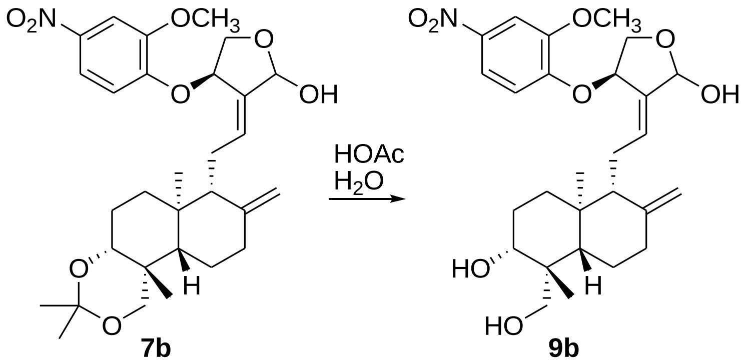

After 0.2 g of **7b** (0.3 mmol) was dissolved in 2 ml of acetic acid, 0.2 ml of H_2O was added and the reaction mixture was stirred at 10 ^{o}C for 1h. Treated with ethyl acetate and washed with sat. $NaHCO_3$ solution and brine. The organic phase was dried over anhydrous Na_2SO_4, filtered, evaporated to dryness and the residue was purified by silica gel column chromatography (dichloromethane/methanol 90/1) to afford **9b** as a light yellow solid 0.135 g (78% yield), m.p. 193.0-194.6 ^{o}C. ^{1}H NMR (400 MHz, CD_3OD) δ 9.33 (s, 1H), 7.83 (d, J = 7.1 Hz, 2H), 6.83 (d, J = 9.7 Hz, 1H), 6.69 (t, J = 6.2 Hz, 1H), 5.54 (dd, J = 6.9, 3.4 Hz, 1H), 4.54 (s, 1H), 4.11 (d, J = 11.1 Hz, 1H), 3.99 – 3.94 (m, 1H), 3.92 (s, 3H), 3.83 – 3.75 (m, 2H), 3.45 – 3.33 (m, 2H), 2.93 – 2.86 (m, 1H), 2.61 – 2.54 (m, 1H), 2.36 – 2.29 (m, 1H), 2.03 – 1.91 (m, 1H), 1.90 – 1.77 (m, 5H), 1.34 – 1.25(m, 3H), 1.20 (s, 3H), 0.70 (s, 3H). ^{13}C NMR (101 MHz,

CD_3OD) δ 195.1, 163.8, 153.7, 150.8, 148.9, 143.1, 138.6, 118.4, 113.2, 108.6, 107.8, 80.9, 76.6, 65.0, 63.7, 57.4, 56.8, 56.3, 43.7, 40.0, 38.9, 38.1, 29.0, 25.9, 25.2, 23.4, 15.5. ESI-HRMS: *m/z* 526.2407 $[M+Na]^+$ calculated for $C_{27}H_{37}NO_8Na$, 526.2417.

14β-(2'-methoxy-4'-nitro-phenoxy)-15,16-dihydroxy-andrographolide (**10b**)

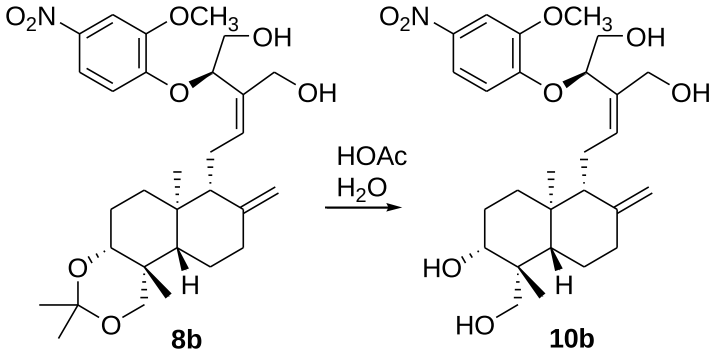

To the solution of 0.15 g of **8b** (0.15mmol) in 1 ml of acetic acid, 0.1 ml H_2O was added at 0 °C. After the reaction mixture was stirred at 10 °C for 1h, it was treated with ethyl acetate and then washed with sat $NaHCO_3$ solution and brine. The organic phase was dried over anhydrous Na_2SO_4, filtered, evaporated to dryness and the residue was purified by silica gel column chromatography (dichloromethane/methanol 70/1) to afford **10b** (60% yield) as a light yellow solid, m. p. 139.2~141.4 °C. ^1H NMR (500 MHz, DMSO-d_6) δ 7.86 (dd, *J* = 8.9, 2.5 Hz, 1H), 7.75 (d, *J* = 2.5 Hz, 1H), 7.10 (d, *J* = 9.0 Hz, 1H), 5.44 (t, *J* = 5.9 Hz, 1H), 5.12 (dd, *J* = 7.6, 3.3 Hz, 1H), 5.04 (d, *J* = 4.7 Hz, 2H), 4.80 (t, *J* = 4.6 Hz, 1H), 4.69 (s, 1H), 4.22 (s, 1H), 4.11 (d, *J* = 6.4 Hz, 1H), 3.96 – 3.87 (m, 4H), 3.84 (d, *J* = 10.7 Hz, 2H), 3.75 (dd, *J* = 13.3, 4.4 Hz, 1H), 3.70 – 3.59 (m, 1H), 3.26 – 3.24 (m, 2H), 2.45 (dd, *J* = 16.1, 6.8 Hz, 1H), 2.28 (d, *J* = 12.5 Hz, 1H), 1.99 – 1.97 (m,1H), 1.92 – 1.76 (m, 2H), 1.75 – 1.62 (m, 3H), 1.57 (d, *J* = 10.8 Hz, 1H), 1.32 – 1.28 (m,1H), 1.16 – 1.10 (m, 2H), 1.07 (s, 3H), 0.61 (s,

3H). ^{13}C NMR (101 MHz, DMSO-d_6) δ 153.3, 149.1, 147.6, 140.7, 134.1, 130.2, 117.4, 112.9, 107.6, 106.6, 79.7, 78.5, 62.7, 62.6, 62.2, 56.3, 56.0, 54.5, 42.3, 38.7, 37.7, 36.6, 28.0, 24.0, 23.1, 21.7, 14.7. ESI-HRMS: *m/z* 528.2569 $[M+Na]^+$ calculated for $C_{27}H_{39}NO_8Na$, 528.2573.

Synthesis of Figure S1-1

3,19-Bis(tert-butyldimethylsilyloxy)-14β-(2’-methoxy-4’-nitro-phenoxy)-16α-methoxy-andrographolide (**12ba**), 3,19-bis(tert-butyldimethylsilyloxy)-14β-(2’-methoxy-4’-nitro-phenoxy)-16β-methoxy-andrographolide (**12bb**), 14β-(2’-methoxy-4’-nitro-phenoxy)-16α-methoxy-andrographolide (**11ba**), and 14β-(2’-methoxy-4’-nitro-phenoxy)-16β-methoxy-andrographolide (**11bb**)

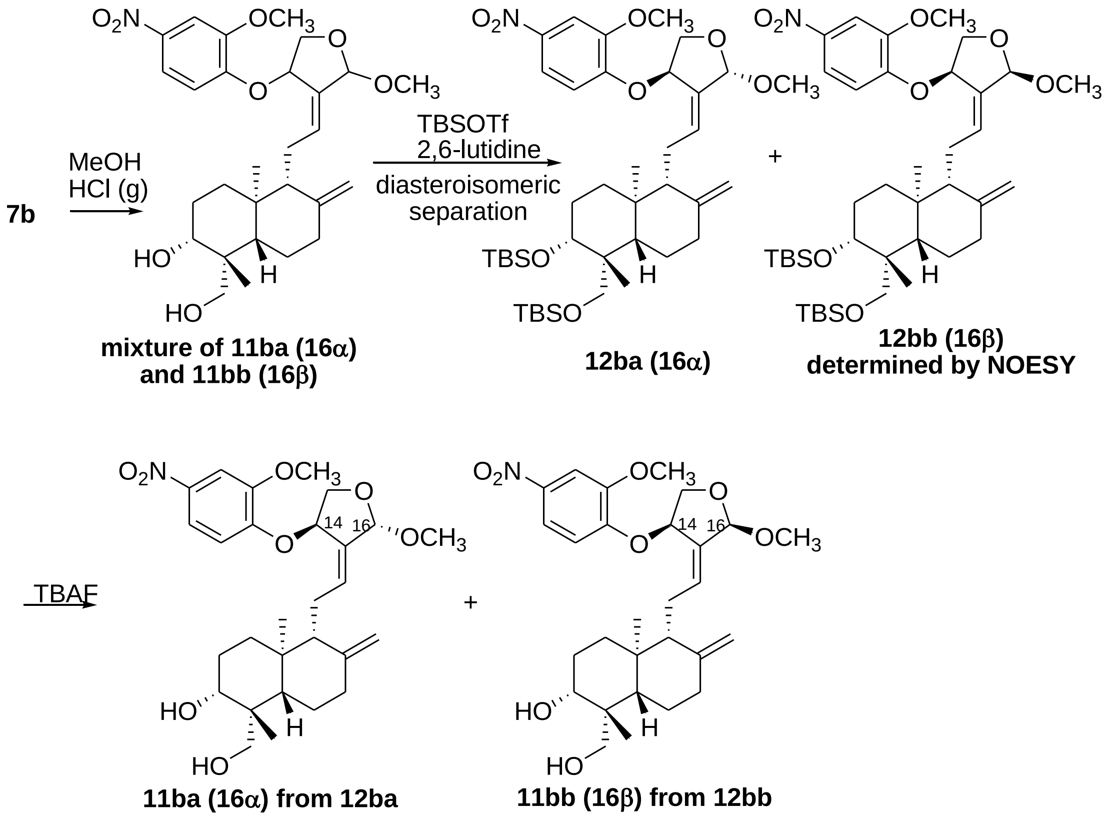

Figure S1-1. Preparation and seperation of diasteroisomers **11ba** and **11bb** from **7b**.

To the solution of 0.2 g of **7b** (0.3 mmol) in 2.5 ml of methanol at 0 °C, 2.5 ml of methanol saturated with HCl(g) was added dropwise at 0°C and the reaction was stirred at 0 °C for 1 hour before treated with ethyl acetate and carefully washed with sat. $NaHCO_3$ solution and brine. The organic phase was dried over anhydrous Na_2SO_4, filtered, evaporated to dryness and residue was purified by silica gel column

chromatography (dichloromethane/methanol 90/1) to afford the mixture of **11ba** and **11bb** as light yellow solid 0.134 g (86% yield).

The mixture of **11ba** and **11bb** was dissolved in 3 ml of dry dichloromethane and the solution was cooled to 0 ^{o}C before 0.13 ml of 2,6-lutidine (1.3 mmol) and then 0.23 ml of TBSOTf (1.1 mmol) were added. After the reaction was complete in about 1 hour, it was treated with sat. $NaHCO_3$ solution and extracted with ethyl acetate and washed with sat. $NaHCO_3$ solution and brine. The organic phase was dried over anhydrous Na_2SO_4, filtered and evaporated in reduced pressure to dryness and the residue was silica gel chromatographed to give 53% yield of **12ba** and 13% yield of **12bb**.

12ba, pale yellow solid, m.p. 167.3-169.8 ^{o}C. ^{1}H NMR (400 MHz, C_6D_6) δ 7.66 (dd, J = 8.8, 2.6 Hz, 1H), 7.60 (d, J = 2.6 Hz, 1H), 6.28 (t, J = 7.4 Hz, 1H), 6.06 (d, J = 8.9 Hz, 1H), 5.30 (s, 1H), 5.02 (d, J = 3.0 Hz, 1H), 4.94 (s, 1H), 4.63 (s, 1H), 4.02 (d, J = 10.5 Hz, 1H), 3.98 (d, J = 3.6 Hz, 1H), 3.91 (d, J = 10.5 Hz, 1H), 3.79 (d, J = 10.5 Hz, 1H), 3.23 (s, 3H), 3.19 (s, 3H), 3.00 (dd, J = 11.9, 4.4 Hz, 1H), 2.42 (dd, J = 9.4, 2.4 Hz, 1H), 2.26 – 2.24 (m, 1H), 2.14 – 2.13(m, 1H), 2.04 – 1.82 (m, 4H), 1.57 – 1.55 (m, 1H), 1.40 – 1.38 (m, 1H), 1.30 – 1.19 (m, 1H), 1.11 (s, 3H), 0.98 (d, J = 1.2 Hz, 19H), 0.87 (s, 3H), 0.80 – 0.76 (m, 1H), 0.13 (s, 3H), 0.09 (s, 3H), 0.06 (s, 3H), 0.03 (s, 3H). ^{13}C NMR (101 MHz, C_6D_6) δ 152.6, 150.2, 148.8, 142.4, 138.5, 135.3, 117.5, 112.6, 107.4, 107.0, 105.2, 79.8, 76.5, 70.7, 64.8, 57.6, 55.5, 55.3, 54.9, 44.1, 39.7, 39.3, 37.2, 28.7, 26.9, 26.2, 26.1, 25.3, 24.1, 18.5, 18.3, 14.5, -3.8, -4.9, -5.4, -5.5. ESI-HRMS: *m/z* 768.4294 $[M+Na]^+$ calculated for $C_{40}H_{67}NO_8Si_2Na$, 768.4303.

12bb, pale yellow solid, m.p. 134.2-135.7 °C. ^{1}H NMR (400 MHz, C_6D_6) δ 7.65 (dd, J = 8.8, 2.6 Hz, 1H), 7.57 (d, J = 2.6 Hz, 1H), 6.22 (d, J = 8.9 Hz, 1H), 6.00 (t, J = 6.8 Hz, 1H), 5.22 (s, 1H), 5.01 (t, J = 4.7 Hz, 1H), 4.92 (s, 1H), 4.56 (s, 1H), 4.11 – 3.99 (m, 2H), 3.96 (dd, J = 9.7, 4.5 Hz, 1H), 3.80 (d, J = 10.5 Hz, 1H), 3.28 (s, 3H), 3.18 (s, 3H), 3.11 (dd, J = 11.9, 4.2 Hz, 1H), 2.46 – 2.34(m, 1H), 2.24 (dd, J = 12.4, 7.1 Hz, 1H), 2.15 – 2.03 (m, 1H), 2.02 – 1.74 (m, 4H), 1.69 – 1.50 (m, 1H), 1.41 – 1.29 (m, 2H), 1.13 (s, 3H), 1.00 (s, 9H), 0.97 (s, 10H), 0.86 (s, 3H), 0.84 – 0.74 (m, 1H), 0.14 (s, 3H), 0.08 (d, J = 4.8 Hz, 6H), 0.05 (s, 3H). ^{13}C NMR (101 MHz, C_6D_6) δ 152.9, 150.3, 149.0, 142.7, 137.0, 134.3, 117.5, 113.8, 107.2, 107.2, 105.3, 79.8, 75.5, 71.5, 64.8, 57.1, 55.5, 55.4, 54.1, 44.1, 39.5, 39.2, 37.2, 28.7, 26.8, 26.2, 26.1, 24.4, 24.0, 18.5, 18.3, 14.6, -3.7, -4.8, -5.4, -5.5. ESI-HRMS: *m/z* 768.4299 $[M+Na]^+$ calculated for $C_{40}H_{67}NO_8Si_2Na$, 768.4303. The absolute structure of **12bb** was determined by NOESY (in this SI at pp 3-4).

About 1.0 mmol of **12ba** or **12bb** was dissolved in 10 ml of dry THF and 3.9 g of TBAF (15.0 mmol) was added at 0 °C. The reaction was heated at 50 °C for 18 hours until **12ba** or **12bb** was disappeared and then the complete reaction was treated with ethyl acetate and sat. $NaHCO_3$ solution and washed with brine. After the organic phase was dried over anhydrous Na_2SO_4, filtered, evaporated and dried in vacuum. The residue was purified by silica gel column chromatography (dichloromethane/methanol 160/1) to afford **11ba** or **11bb** as a light yellow solid in 95% yield.

11ba, m.p. 169.8~172.6 °C. ^{1}H NMR (400 MHz, DMSO-d_6) δ 7.90 (dd, J = 8.9,

2.6 Hz, 1H), 7.78 (d, *J* = 2.6 Hz, 1H), 7.22 (d, *J* = 9.1 Hz, 1H), 5.99 (dd, *J* = 7.8, 7.0 Hz, 1H), 5.71 – 5.62 (m, 1H), 5.35 (s, 1H), 4.97 (d, *J* = 4.8 Hz, 1H), 4.81 (s, 1H), 4.54 (s, 1H), 4.16 – 4.03 (m, 2H), 3.91 (d, *J* = 13.0 Hz, 4H), 3.73 (dd, *J* = 11.0, 2.8 Hz, 1H), 3.26 (s, 3H), 3.17 (dd, *J* = 10.8, 7.9 Hz, 1H), 2.95 – 2.85 (m, 1H), 2.39 – 2.18 (m, 2H), 2.10 – 1.97 (m, 1H), 1.95 – 1.81 (m, 1H), 1.81 – 1.63 (m, 2H), 1.49 – 1.19 (m, 4H), 1.03 (s, 3H), 1.01 – 0.94 (m, 1H), 0.77 (td, *J* = 13.9, 13.3, 2.6 Hz, 1H), 0.51 (s, 3H). ^{13}C NMR (101 MHz, DMSO-d_6) δ 152.5, 149.2, 147.6, 141.0, 137.6, 134.4, 117.4, 112.8, 107.4, 106.3, 103.8, 78.5, 75.7, 70.5, 62.5, 56.1, 55.9, 54.4, 54.2, 42.1, 38.6, 37.6, 35.9, 27.8, 24.2, 24.0, 23.1, 14.6. ESI-HRMS: *m/z* 540.2567 [M+Na]$^+$ calculated for $C_{28}H_{39}NO_8Na$, 540.2573.

11bb, m.p. 178.3~180.7 ^{o}C. ^{1}H NMR (400 MHz, DMSO-d_6) δ 7.88 (dd, *J* = 8.9, 2.7 Hz, 1H), 7.77 (d, *J* = 2.7 Hz, 1H), 7.21 (d, *J* = 9.1 Hz, 1H), 5.95 (t, *J* = 7.1 Hz, 1H), 5.57 (t, *J* = 4.4 Hz, 1H), 5.20 (s, 1H), 4.99 (d, *J* = 4.8 Hz, 1H), 4.80 (s, 1H), 4.55 – 4.39 (m, 2H), 4.06 (dd, *J* = 7.6, 2.8 Hz, 1H), 3.90 (s, 3H), 3.78 – 3.72 (m, 2H), 3.26 (s, 3H), 3.17 (dd, *J* = 10.8, 7.8 Hz, 1H), 3.01 – 2.89 (m, 1H), 2.38 – 2.13 (m, 2H), 2.00 – 1.88 (m, 2H), 1.73 (dd, *J* = 24.4, 11.9 Hz, 2H), 1.53 – 1.19 (m, 4H), 1.04 – 1.01 (m, 4H), 0.84– 0.83 (m, 1H), 0.50 (s, 3H). ^{13}C NMR (101 MHz, DMSO-d_6) δ 152.8, 149.0, 147.8, 141.1, 136.5, 133.4, 117.5, 112.9, 107.3, 106.4, 104.1, 78.5, 74.4, 70.8, 62.6, 56.0, 54.9, 54.4, 53.7, 42.2, 38.7, 37.6, 35.9, 27.9, 24.0, 23.7, 23.1, 14.6. ESI-HRMS: *m/z* 540.2573 [M+Na]$^+$ calculated for $C_{28}H_{39}NO_8Na$, 540.2573.

^{1}H NMR of **4a**

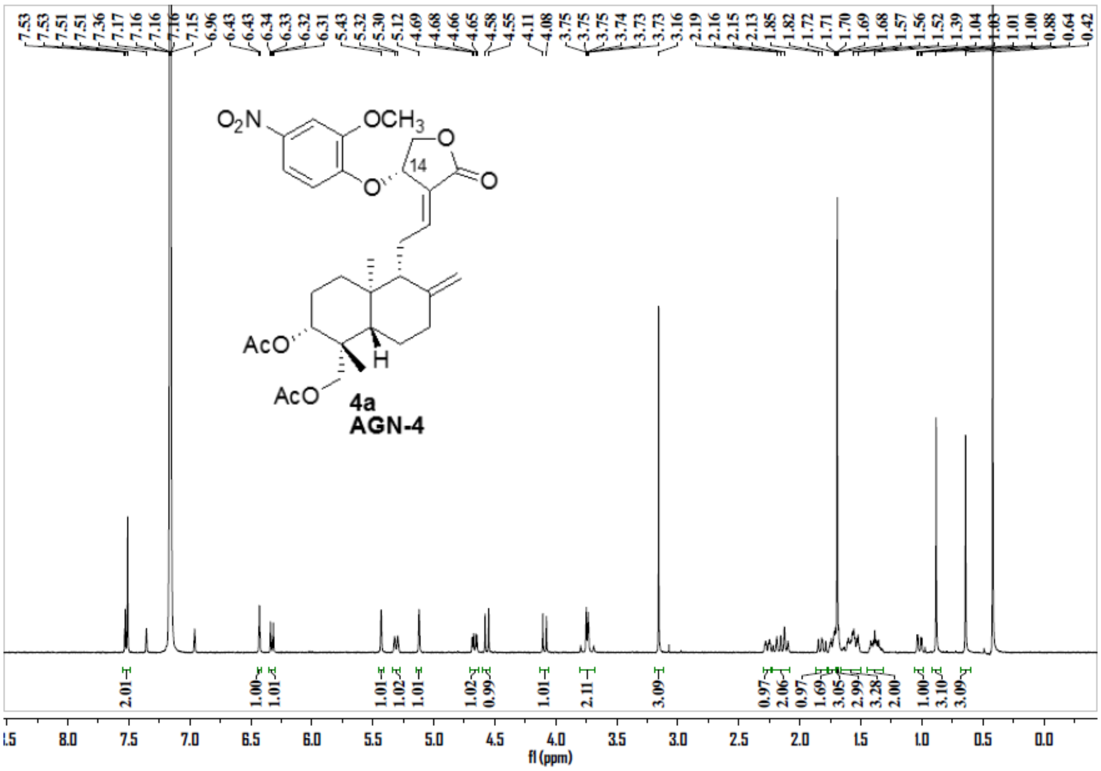

^{13}C NMR of **4a**

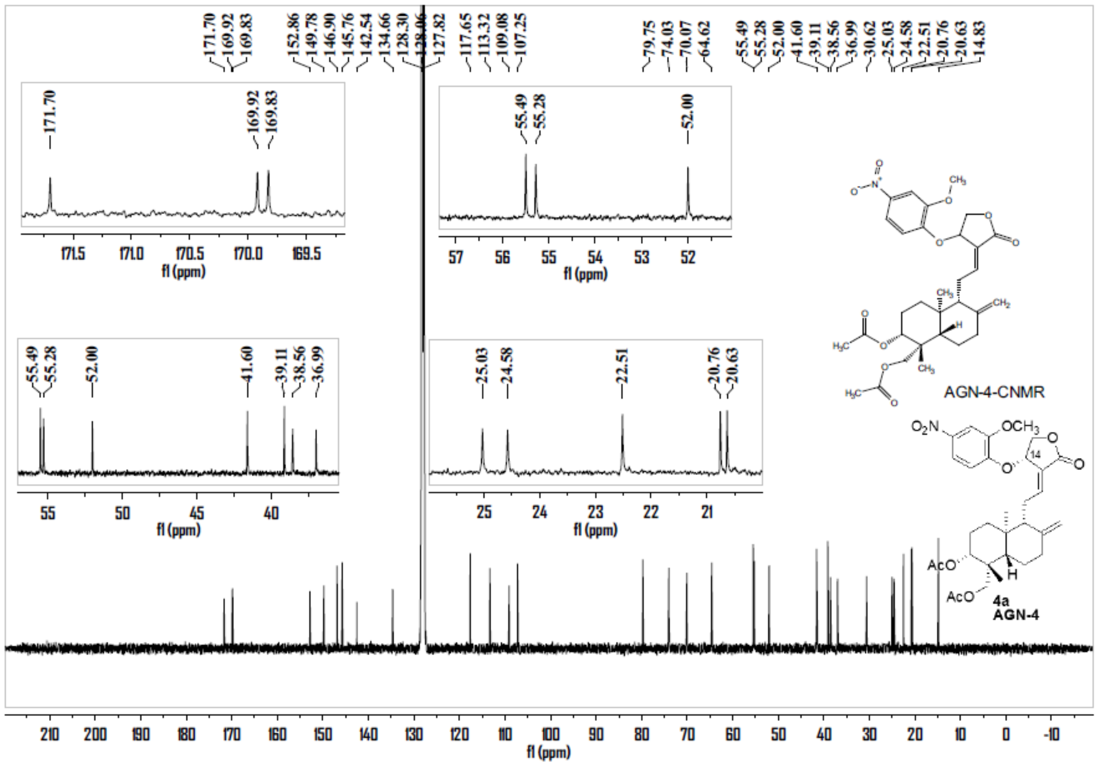

^{1}H NMR of **4b**

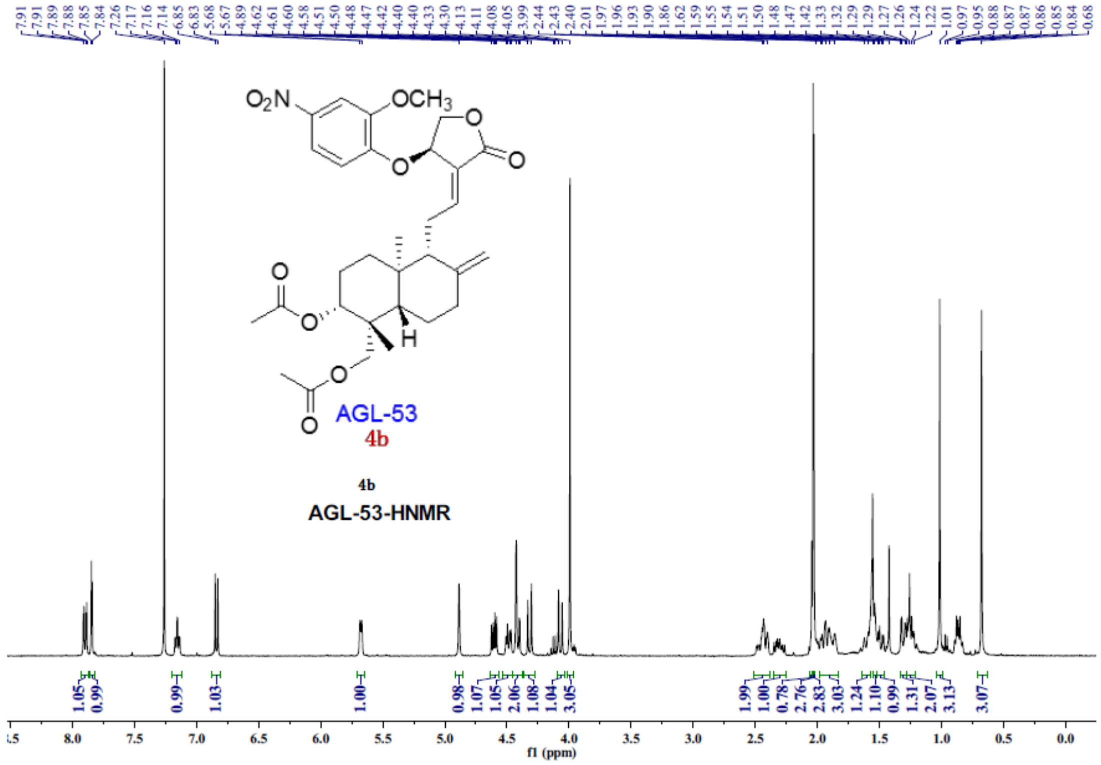

^{13}C NMR of **4b**

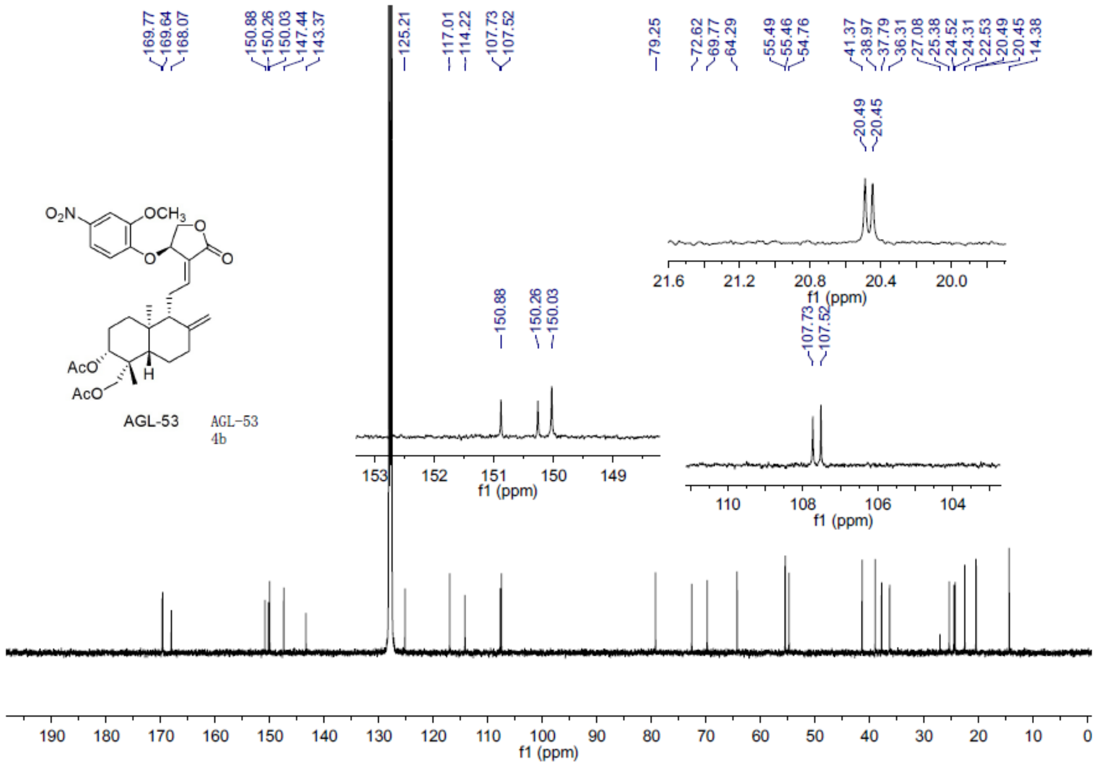

^{1}H NMR of **5a**

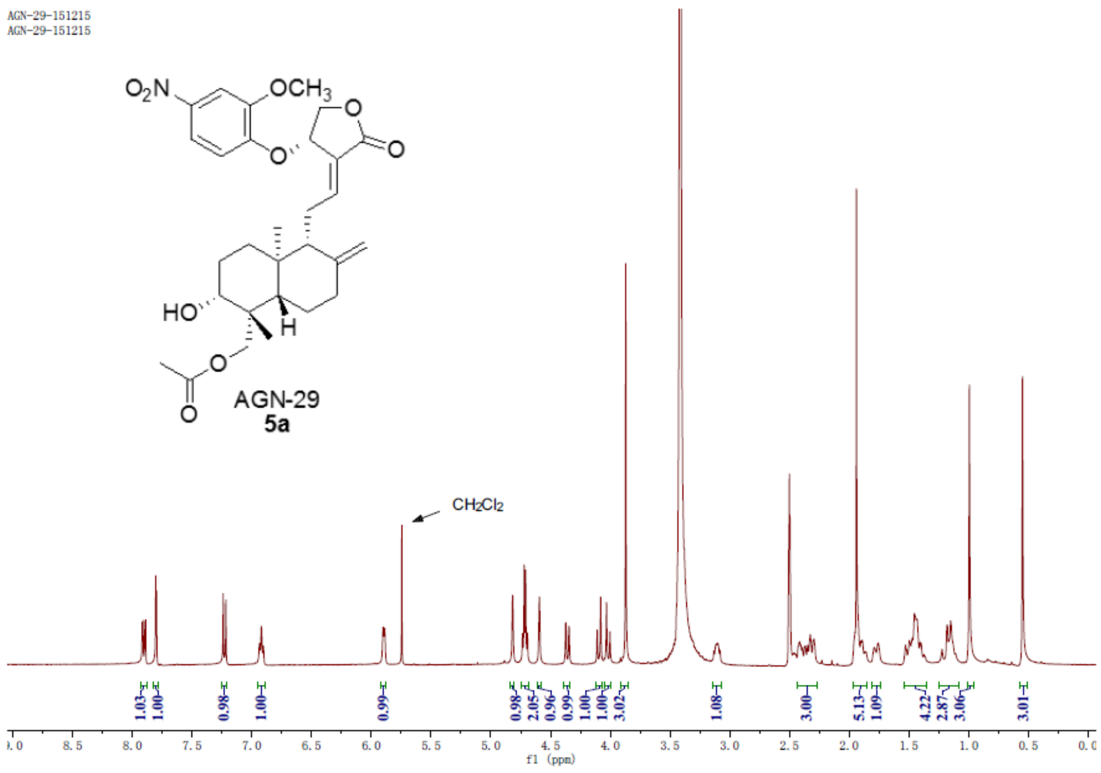
AGN-29-151215
AGN-29-151215
AGN-29
5a
CH2Cl2
f1 (ppm)

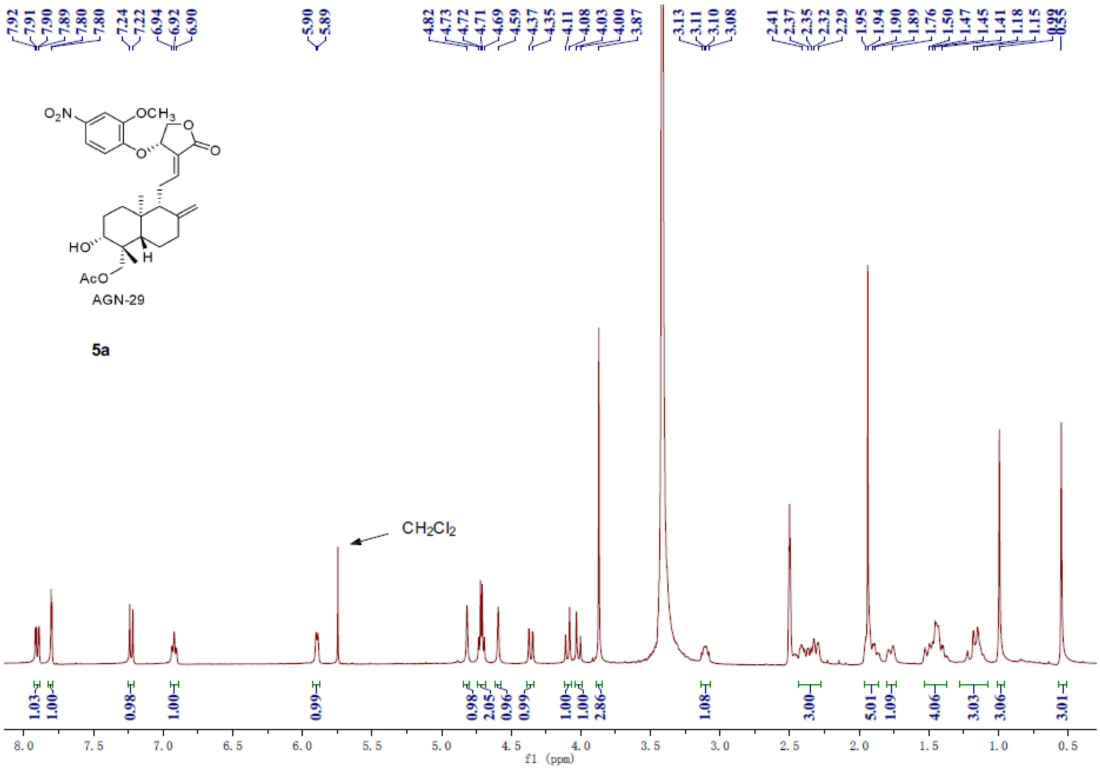
AGN-29
5a
CH2Cl2
f1 (ppm)

^{13}C NMR of **5a**

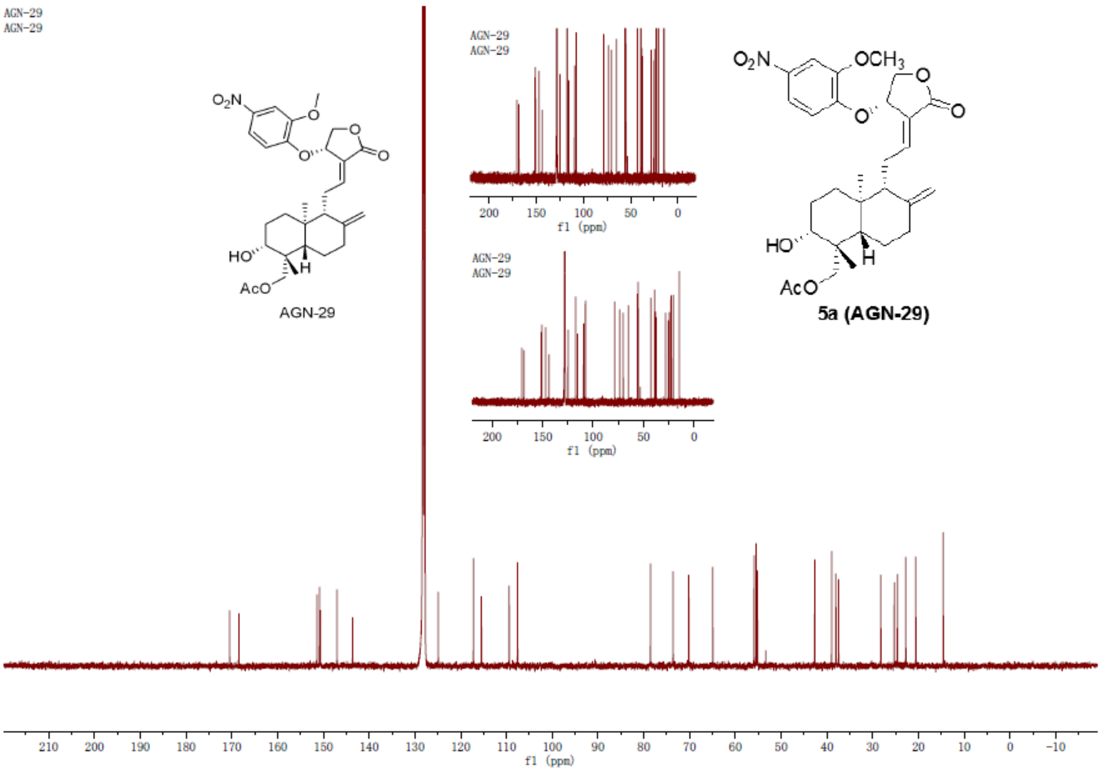
AGN-29
AGN-29
AGN-29
5a (AGN-29)
f1 (ppm)

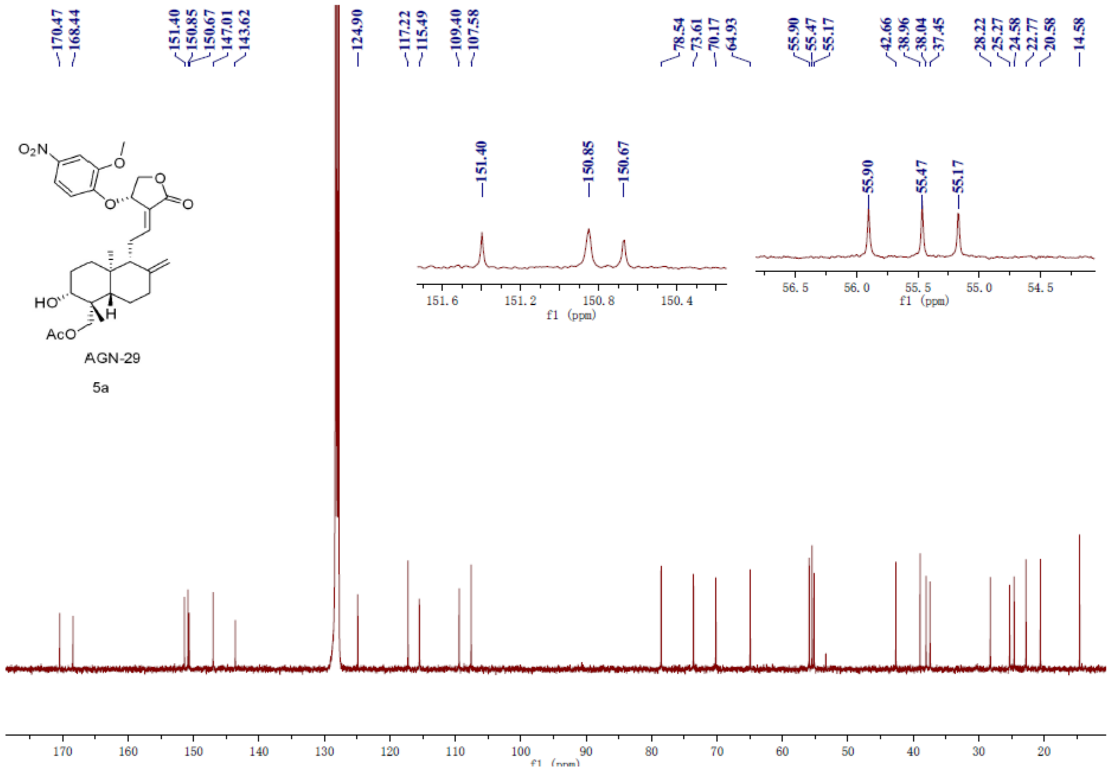
170.47
168.44
151.40
150.85
150.67
147.01
143.62
124.90
117.22
115.49
109.40
107.58
78.54
73.61
70.17
64.93
55.90
55.47
55.17
42.66
38.96
38.04
37.45
28.22
25.27
24.58
22.77
20.58
14.58
AGN-29
5a
f1 (ppm)

^{1}H NMR of **5b**

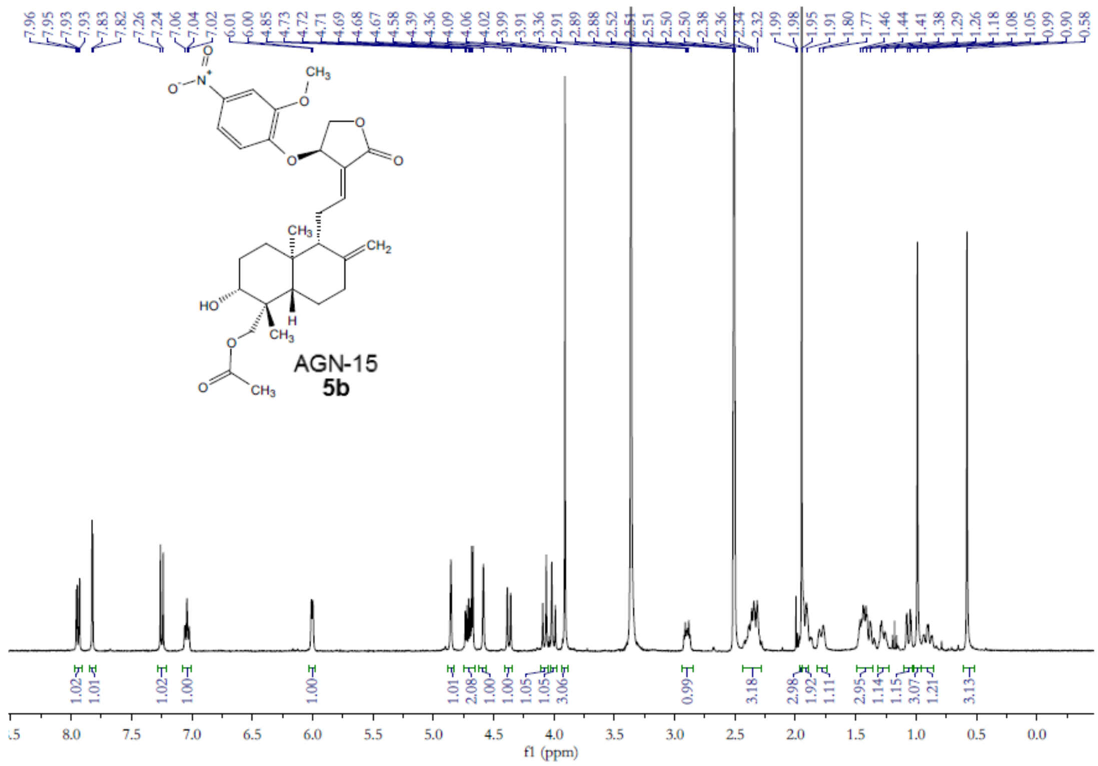

^{13}C NMR of **5b**

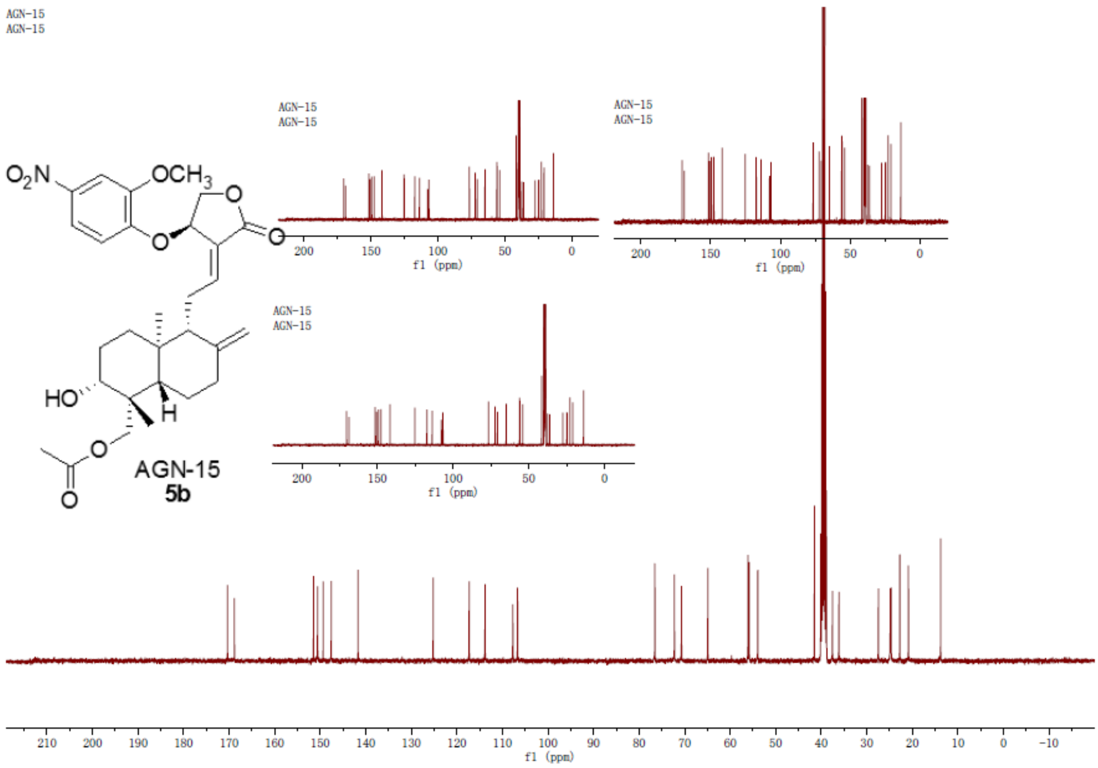

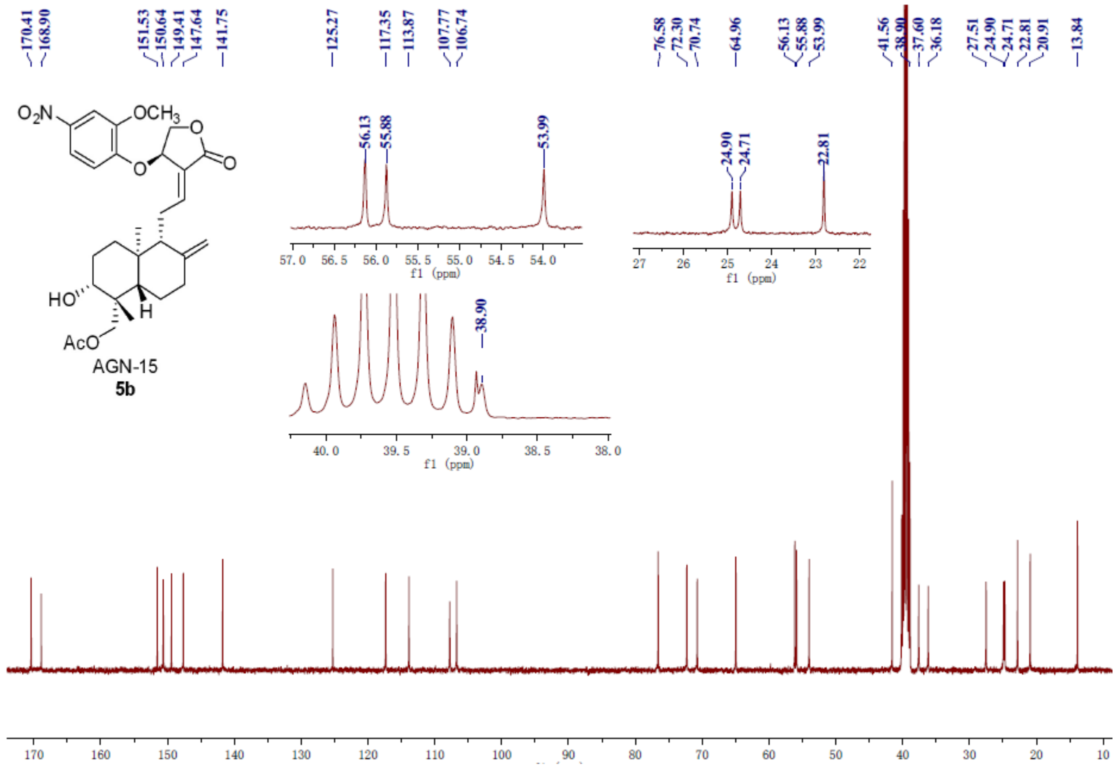

^{1}H NMR of **6a**

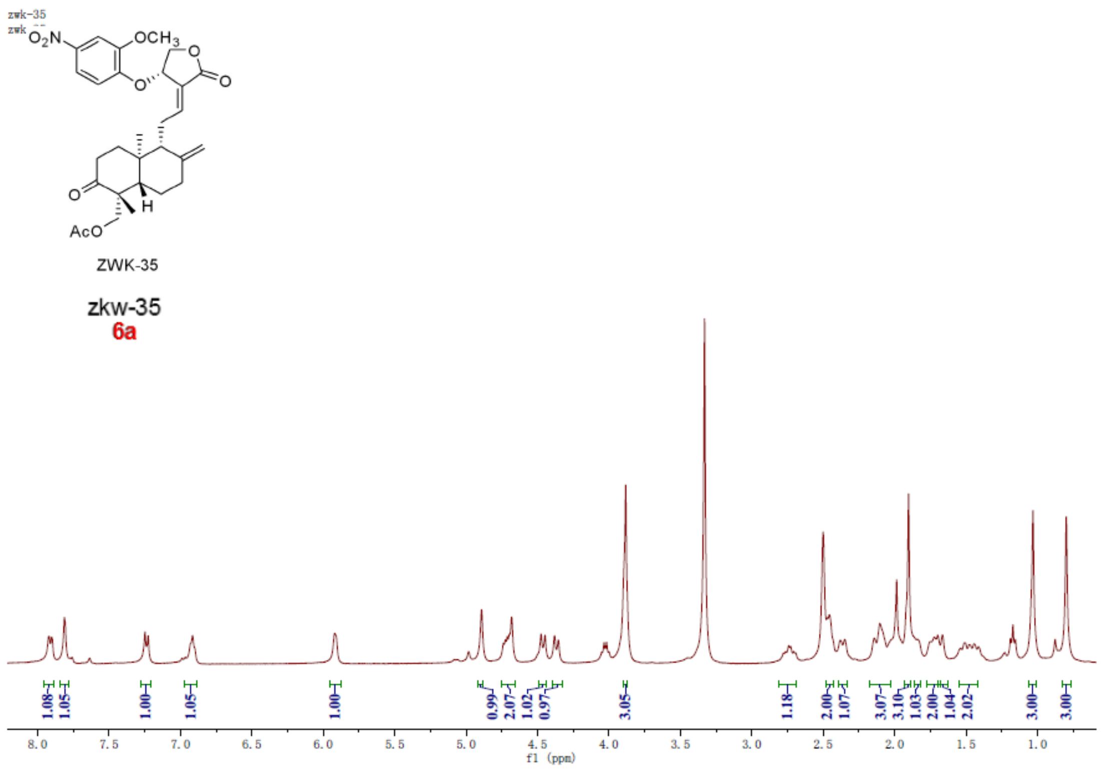

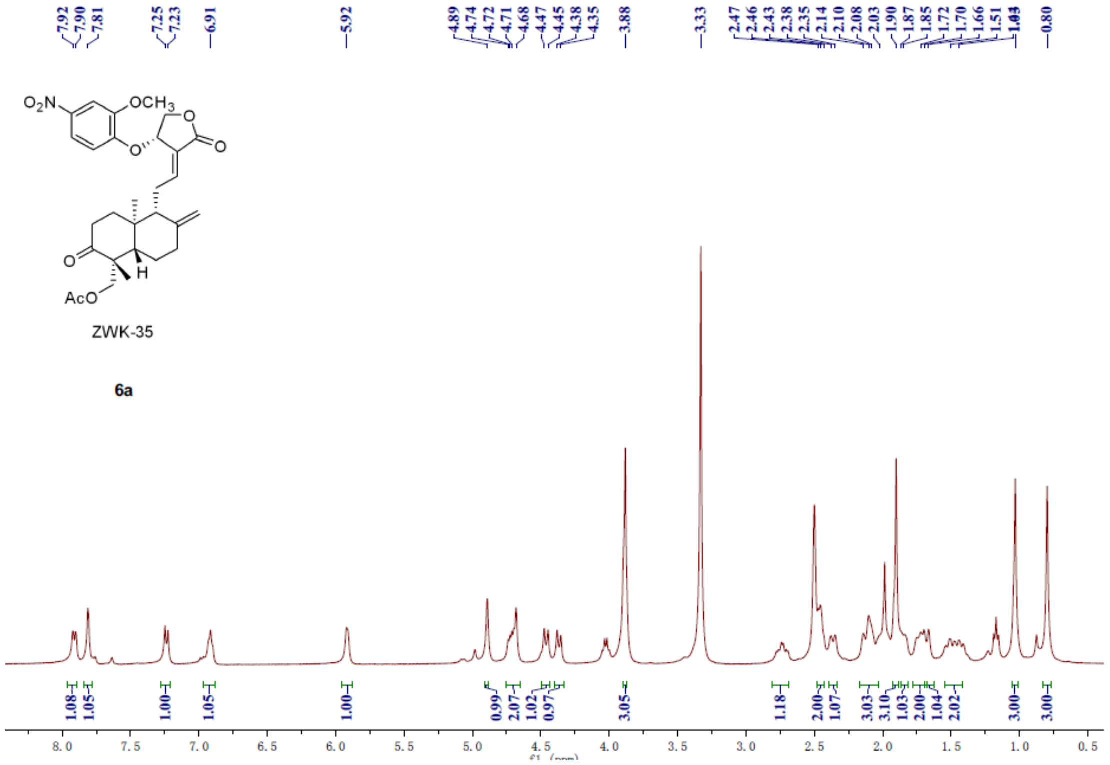

^{13}C NMR of **6a**

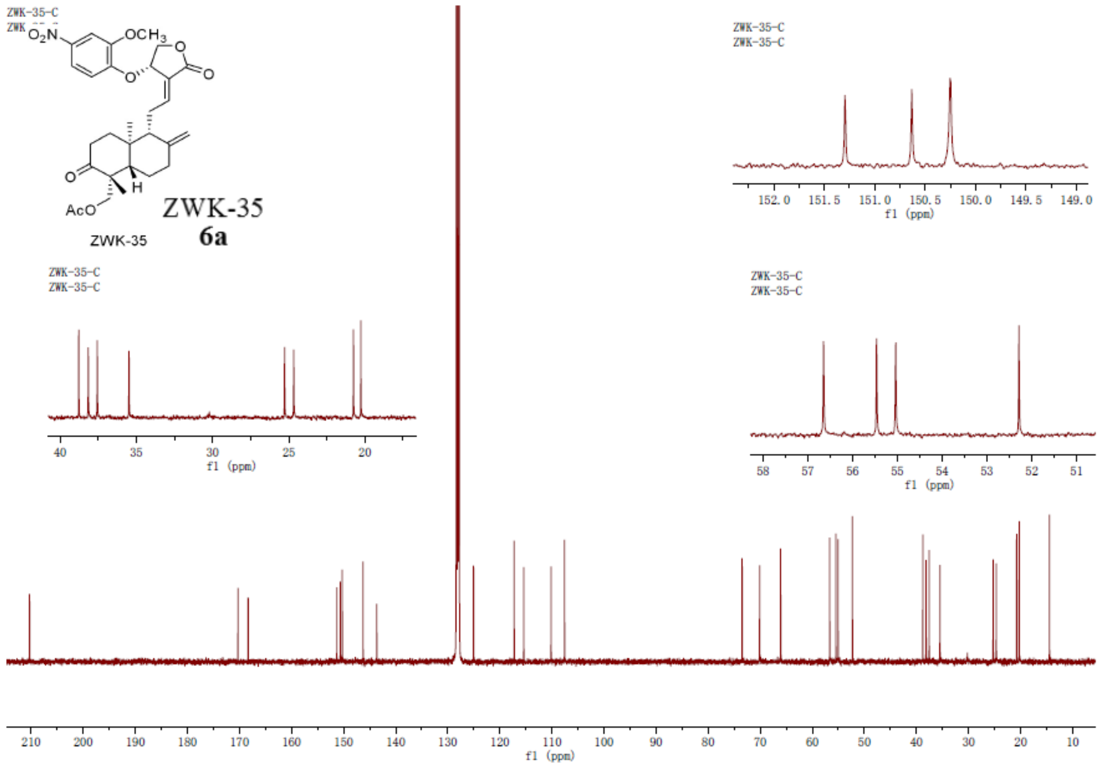

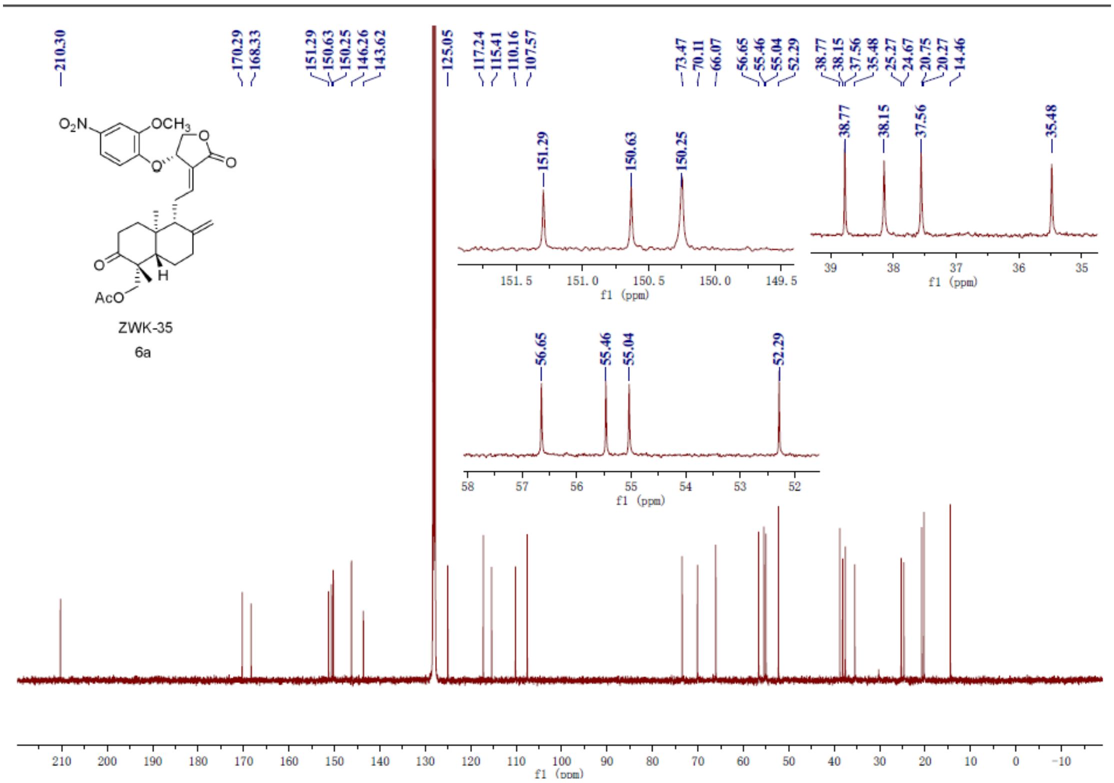

^{1}H NMR of **6b**

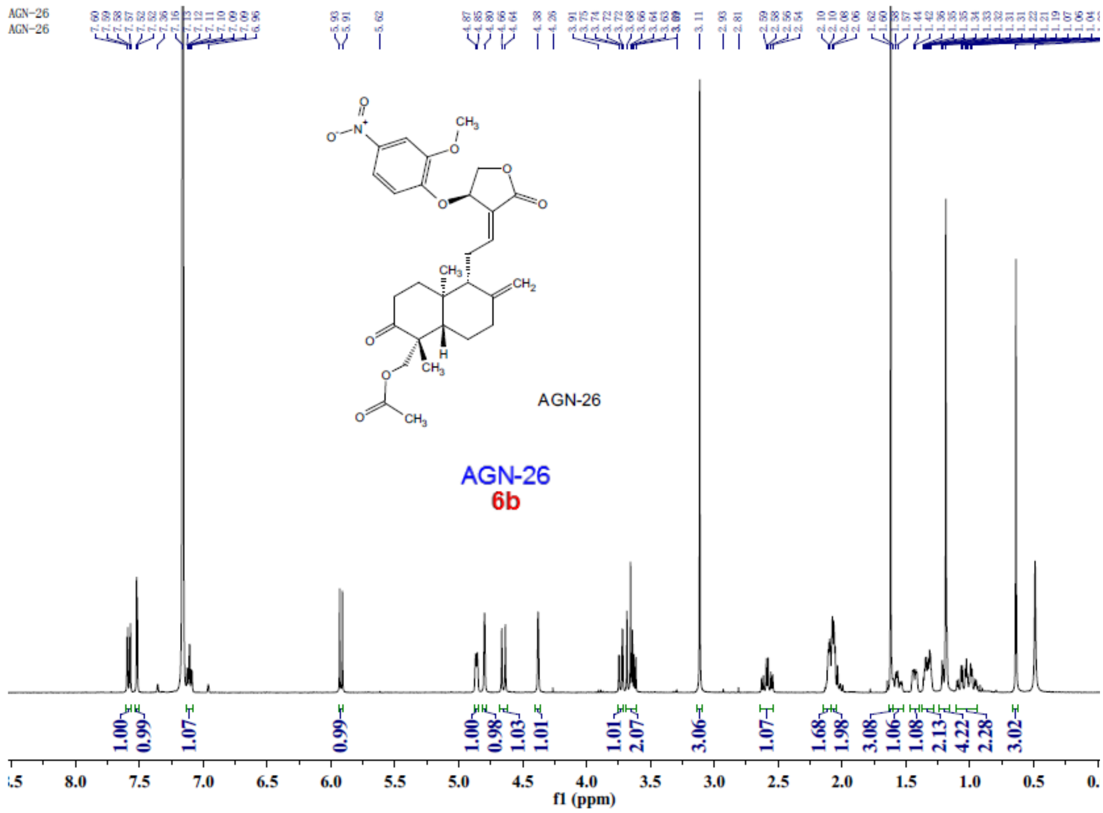

^{13}C NMR of **6b**

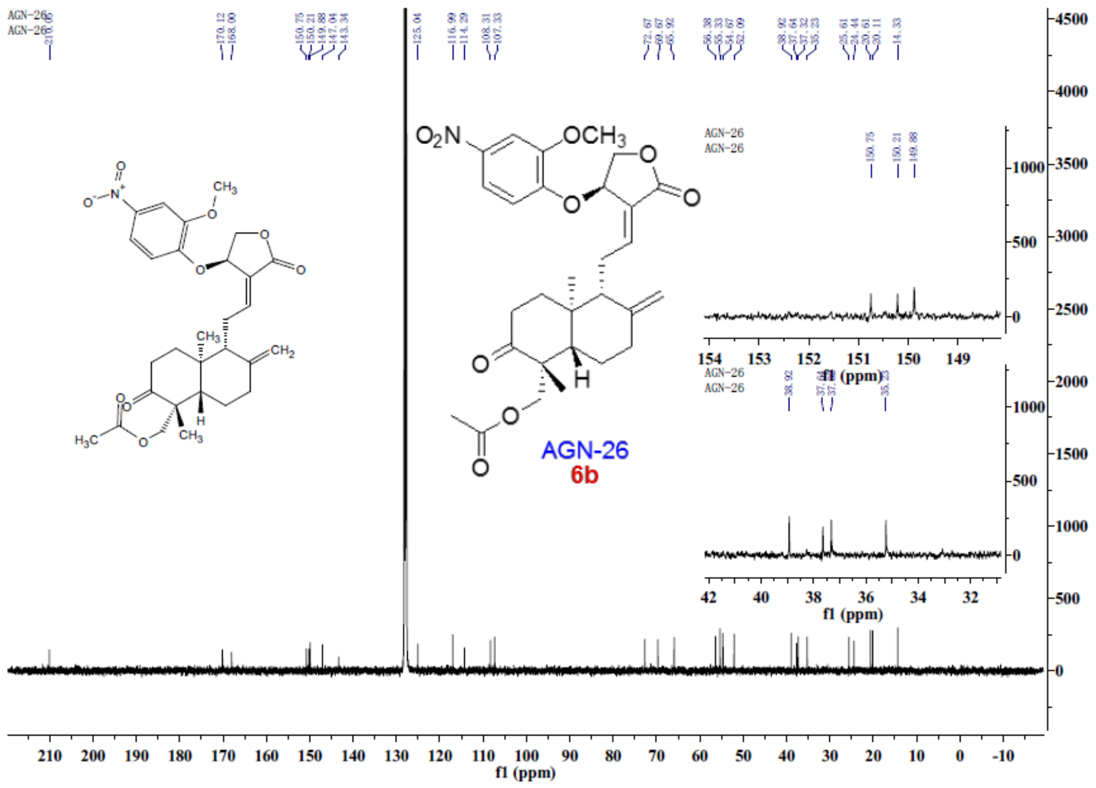

^{1}H NMR of **7b**

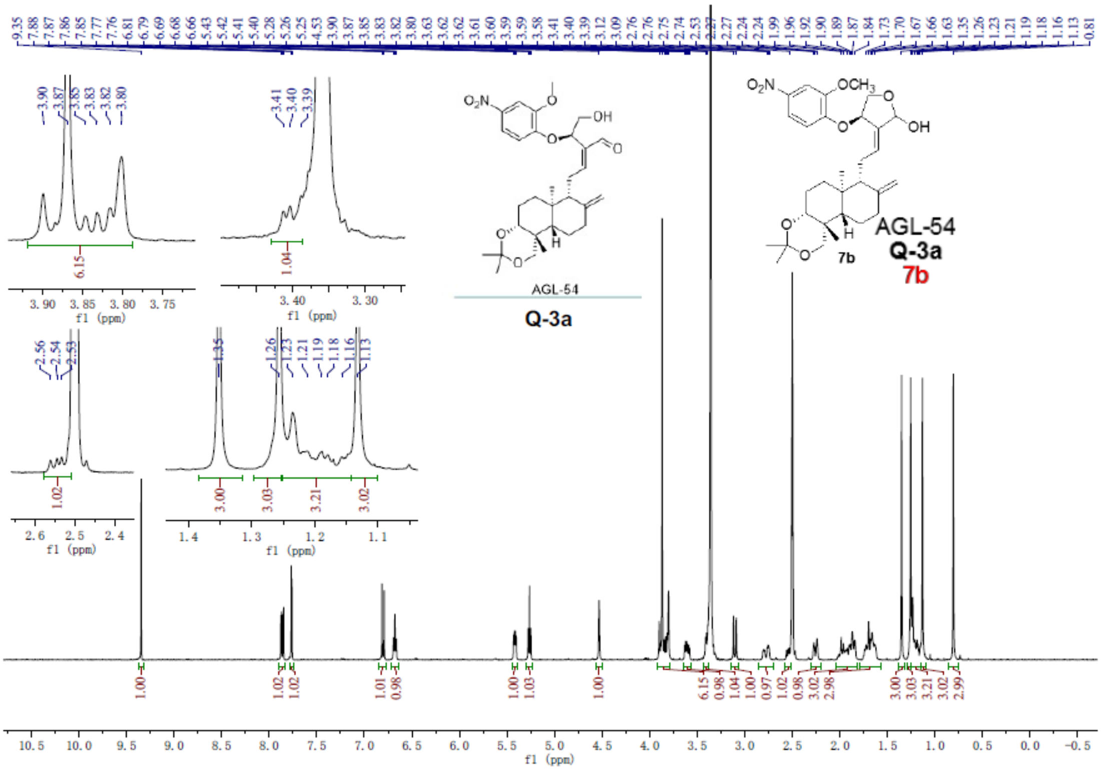

^{13}C NMR of **7b**

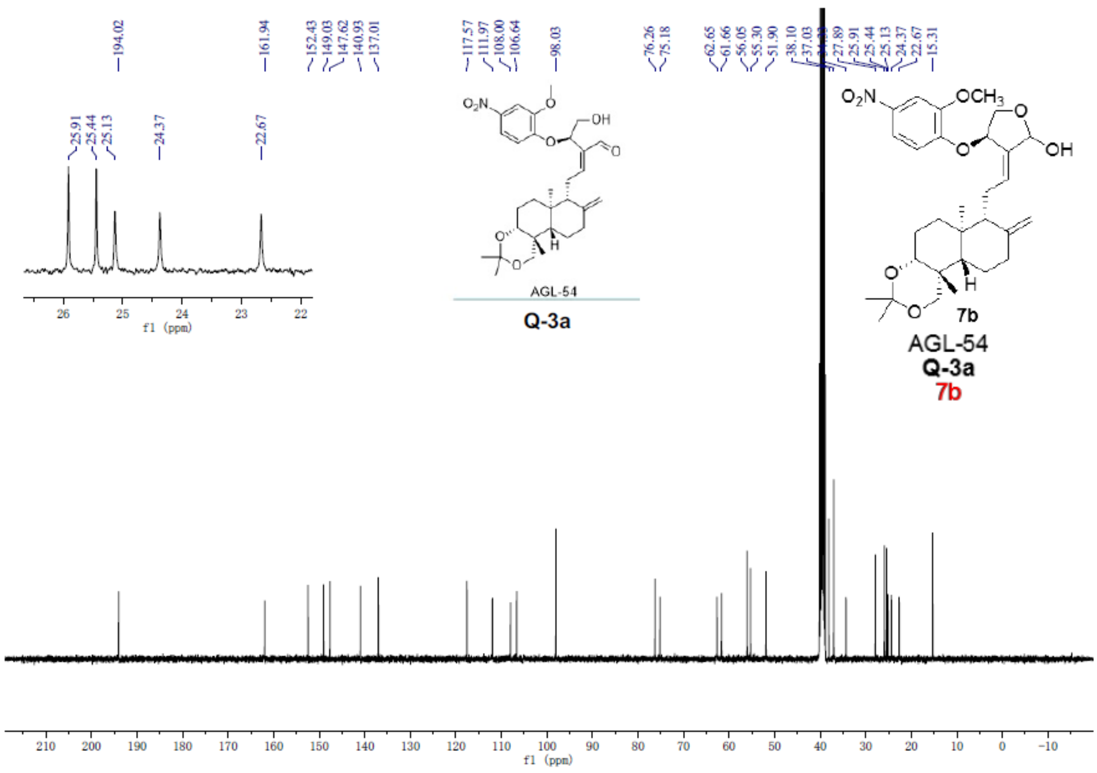

^{1}H NMR of **8b**

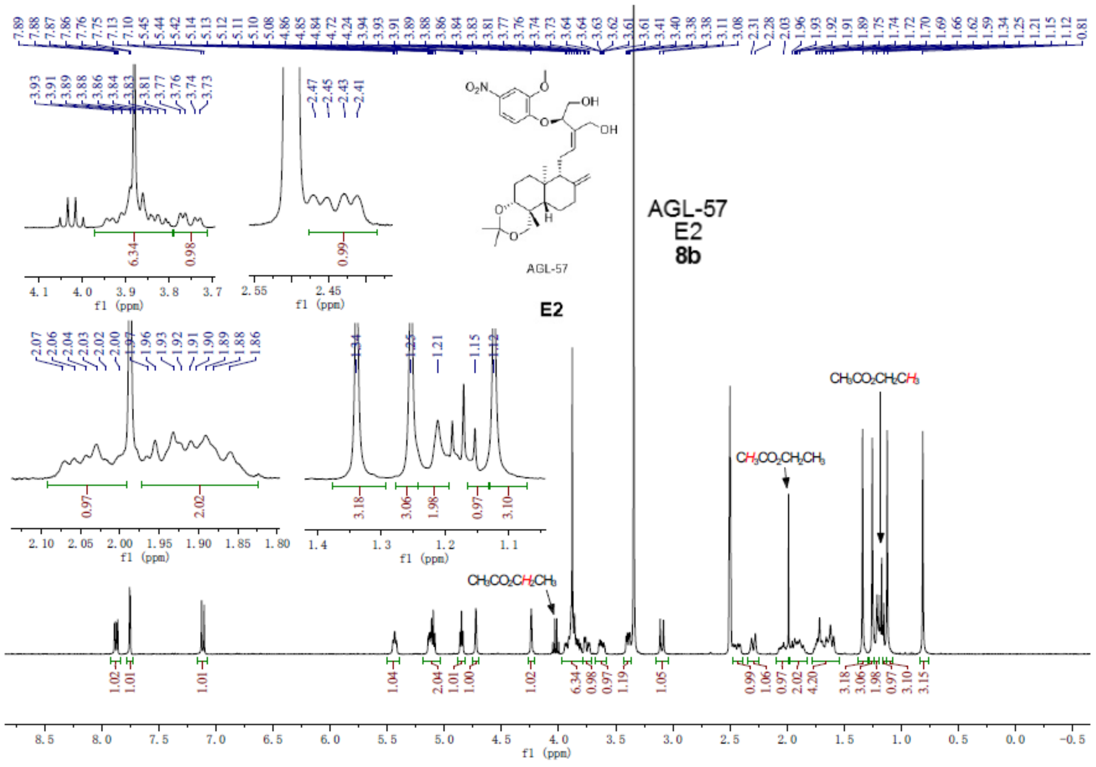

^{13}C NMR of **8b**

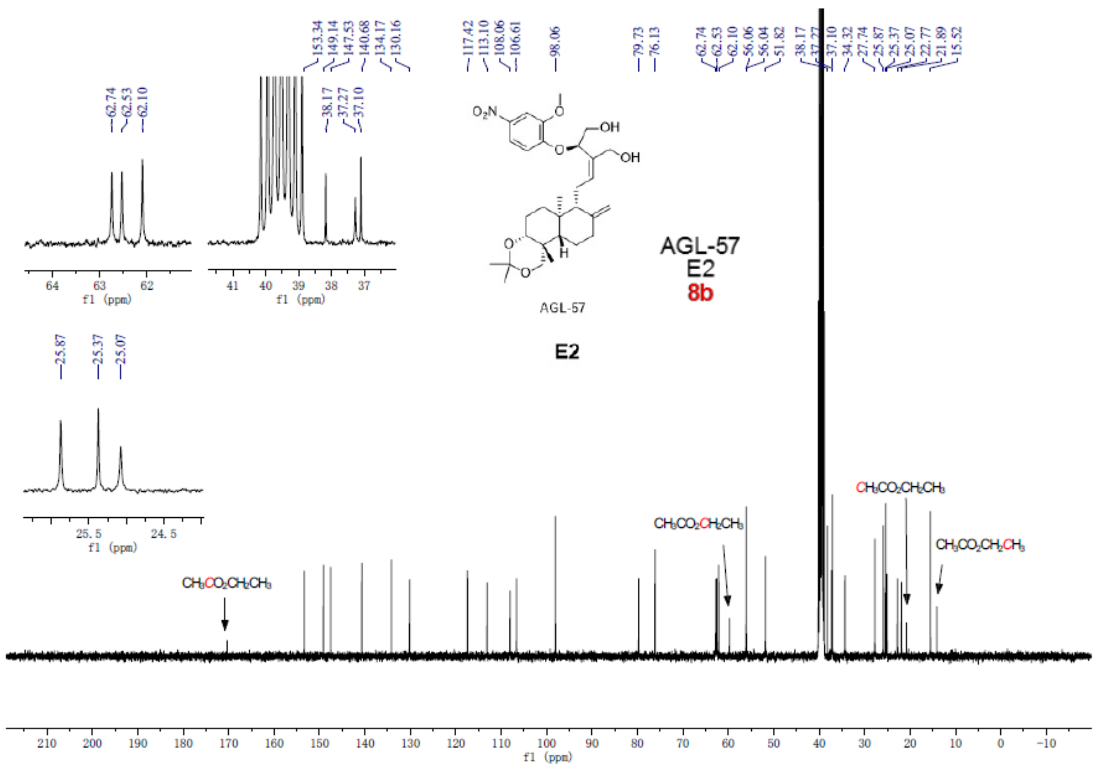

^{1}H NMR of **9b**

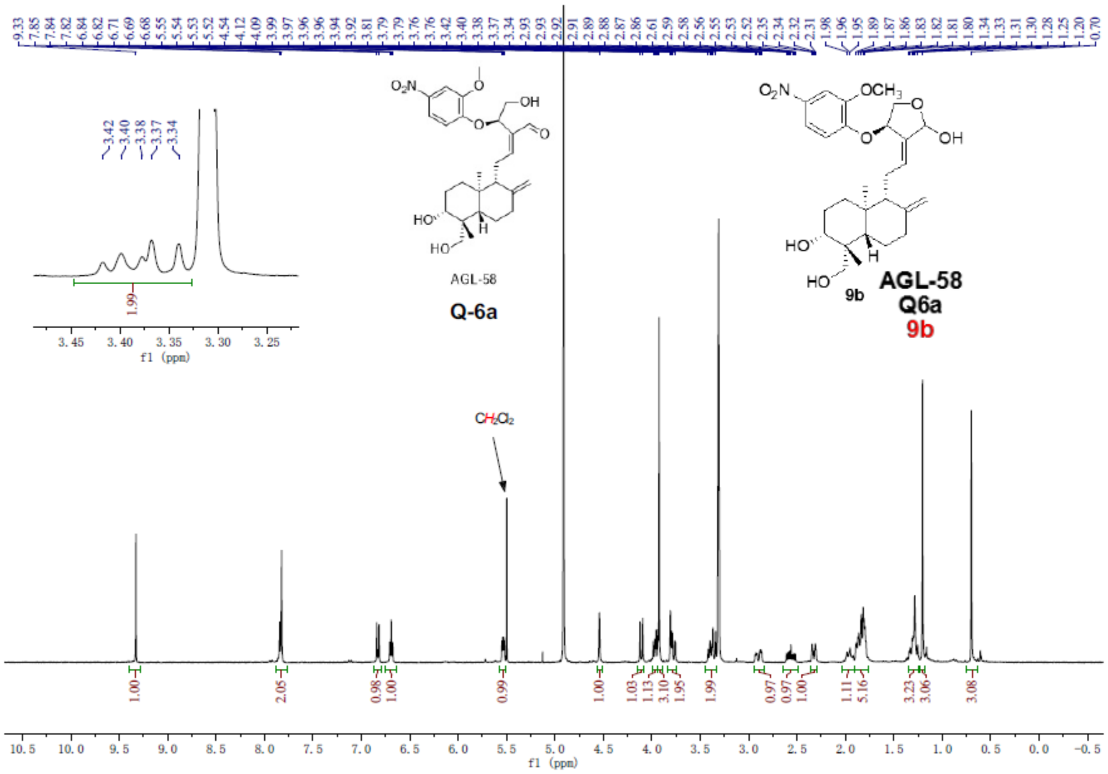

^{13}C NMR of **9b**

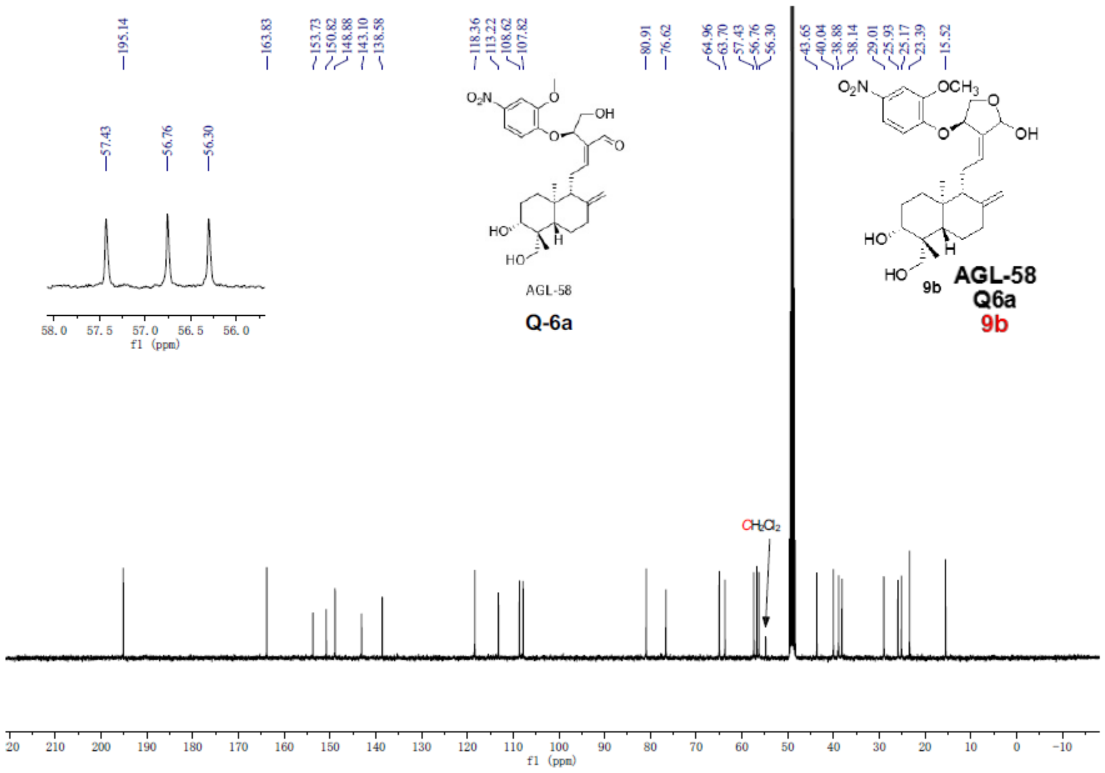

^{1}H NMR of **10b**

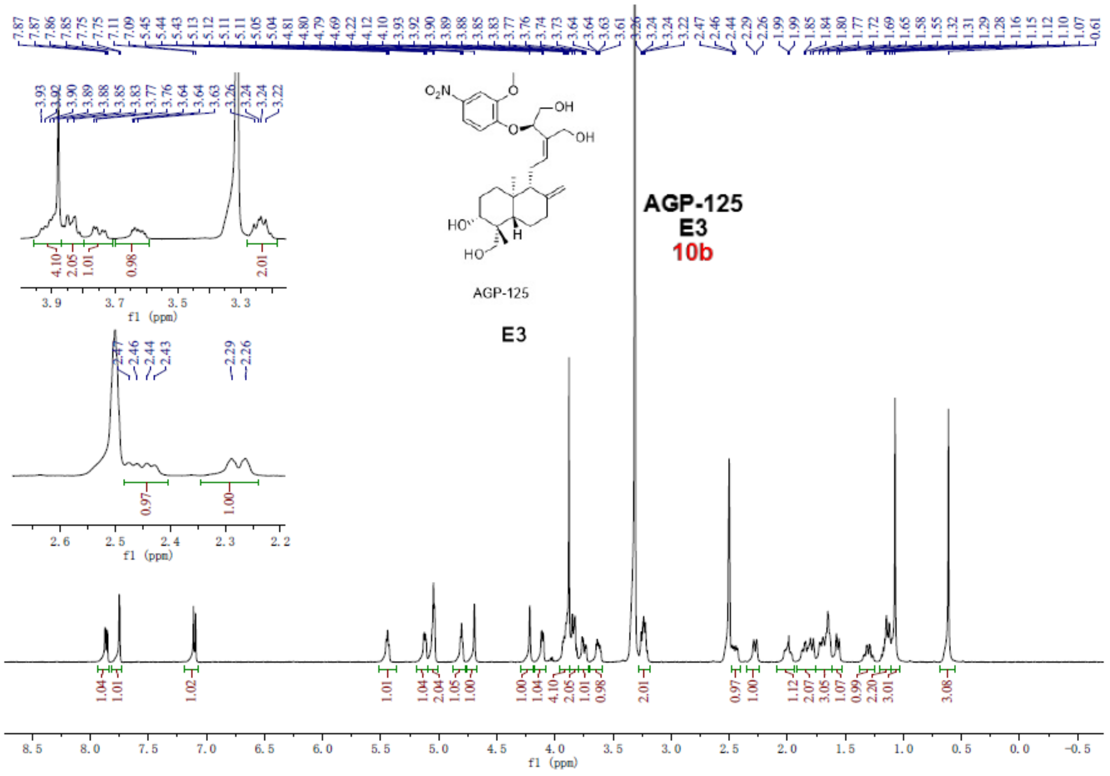

^{13}C NMR of **10b**

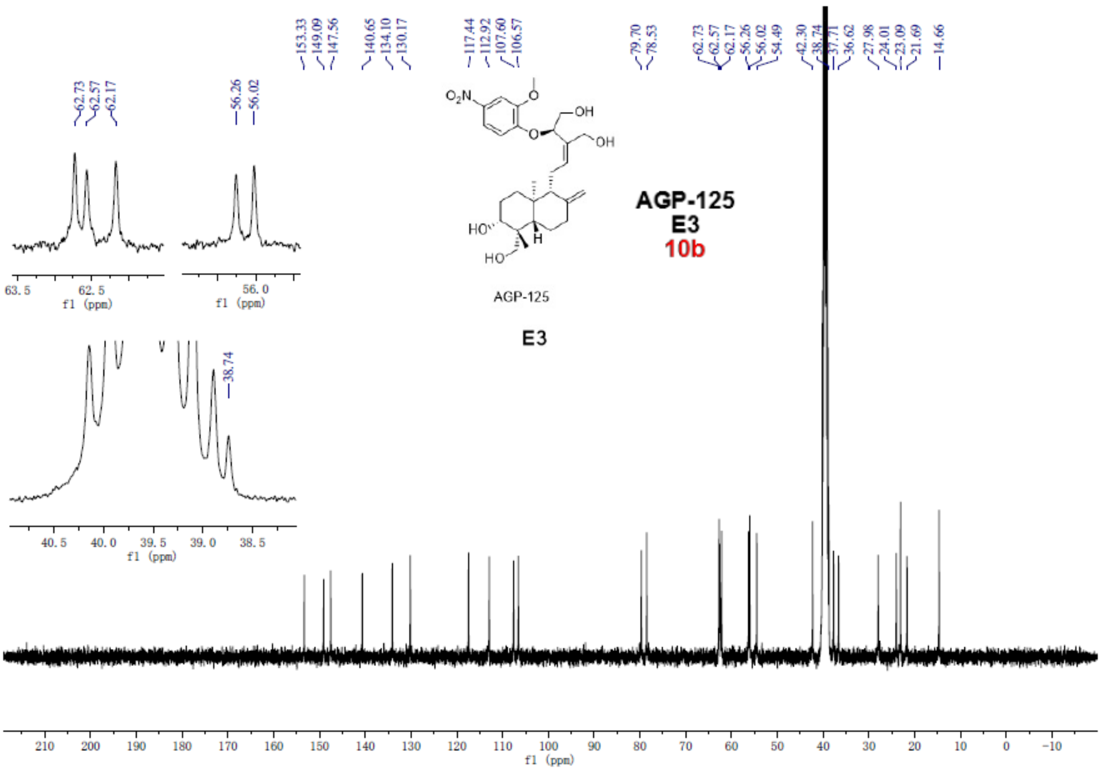

^{1}H NMR of **11ba**

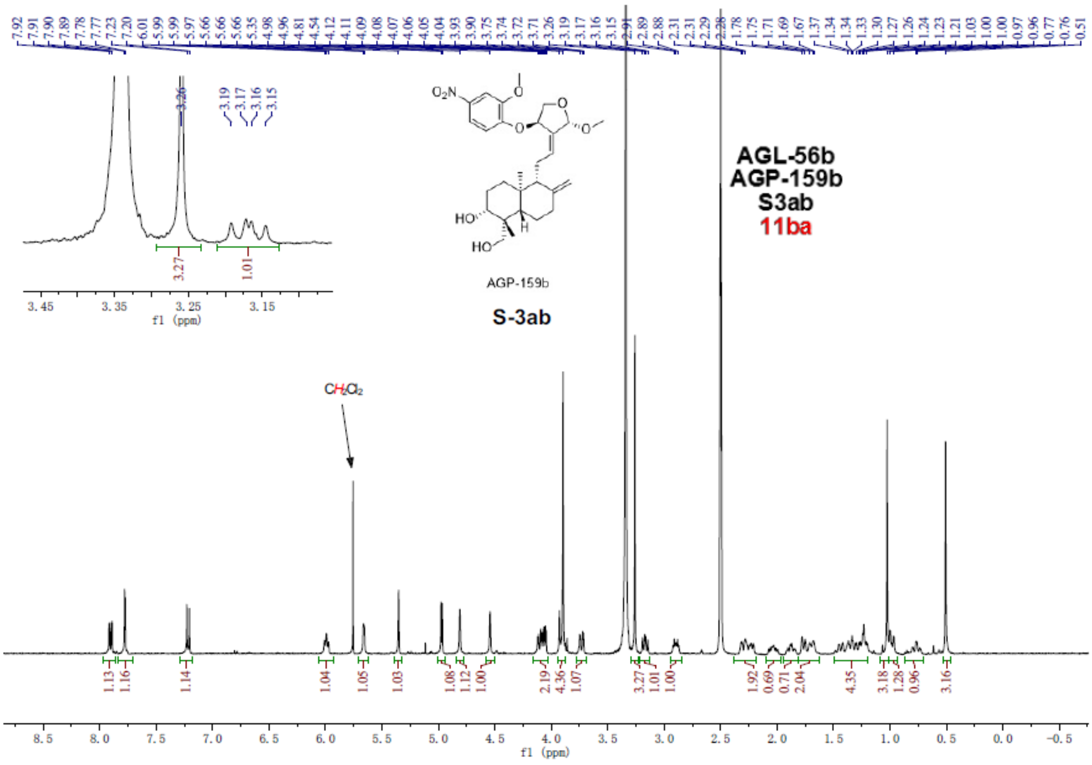

^{13}C NMR of **11ba**

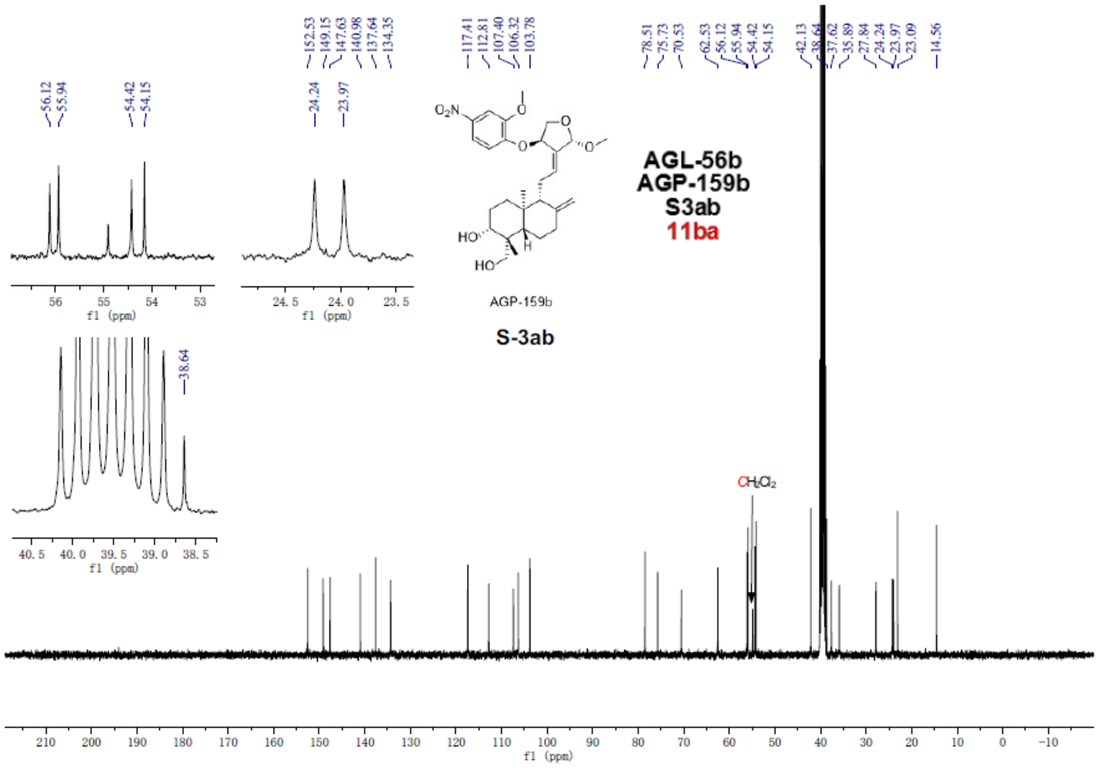

^{1}H NMR of **11bb**

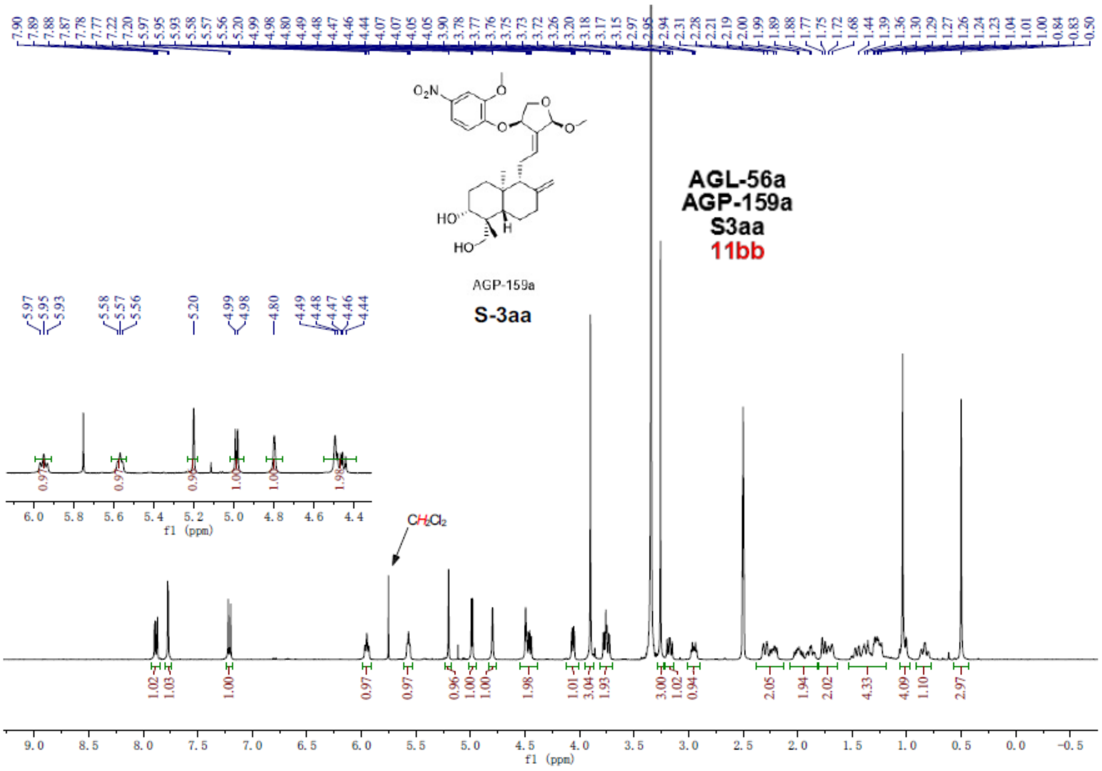

^{13}C NMR of **11bb**

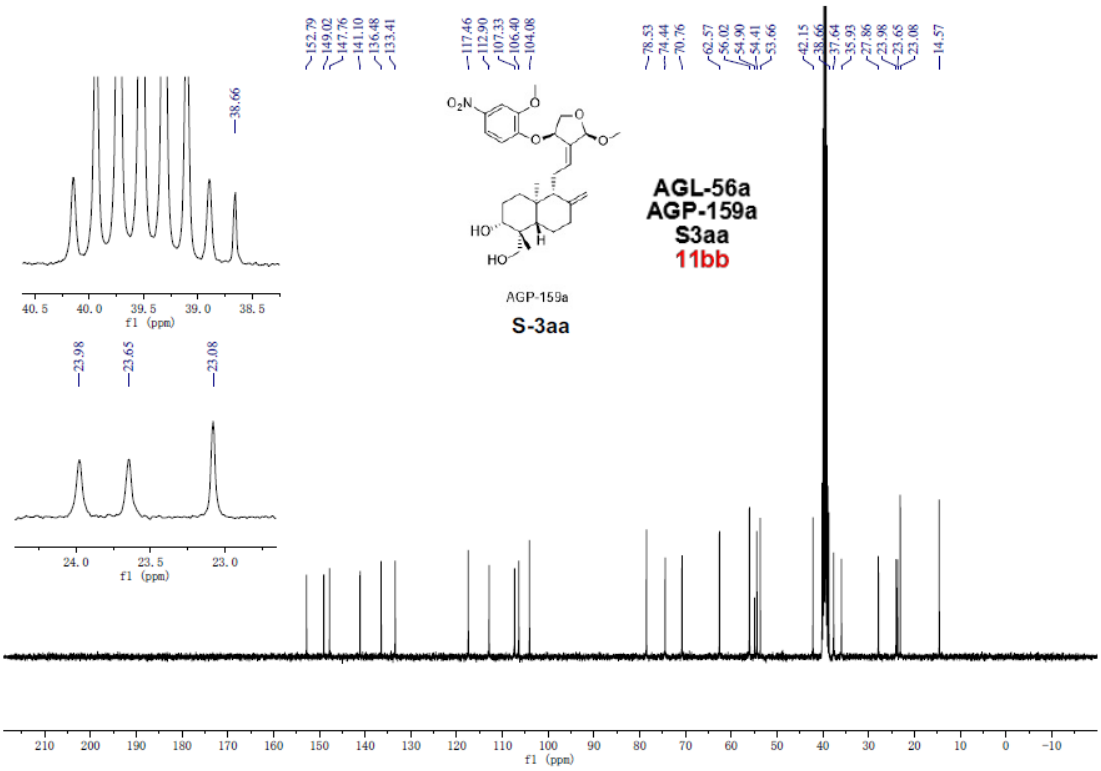

^{1}H NMR of **12ba**

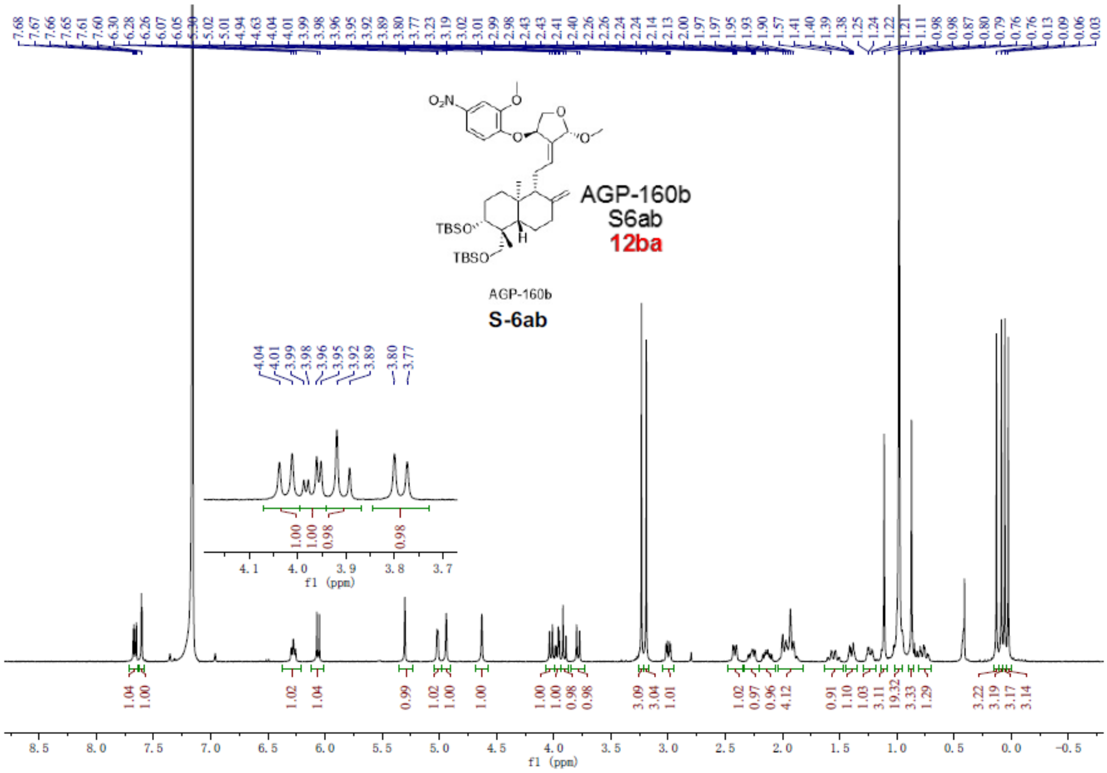

^{13}C NMR of **12ba**

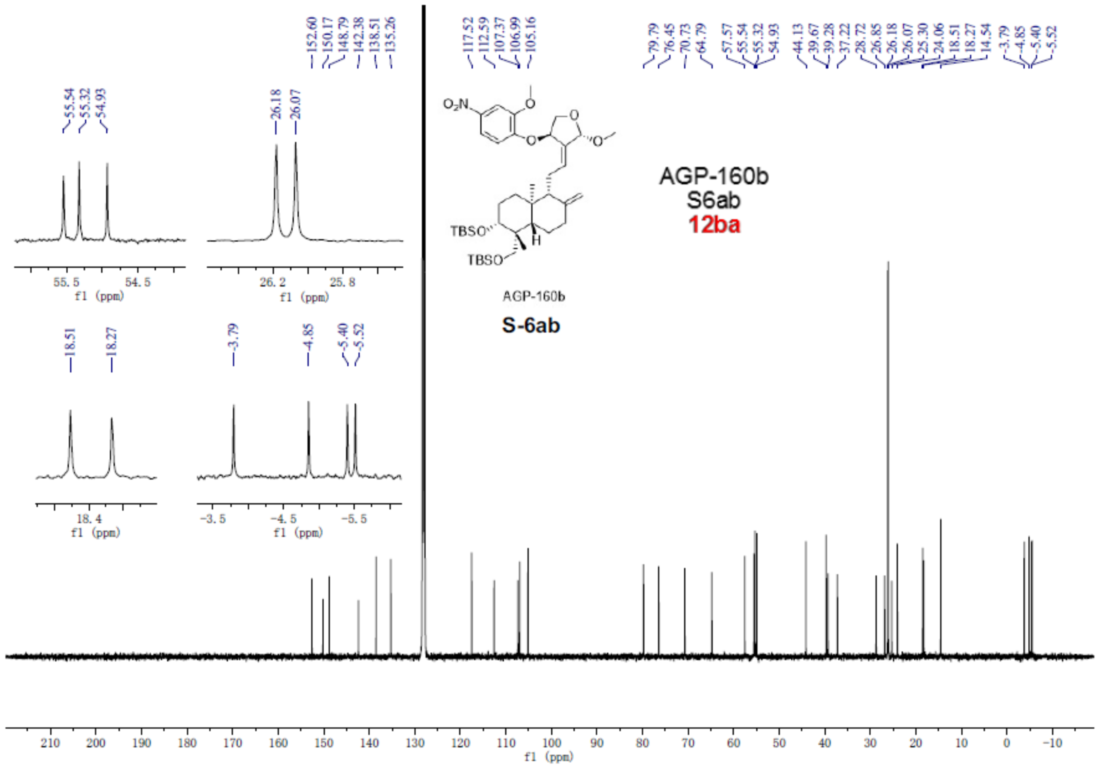

^{1}H NMR of **12bb**

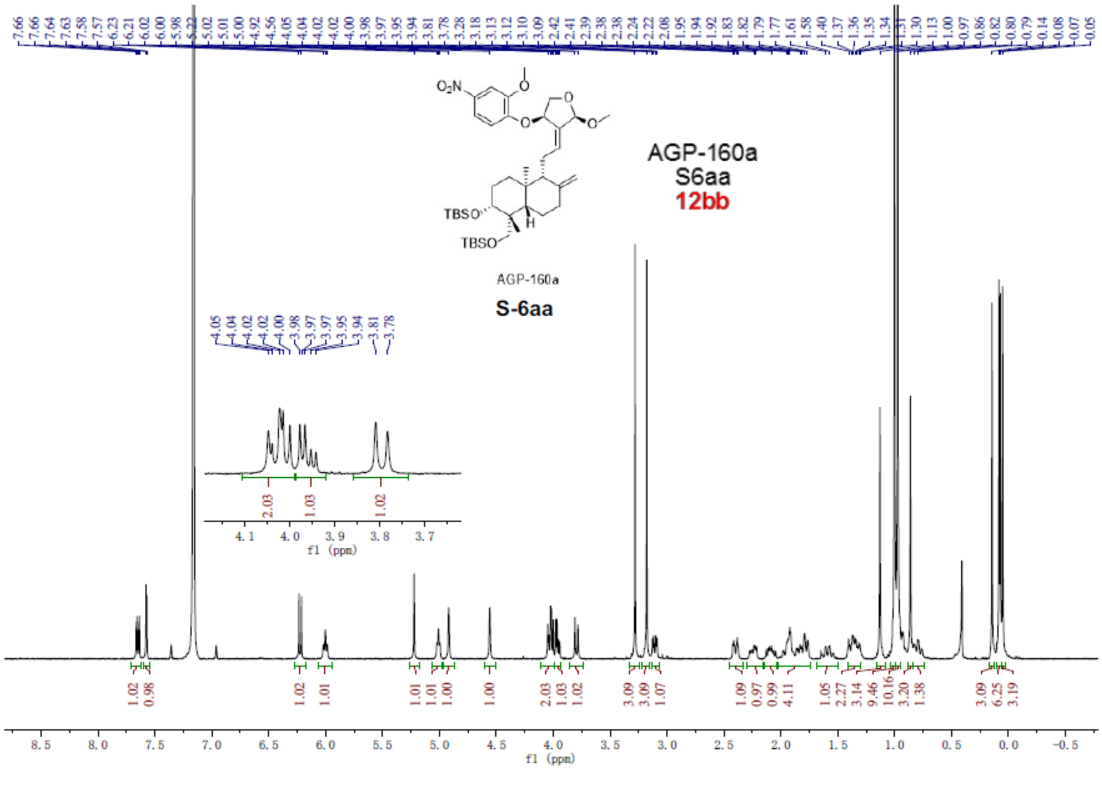

^{13}C NMR of **12bb**

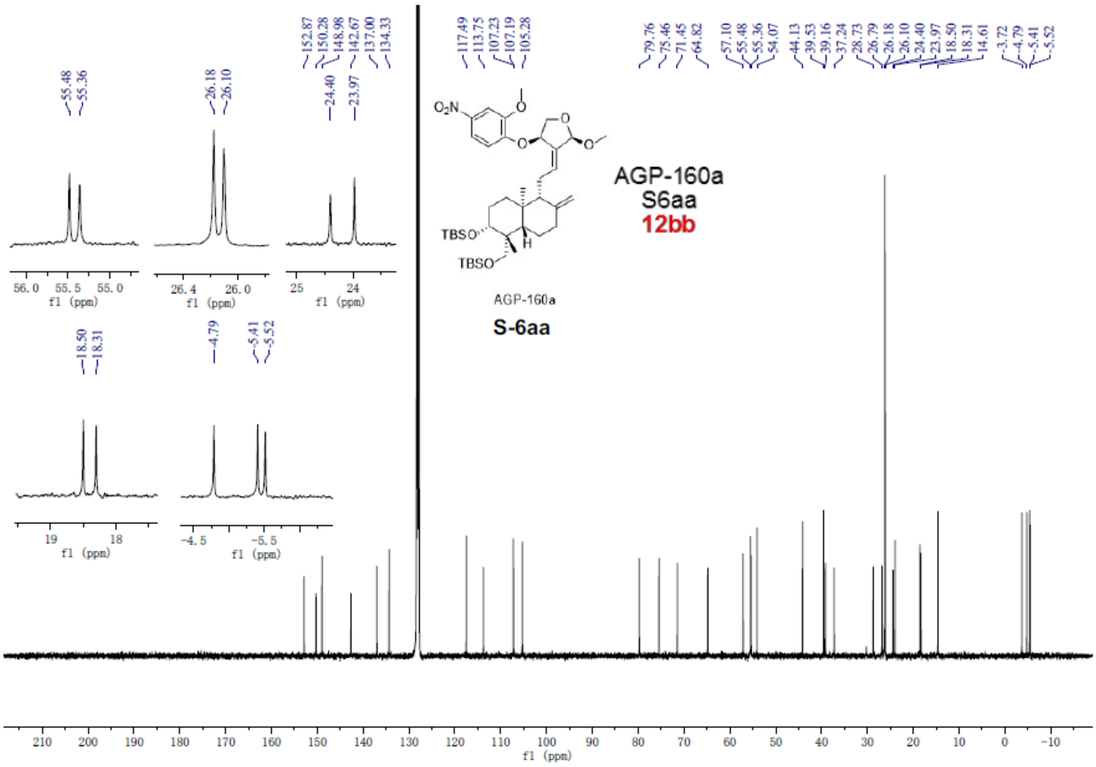

2b purity>98%

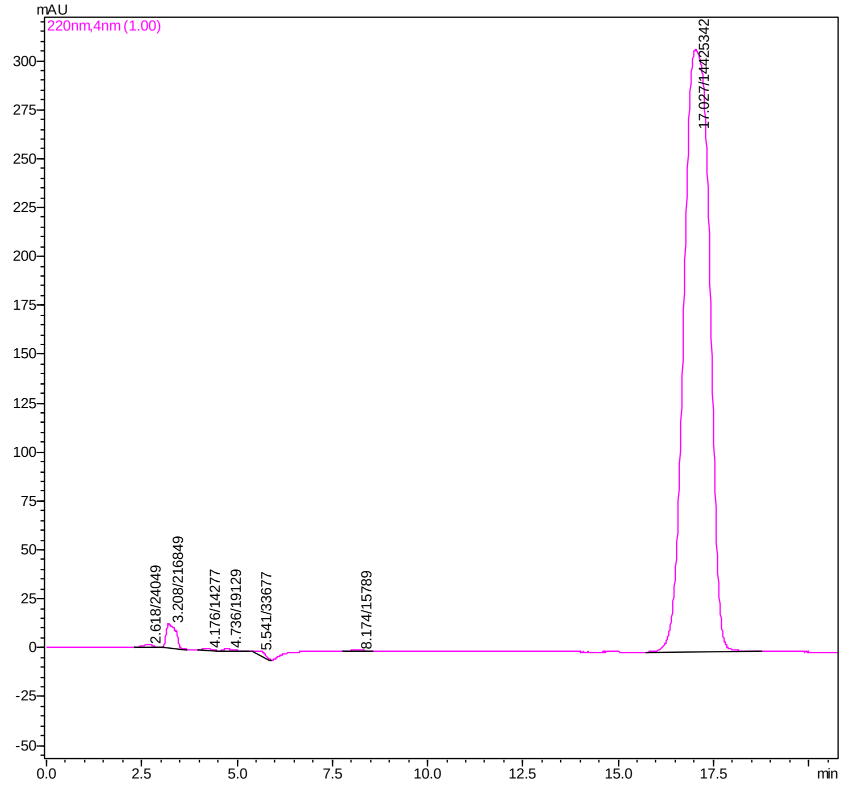

2a purity>98%

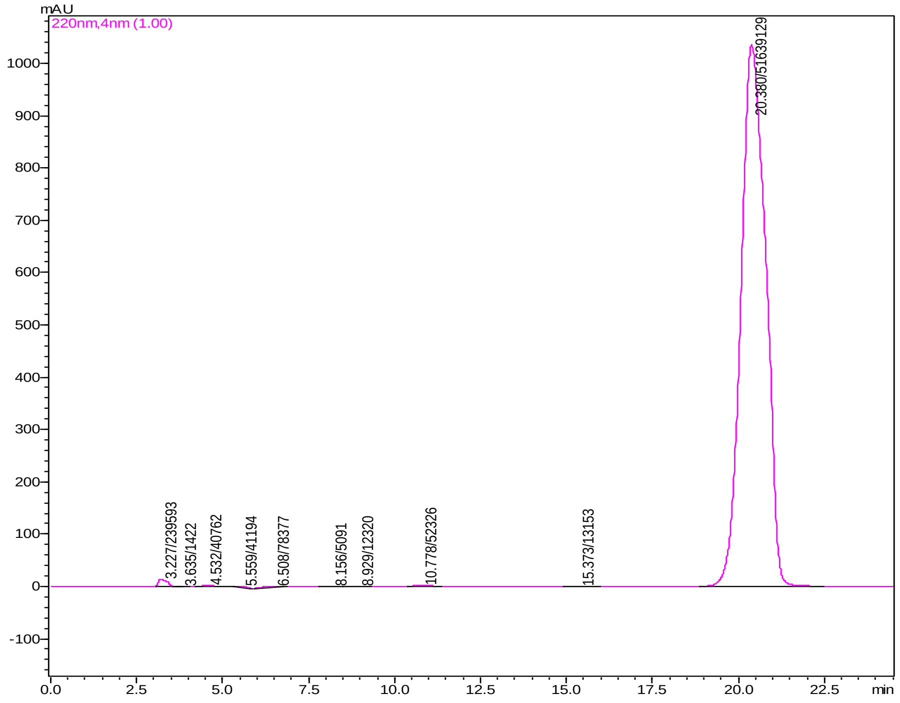

3a purity>98%

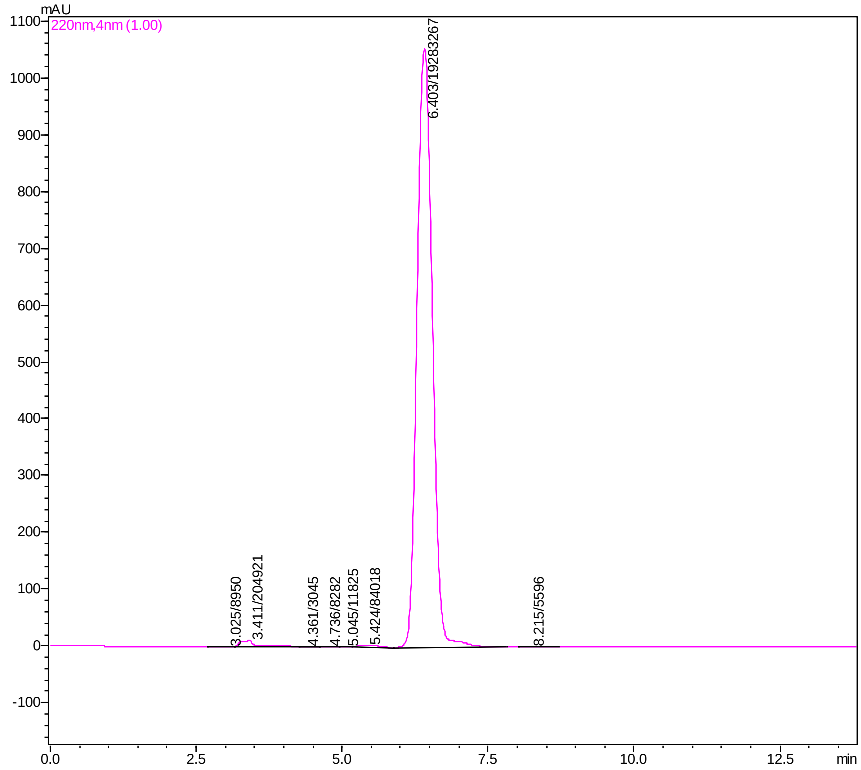

3bpurity>98%

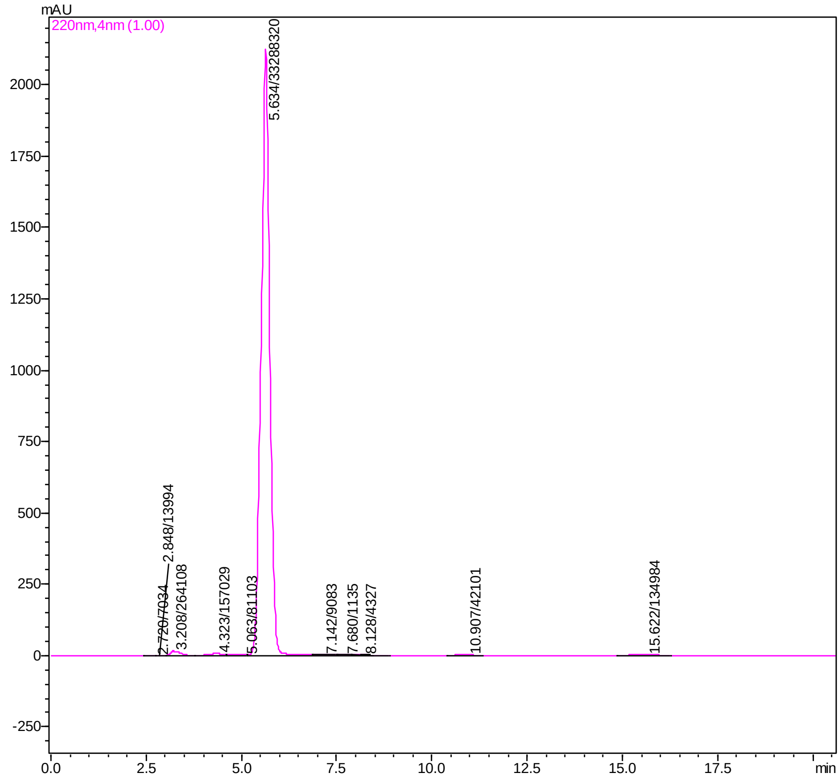

4a purity >94%

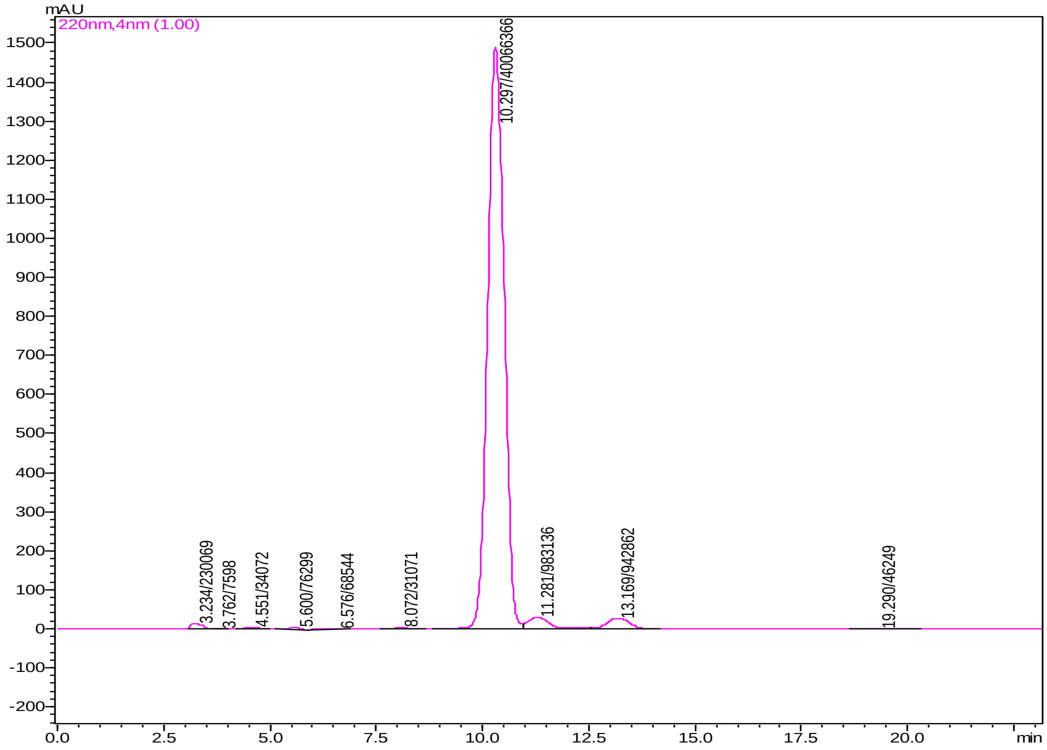

4bpurity>98%

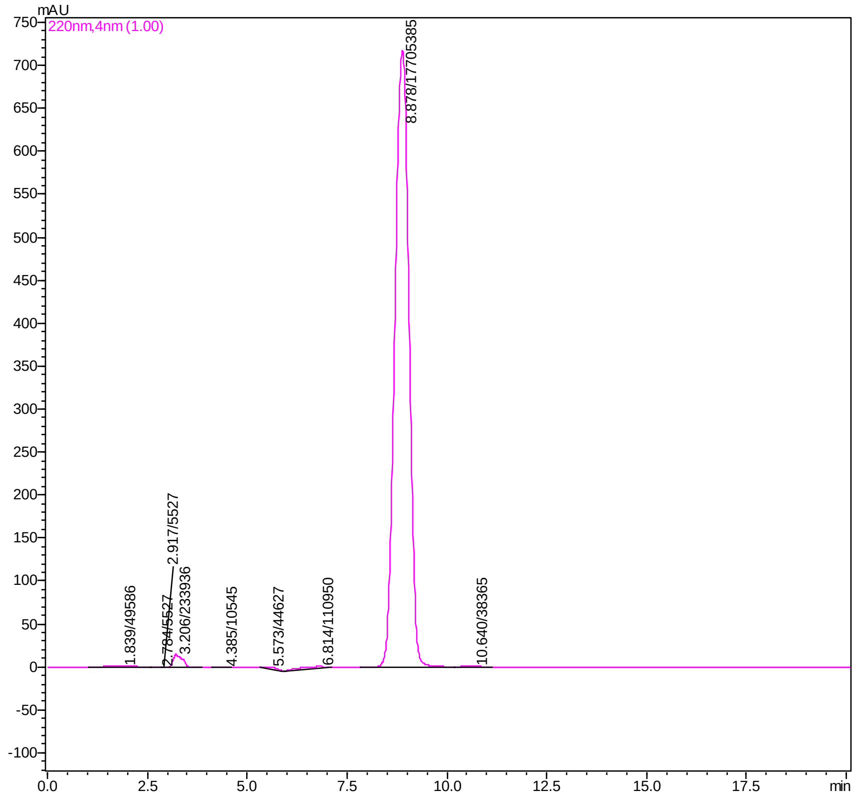

5a purity>98%

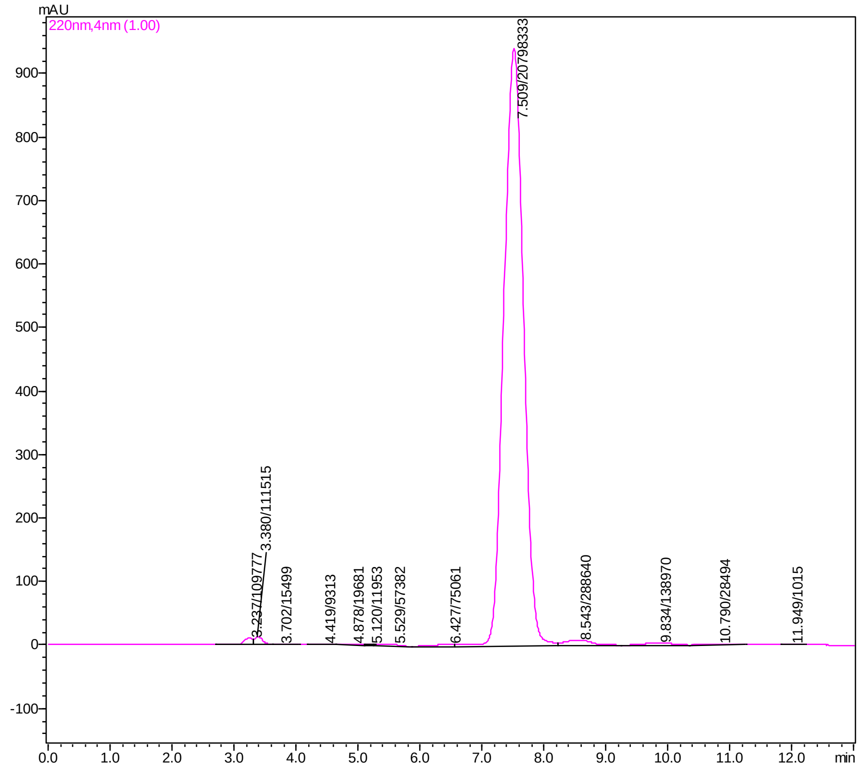

5b purity>98%

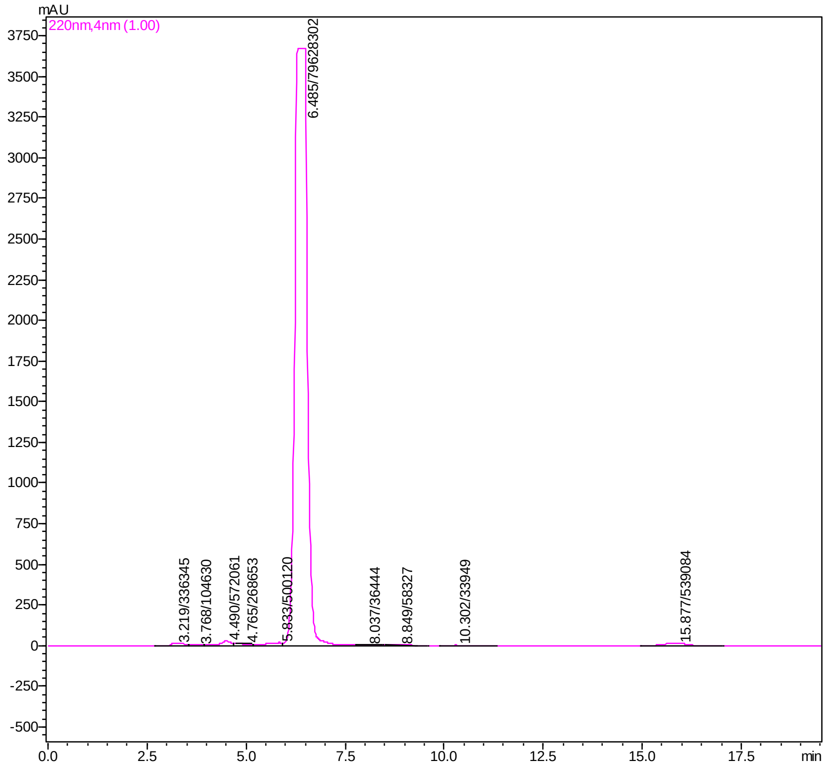

6b purity ≧ 98%

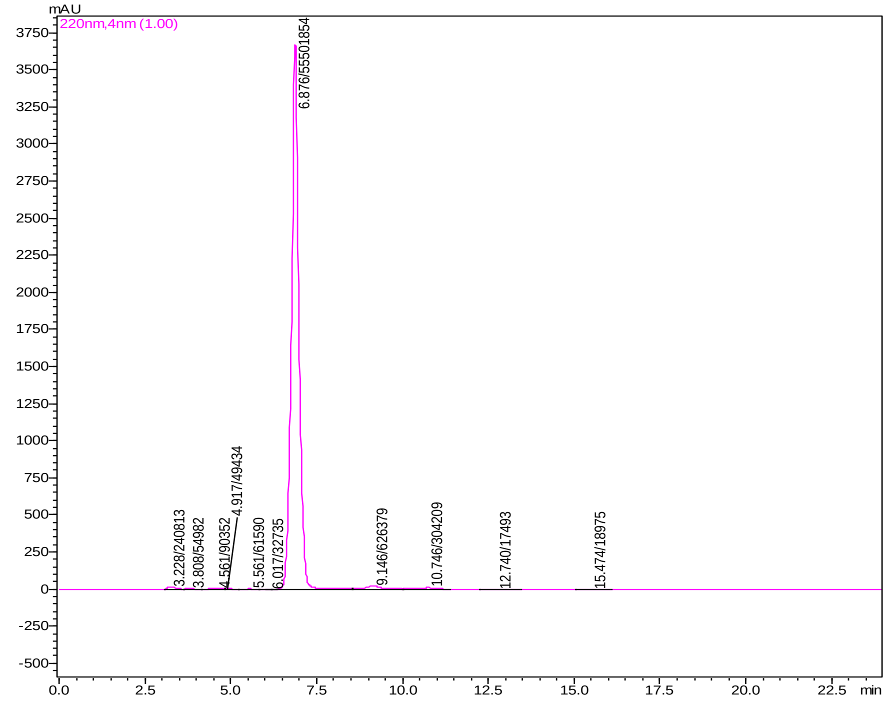

7bpurity >96%

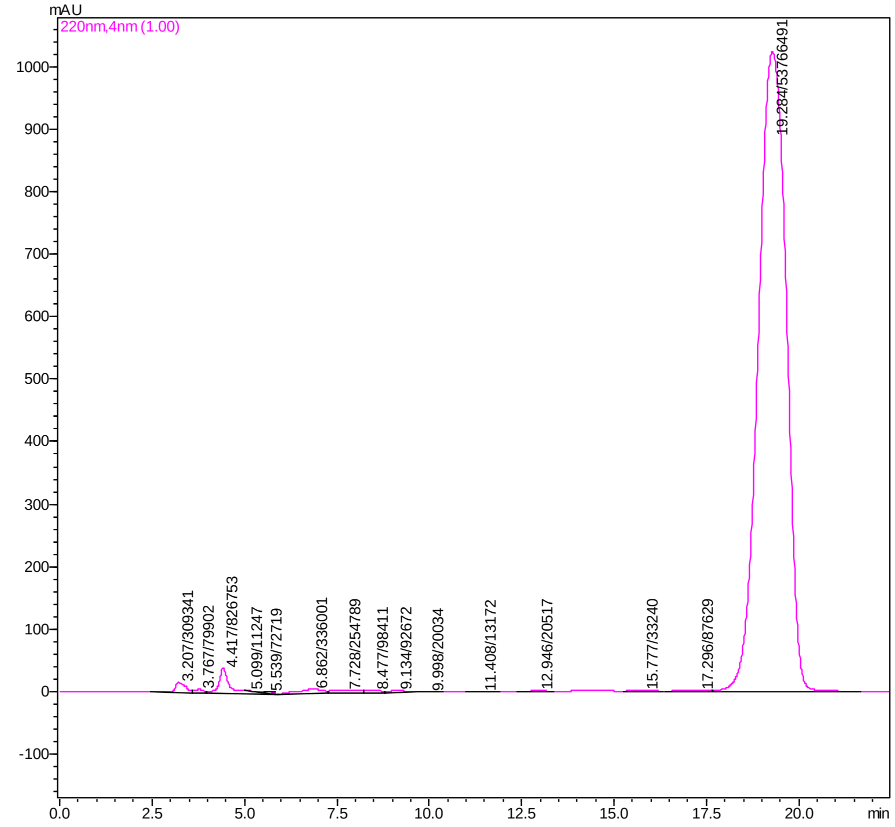

8b purity>97%

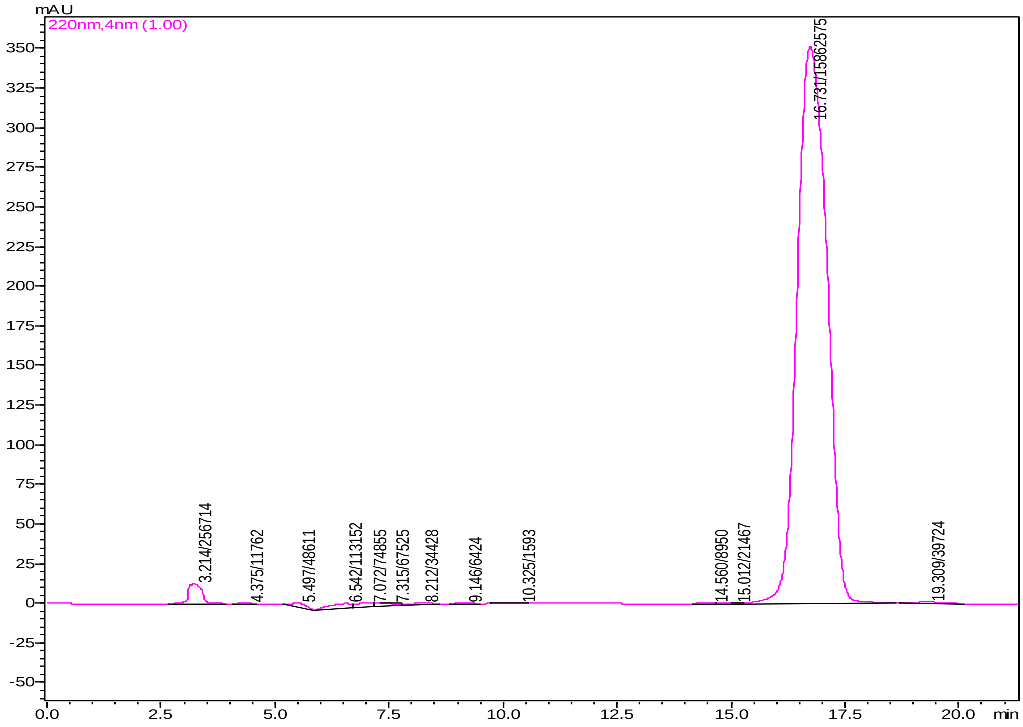

9b purity>98%

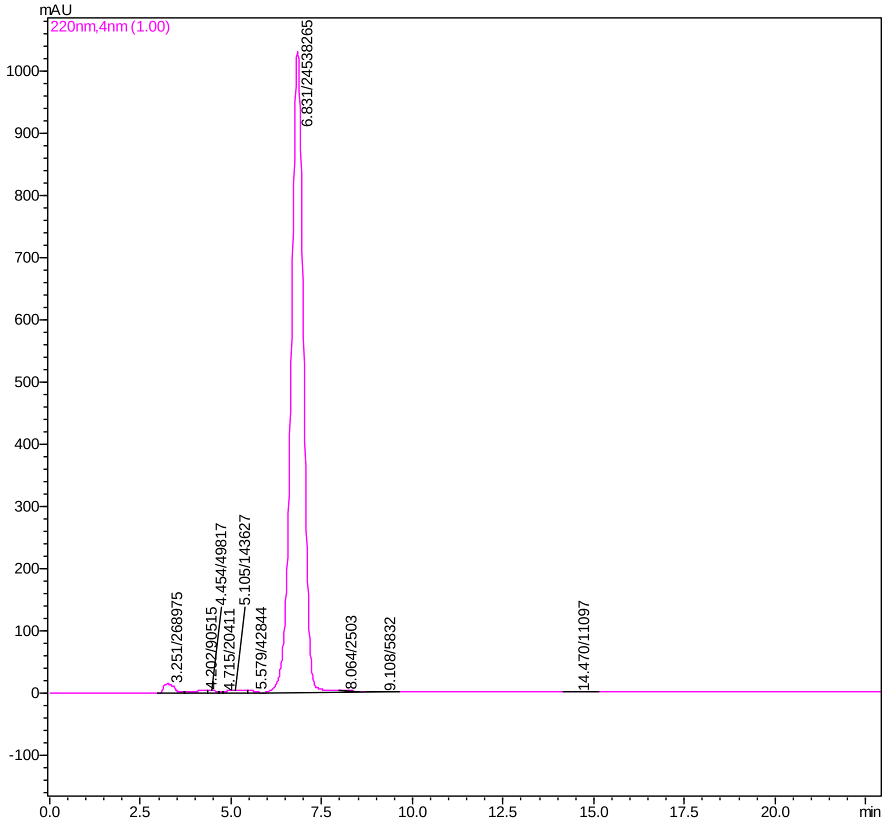

10b purity > 98%

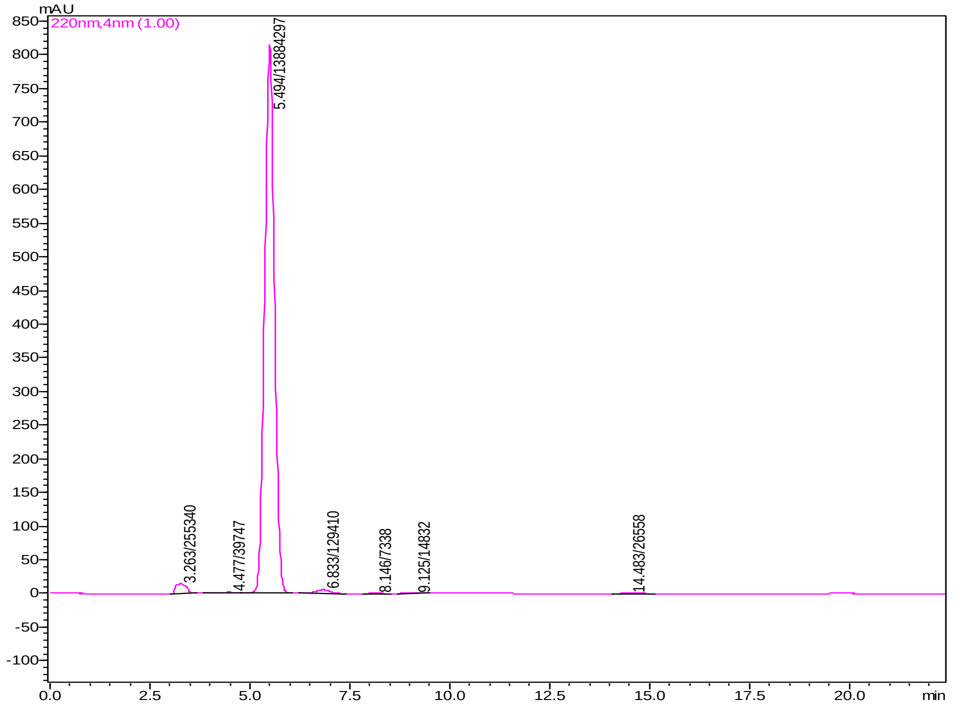

11ba purity ~ 95%

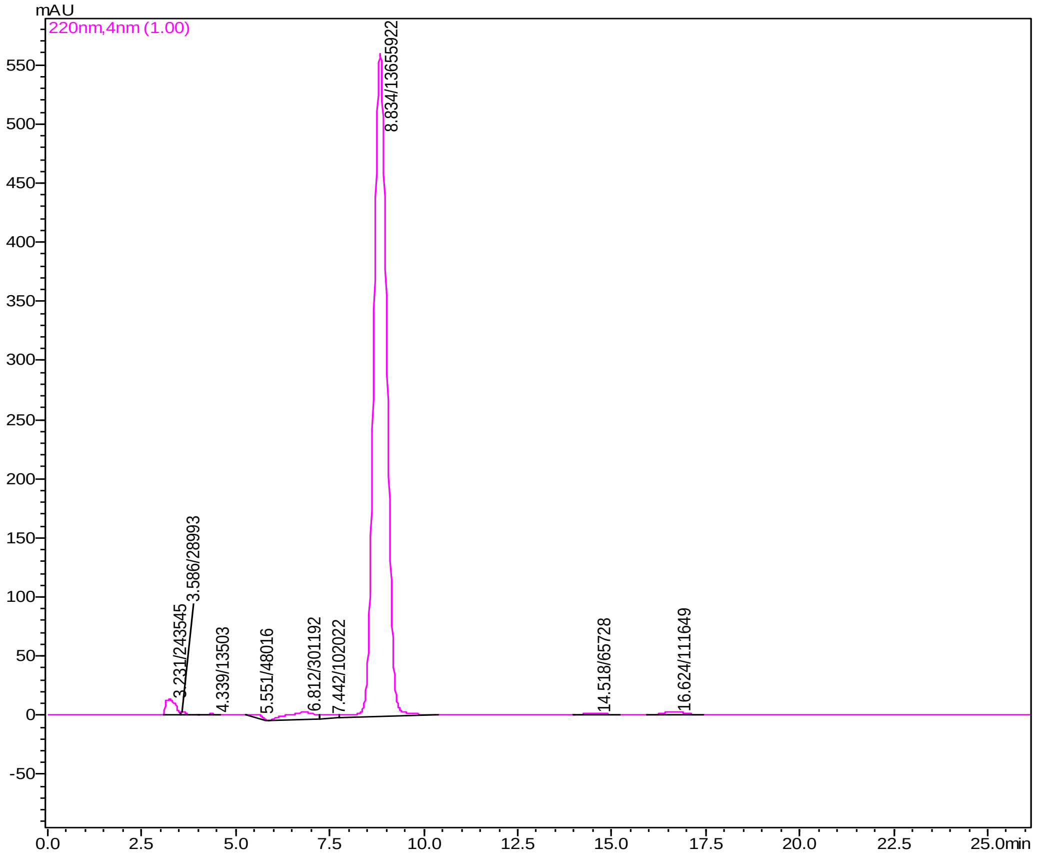

11bb purity ~ 95%

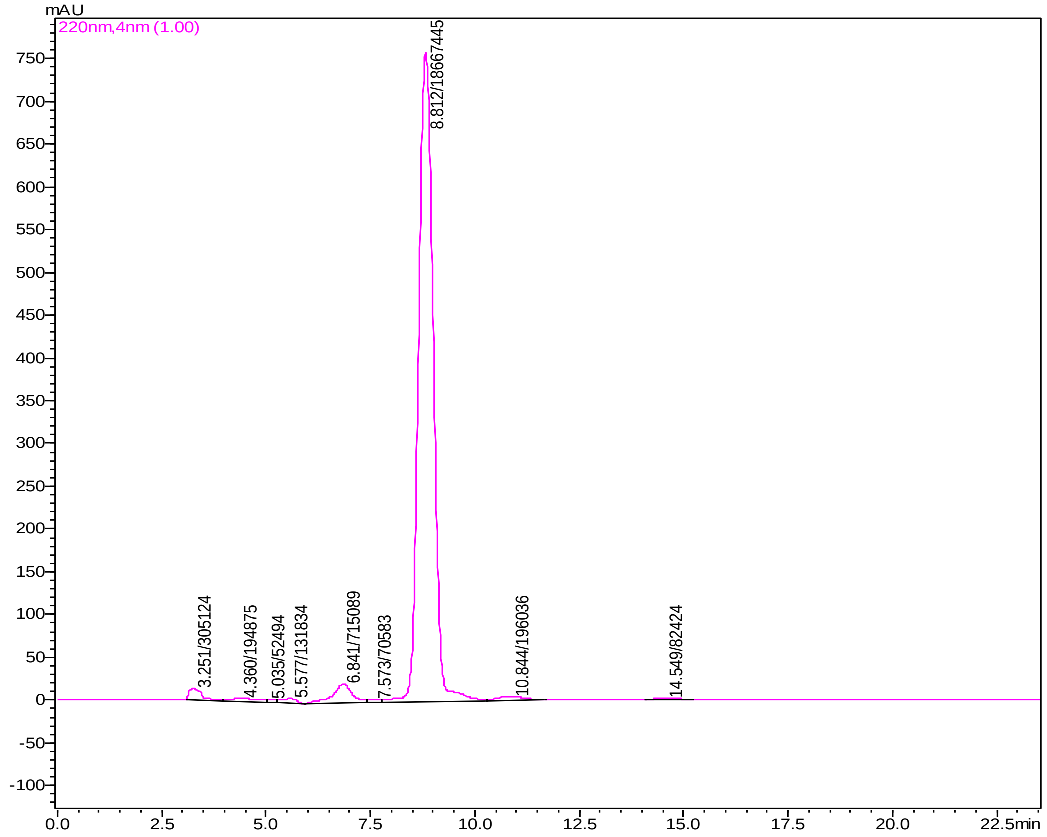

Biological evaluation

Cytotoxicity assay

Ten thousands cells/well were plated in 24-well plates. After drug treatment for 24 hours, cells were fixed and stained with 0.5% methylene blue in 50% ethanol for 2 hours at room temperature, followed by washing with tap water to remove excess color. Plates were dried and then suspended in 1% sarkosyl and incubate for 3 hours at room temperature. Cell growth was quantitated based on the amount of methylene blue adsorbed into cellular proteins measured by spectrophotometer (Molecular Devices) at 595 nm. IC_{50} was defined as the concentration of drug that inhibited cell growth by 50% after continuous drug exposure for 72 hours.

Confocal microscopy analysis

Briefly, AD-293 cells plated onto culture slides were treated as indicated in the figure for 4 hours. Then the cells were fixed with 4% paraformaldehyde at room temperature for 30 minutes and blocked with 3% BSA in PBS for 1 hour at room temperature. Cells were further incubated with primary antibody at optimal dilution in 3% BSA at 4 ˚C overnight followed by fluorescent conjugated secondary antibody at 1:500 dilution. Cells were then sealed in anti-fade reagent and examined under Leica TCS SP8 confocal microscope.

Detection of cytokine level

The levels of proinflammatory cytokines and chemokine in culture medium and

serum were detected by BioLedgendLEGENDplex™ bead-based immunoassays following the manufacturer's recommendations on BD Accuri™ C6 flow cytometer.

Table S1. Primer sequences used in the present study.

	Forward primer (5’-3’)	Reverse primer (5’-3’)
IL-6	CCTGAACCTTCCAAAGATGG C	TTCACCAGGCAAGTCTCCTCA
IL-18	GTAACCCGTTGAACCCCATT	CCATCCAATCGGTAGTAGCG
TNF-α	GACGTGGAACTGGCAGAAGA G	TTGGTGGTTTGTGAGTGTGAG
IFN-β	ATGACCAACAAGTGTCTCCTC C	GGAATCCAAGCAAGTTGTAG CTC
18s rRNA	GTAACCCGTTGAACCCCATT	CCATCCAATCGGTAGTAGCG

References

1 Lim, J. C. *et al.* Andrographolide and its analogues: versatile bioactive molecules for combating inflammation and cancer. *Clin Exp Pharmacol Physiol* **39,** 300-310, (2012).
